# Supplementary material for: Estimated impact of the 2020 economic downturn on under-5 mortality for 129 countries
Source: PLoS One. 2022 Feb 23;17(2):e0263245. doi: 10.1371/journal.pone.0263245 (PMC8865697; doi:10.1371/journal.pone.0263245)
Supplement: S1 Appendix — (ZIP) [file pone.0263245.s001.zip › S1 Appendix.pdf]

S1 Appendix

Descriptive Statistics of variables used for the analysis.

	Year														
	1990	1991	1992	1993	1994	1995	1996	1997	1998	1999	2000	2001	2002	2003	2004
**Under-five mortality (deaths under age 5 per 1,000 live births)**															
Mean	102.49	100.82	99.06	97.14	95.01	92.66	90.13	87.48	84.79	82.08	89.90	88.08	82.79	80.78	70.77
Standard Deviation	71.62	72.23	72.33	71.64	70.09	67.89	65.40	63.02	60.91	59.03	58.80	58.30	55.66	53.62	52.09
Share of missing observations	0.00	0.00	0.00	0.00	0.00	0.00	0.00	0.00	0.00	0.00	0.00	0.00	0.00	0.00	0.00
**GDP per capita constant 2010$**															
Mean	2,454	2,431	2,334	2,356	2,331	2,346	2,402	2,475	2,509	2,591	2,451	2,462	2,619	2,614	2,806
Standard Deviation	2,444	2,444	2,422	2,381	2,369	2,338	2,346	2,430	2,457	2,430	2,430	2,313	2,480	2,531	2,717
Share of missing observations	13.18	13.18	12.40	11.63	10.85	8.53	8.53	7.75	7.75	6.98	5.43	4.65	3.88	3.88	3.88
**Physicians (per 1,000 people)**															
Mean	0.96	1.08	1.04	0.95	1.07	1.03	1.02	1.05	1.12	1.09	0.78	0.79	0.87	0.81	0.76
Standard Deviation	1.30	1.41	1.39	1.33	1.40	1.32	1.31	1.29	1.36	1.34	1.22	1.23	1.26	1.22	1.06
Share of missing observations	30.23	62.02	61.24	46.51	62.02	42.64	53.49	48.06	62.02	63.57	52.71	58.91	62.02	62.02	31.78
**Electric power consumption (kWh per capita)**															
Mean	978	963	921	881	889	898	924	933	972	981	824	785	904	864	926
Standard Deviation	1,412	1,394	1,327	1,223	1,201	1,167	1,186	1,177	1,201	1,165	1,165	1,103	1,179	1,146	1,142
Share of missing observations	37.21	36.43	35.66	35.66	35.66	34.88	34.88	34.88	34.88	34.88	33.33	33.33	33.33	33.33	33.33
**Proportion of seats held by women in national parliaments (%)**															
Mean	4.06	4.80	5.23	5.85	6.52	7.44	7.99	8.25	9.12	9.32	9.48	10.28	11.69	12.44	12.44
Standard Deviation	10.15	10.15	10.09	10.17	10.20	10.33	10.21	7.33	7.72	7.51	8.08	7.90	9.12	9.22	9.03
Share of missing observations	100.00	100.00	100.00	100.00	100.00	100.00	100.00	20.16	18.60	24.03	18.60	14.73	18.60	10.85	7.75
**Total fertility (live births per woman)**															
Mean	4.79	4.70	4.61	4.52	4.43	4.34	4.25	4.17	4.09	4.02	4.36	4.36	4.21	4.20	3.92
Standard Deviation	1.64	1.65	1.66	1.66	1.67	1.68	1.69	1.70	1.70	1.71	1.66	1.59	1.60	1.60	1.58
Share of missing observations	0.00	0.00	0.00	0.00	0.00	0.00	0.00	0.00	0.00	0.00	0.00	0.00	0.00	0.00	0.00
**Immunization, DPT (% of children ages 12-23 months)**															
Mean	74.71	71.89	70.88	71.15	72.64	74.45	74.19	74.44	74.89	74.78	72.44	72.99	73.59	76.32	78.10
Standard Deviation	21.56	22.22	21.69	21.45	21.11	20.55	20.82	22.09	22.02	21.88	20.66	20.91	19.28	18.52	18.24
Share of missing observations	17.05	16.28	5.43	3.88	3.88	3.88	3.88	3.88	3.88	2.33	2.33	2.33	1.55	1.55	1.55
**Immunization, measles (% of children ages 12-23 months)**															
Mean	73.39	71.53	69.77	71.77	72.50	74.39	74.59	74.66	75.41	75.68	72.34	73.54	74.62	75.83	77.82
Standard Deviation	19.28	20.41	20.28	20.21	19.10	19.18	19.59	20.38	20.66	19.90	19.64	19.69	18.69	17.64	18.98
Share of missing observations	17.05	16.28	5.43	3.88	3.88	3.88	3.88	3.88	3.88	2.33	2.33	2.33	1.55	1.55	1.55

	Year															
	2005	2006	2007	2008	2009	2010	2011	2012	2013	2014	2015	2016	2017	2018	2019	2020
**Under-five mortality (deaths under age 5 per 1,000 live births)**																
Mean	76.26	73.73	72.08	66.84	64.12	66.69	57.82	57.17	53.73	53.03	43.52	42.07	40.74	39.52	38.37	37.29
Standard Deviation	49.73	47.46	46.79	44.43	41.74	42.26	39.49	37.97	34.94	35.01	32.34	31.32	30.41	29.57	28.81	28.09
Share of missing observations	0.00	0.00	0.00	0.00	0.00	0.00	0.00	0.00	0.00	0.00	0.00	0.00	0.00	0.00	0.00	0.00
**GDP per capita constant 2010$**																
Mean	2,861	2,941	2,995	3,144	3,145	2,973	3,209	3,387	3,362	3,485	3,738	3,782	3,824	3,940	3,996	3,957
Standard Deviation	2,998	3,122	3,453	3,419	3,387	3,306	3,178	3,406	3,225	3,342	3,163	3,148	3,228	3,237	3,273	2,835
Share of missing observations	3.88	3.88	3.88	3.10	3.10	2.33	3.10	3.88	3.88	3.88	4.65	5.43	5.43	6.20	10.85	100.00
**Physicians (per 1,000 people)**																
Mean	0.82	0.88	0.87	0.91	0.73	0.61	0.95	0.93	0.92	0.93	1.19	1.05	1.06	1.14	1.17	1.16
Standard Deviation	1.21	1.23	1.19	1.18	1.06	0.90	1.19	1.19	1.15	1.25	1.43	1.39	1.47	1.51	1.37	1.36
Share of missing observations	52.71	58.14	48.84	35.66	35.66	17.05	48.84	49.61	47.29	49.61	53.49	48.06	51.94	67.44	100.00	100.00
**Electric power consumption (kWh per capita)**																
Mean	999	1,017	994	1,050	990	955	1,095	1,105	1,108	1,133	1,384	1,351	1,357	1,414	1,467	1,453
Standard Deviation	1,345	1,324	1,335	1,333	1,252	1,279	1,252	1,230	1,239	1,276	1,366	1,361	1,375	1,394	1,369	1,314
Share of missing observations	32.56	32.56	32.56	32.56	32.56	32.56	32.56	32.56	32.56	32.56	100.00	100.00	100.00	100.00	100.00	100.00
**Proportion of seats held by women in national parliaments (%)**																
Mean	13.70	14.13	14.83	15.31	15.32	14.80	16.47	16.71	17.56	18.30	19.76	20.29	20.52	21.17	21.65	22.76
Standard Deviation	9.29	9.57	9.83	10.93	10.45	10.70	10.61	10.80	11.37	12.20	12.16	12.36	11.98	12.35	12.33	10.35
Share of missing observations	4.65	2.33	3.10	3.88	3.88	3.88	3.10	2.33	6.20	3.88	2.33	0.78	1.55	1.55	0.78	100.00
**Total fertility (live births per woman)**																
Mean	4.17	4.12	4.13	3.96	3.92	4.10	3.88	3.84	3.69	3.67	3.28	3.24	3.20	3.16	3.11	3.07
Standard Deviation	1.56	1.51	1.56	1.48	1.44	1.51	1.47	1.42	1.32	1.35	1.35	1.32	1.30	1.27	1.24	1.22
Share of missing observations	0.00	0.00	0.00	0.00	0.00	0.00	0.00	0.00	0.00	0.00	0.00	0.00	0.00	0.00	0.00	0.00
**Immunization, DPT (% of children ages 12-23 months)**																
Mean	78.45	79.60	81.53	81.17	83.22	81.69	83.79	83.54	83.11	81.88	84.74	85.05	84.95	84.70	88.24	88.61
Standard Deviation	18.26	18.42	16.14	17.68	15.08	15.60	15.41	16.24	17.81	17.21	16.37	15.87	14.75	15.50	18.41	18.49
Share of missing observations	1.55	0.78	0.78	0.78	0.78	0.78	0.00	0.00	0.00	0.00	0.00	0.00	0.00	0.00	100.00	100.00
**Immunization, measles (% of children ages 12-23 months)**																
Mean	76.74	78.22	80.04	80.28	81.13	80.43	82.67	82.55	83.03	81.56	84.42	84.20	84.26	84.12	87.70	88.04
Standard Deviation	17.85	17.39	16.23	16.62	15.47	14.99	14.37	14.70	15.30	15.43	15.13	15.36	14.67	15.34	17.55	17.49
Share of missing observations	1.55	0.78	0.78	0.78	0.78	0.78	0.00	0.00	0.00	0.00	0.00	0.00	0.00	0.00	100.00	100.00

Source: Authors' elaboration
