# Supplementary material for: Estimated impact of the 2020 economic downturn on under-5 mortality for 129 countries
Source: PLoS One. 2022 Feb 23;17(2):e0263245. doi: 10.1371/journal.pone.0263245 (PMC8865697; doi:10.1371/journal.pone.0263245)
Supplement: S2 Appendix — (ZIP) [file pone.0263245.s002.zip › S2 Appendix.pdf]

S2 Appendix

In order to fill in missing values in the independent control variables of interest, we performed multiple imputation using multivariate normal regression. We selected this approach because all the variables used in the analysis are continuous and because it uses an iterative Markov chain Monte Carlo method to impute missing values.

The method uses data augmentation to simulate unobserved missing values. The data augmentation process consists of two steps. In the first step, missing values in x_i a replaced by draws from the conditional posterior distribution of $x_{i_{(m)}}$ given the observed data and current values of model parameters independently for each i. In a second step, the new values of the parameters are drawn from their conditional posterior distribution given the observed data and the data imputed in the previous step. These processes are repeated for a number of iterations, which is determined by the length of the burn-in period and the number of iterations between imputations. The length of the burn-in period must be large enough in order to ensure that the chain converges to the stationary distribution. The number of iterations between imputations should be large enough to ensure that the random draws are approximately independent.

For this study, we imputed 65 additional data-sets, since the largest share of missing values in a variable was around 55%. The number of iterations used in the burn-in period to reach stationarity was 2500. In order to reduce the correlation between sets of imputed values, 900 iterations of the Markov chain Monte Carlo were performed between imputations. In doing so, we used an informative ridge prior distribution for the Markov chain Monte Carlo procedure; we selected this prior because some countries had few observations. Moreover, Figure A in S2 Appendix demonstrates convergence of the Markov chain Monte Carlo algorithm, and Figure B in S2 Appendix shows that the auto-correlation is faded after 14 lags. In Table A in S2 Appendix a summary of our multiple imputation is presented.

Multiple imputation summary.

Variable	Complete	Incomplete	Imputed	Total
GDP per capita constant 2010$	3625	374	374	3999
Log GDP per capita constant 2010$	3748	251	251	3999
Physicians (per 1,000 people)	1853	2146	2146	3999
Electric power consumption (kWh per capita)	2132	1867	1867	3999
Proportion of seats held by women in national parliaments (%)	2738	1261	1261	3999
Immunization, DPT (% of children ages 12-23 months)	3639	360	360	3999

Source: Authors' elaboration

The auto-correlation disappears after 25 lags, which ensures that there is independence between imputations.

Convergence of the Markov chain Monte Carlo (MCMC).

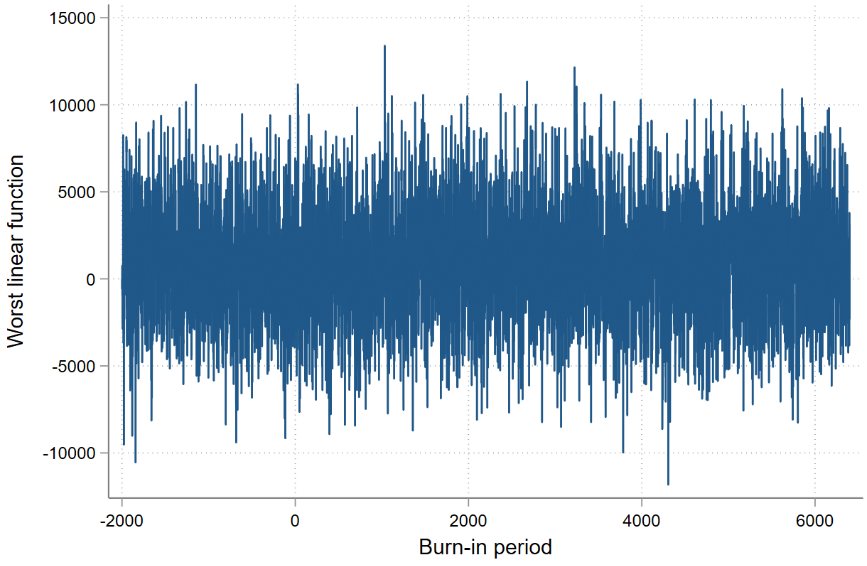

Source: Authors' elaboration

Autocorrelation of the Worst linear function.

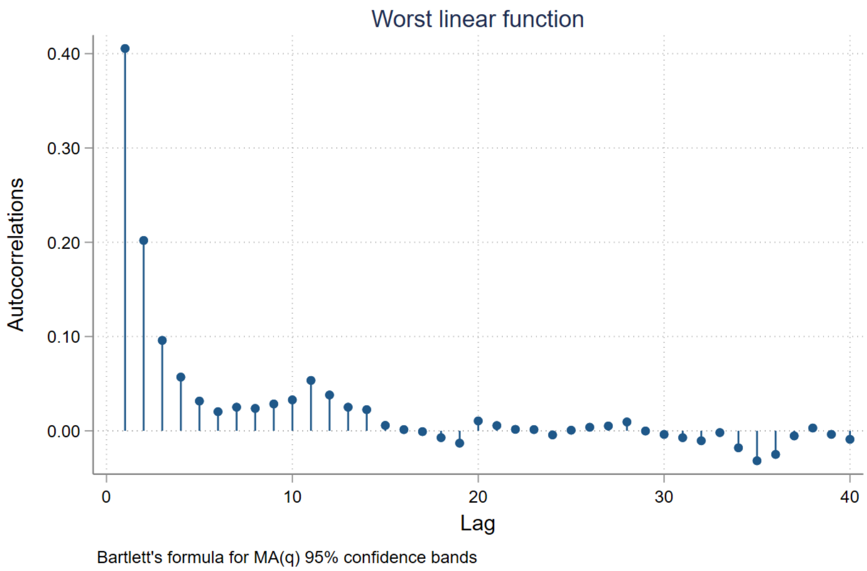

Source: Authors' elaboration
