# Supplementary material for: Estimated impact of the 2020 economic downturn on under-5 mortality for 129 countries
Source: PLoS One. 2022 Feb 23;17(2):e0263245. doi: 10.1371/journal.pone.0263245 (PMC8865697; doi:10.1371/journal.pone.0263245)
Supplement: S3 Appendix — (ZIP) [file pone.0263245.s003.zip › S3 Appendix.pdf]

S3 Appendix

Estimated effect of GDP reductions on U5MR at country level (fitted values).

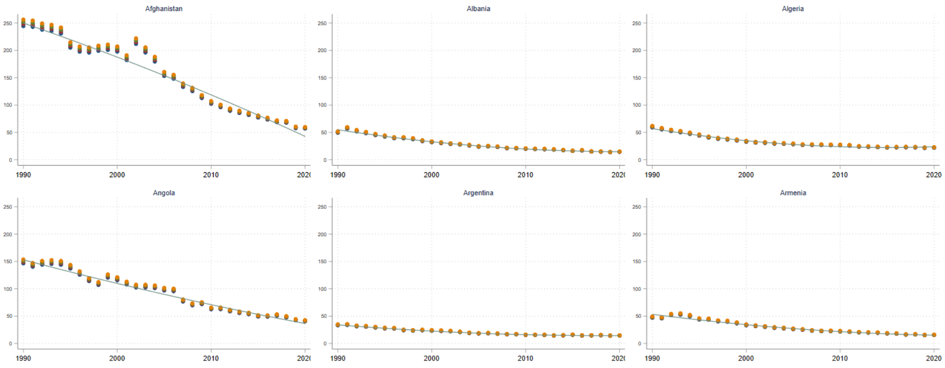

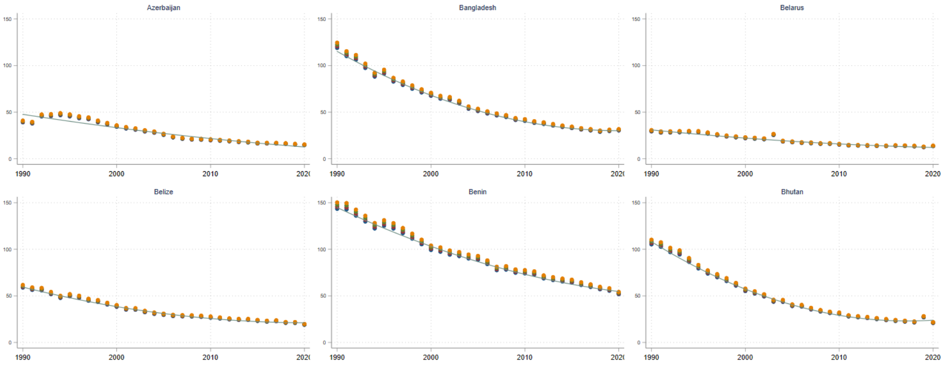

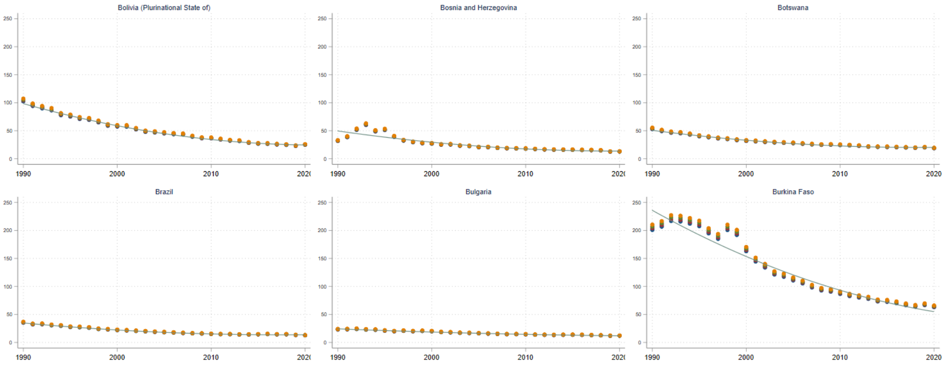

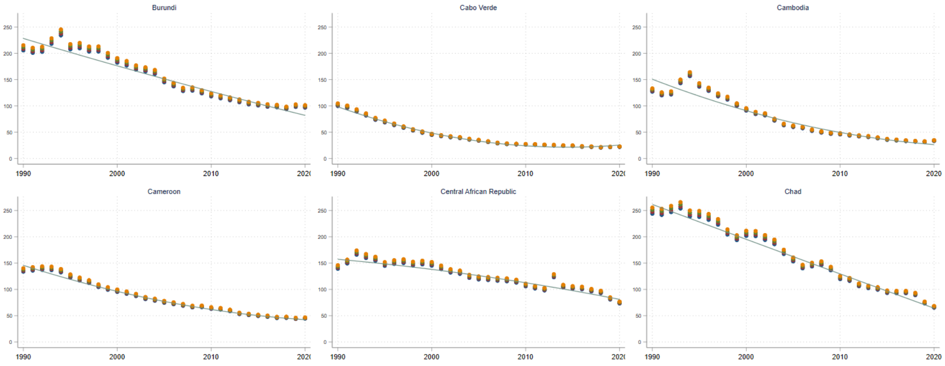

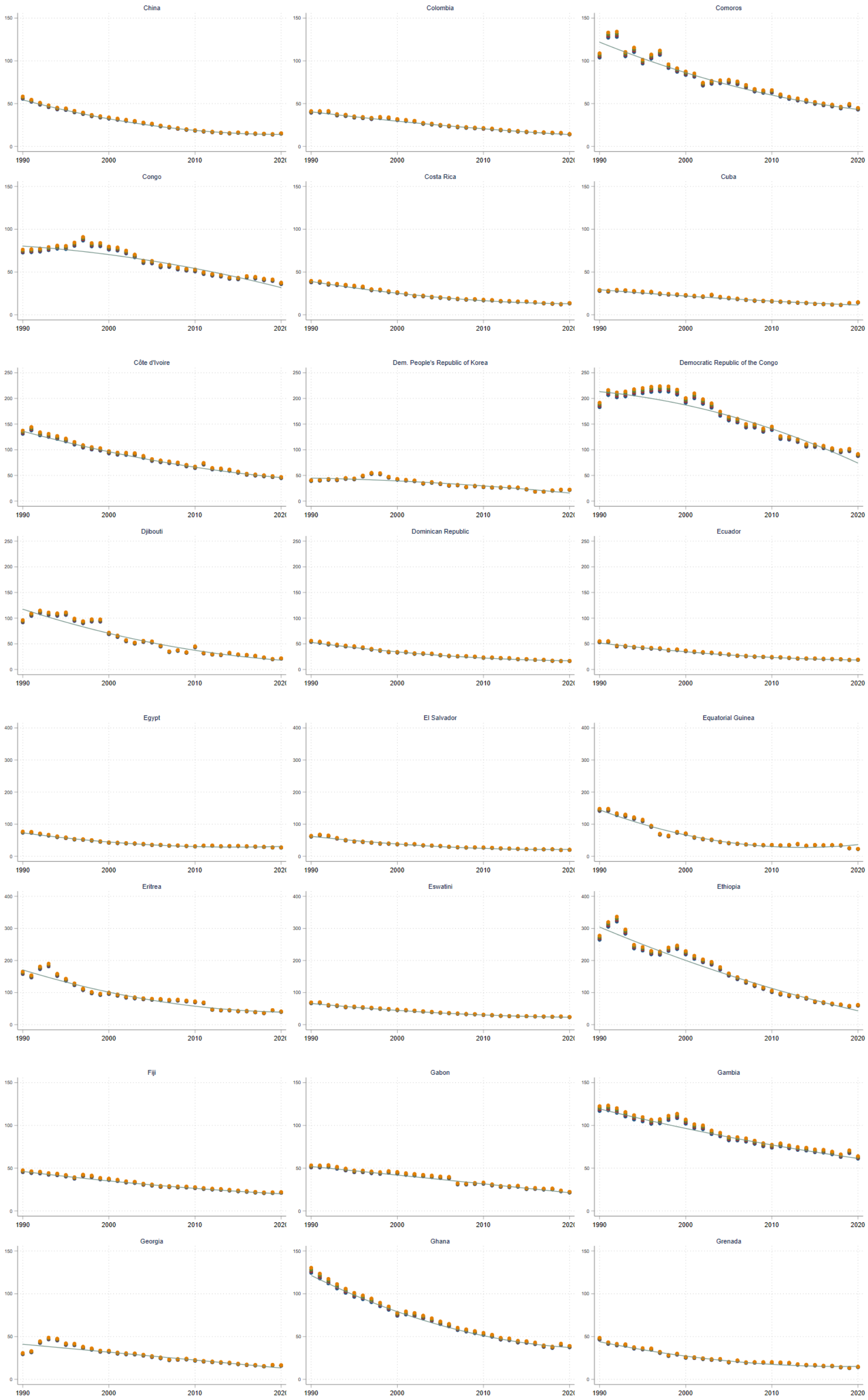
China
Colombia
Comoros
Congo
Costa Rica
Cuba
Côte d'Ivoire
Dem. People's Republic of Korea
Democratic Republic of the Congo
Djibouti
Dominican Republic
Ecuador
Egypt
El Salvador
Equatorial Guinea
Eritrea
Eswatini
Ethiopia
Fiji
Gabon
Gambia
Georgia
Ghana
Grenada

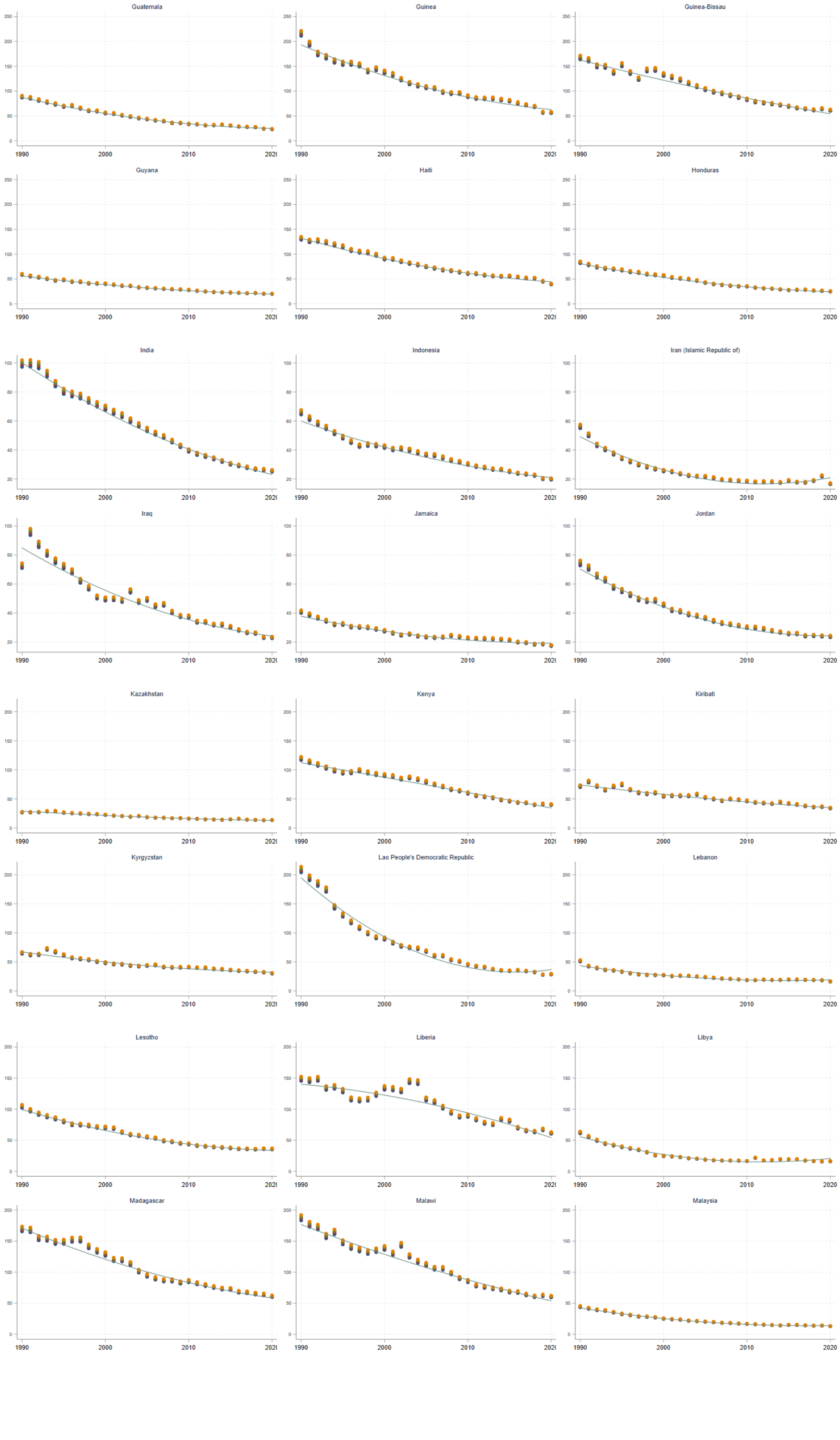
Guatemala
Guinea
Guinea-Bissau
Guyana
Haiti
Honduras
India
Indonesia
Iran (Islamic Republic of)
Iraq
Jamaica
Jordan
Kazakhstan
Kenya
Kiribati
Kyrgyzstan
Lao People's Democratic Republic
Lebanon
Lesotho
Liberia
Libya
Madagascar
Malawi
Malaysia

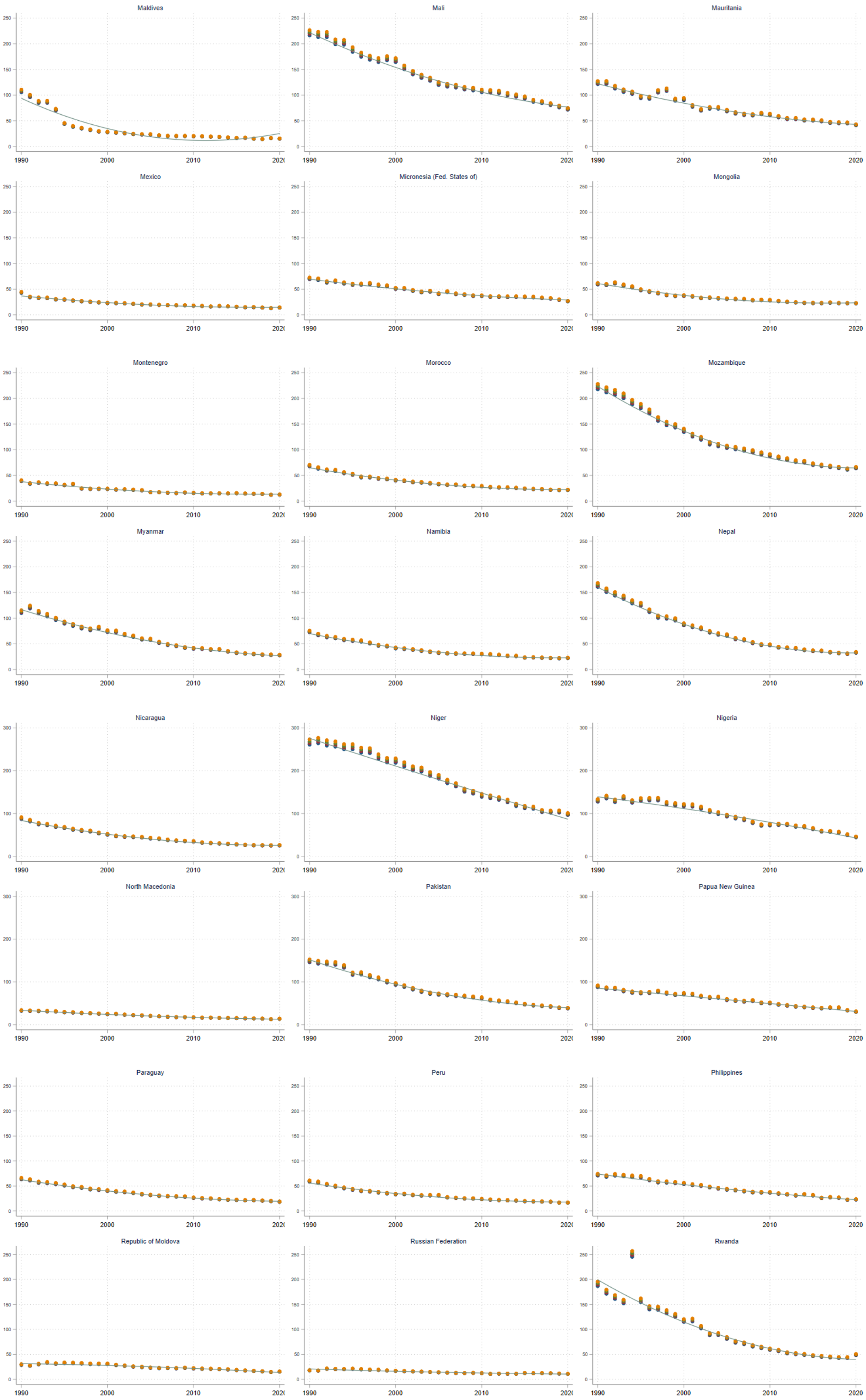
Maldives
Mali
Mauritania
Mexico
Micronesia (Fed. States of)
Mongolia
Montenegro
Morocco
Mozambique
Myanmar
Namibia
Nepal
Nicaragua
Niger
Nigeria
North Macedonia
Pakistan
Papua New Guinea
Paraguay
Peru
Philippines
Republic of Moldova
Russian Federation
Rwanda

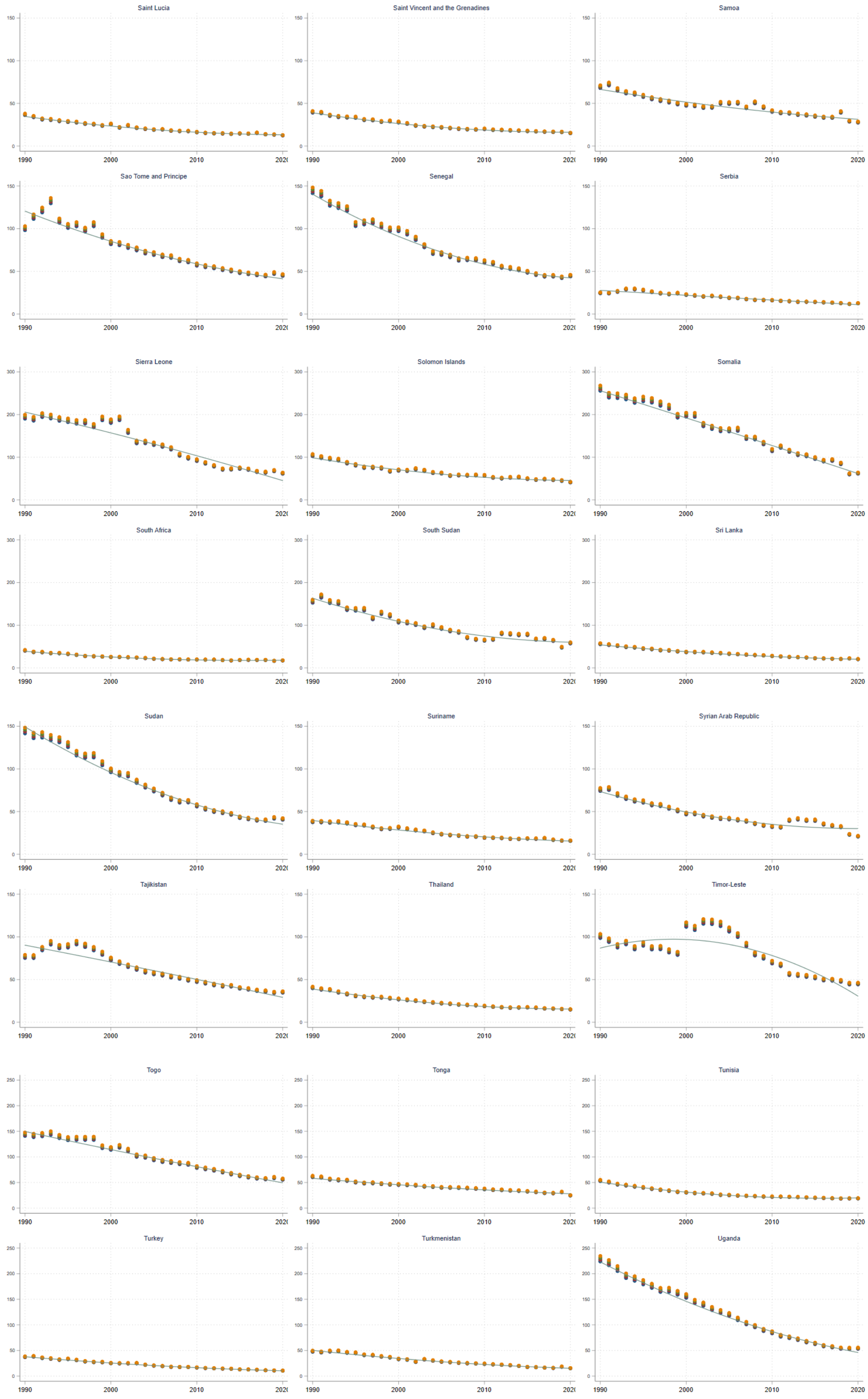
Saint Lucia
Saint Vincent and the Grenadines
Samoa
Sao Tome and Principe
Senegal
Serbia
Sierra Leone
Solomon Islands
Somalia
South Africa
South Sudan
Sri Lanka
Sudan
Suriname
Syrian Arab Republic
Tajikistan
Thailand
Timor-Leste
Togo
Tonga
Tunisia
Turkey
Turkmenistan
Uganda
1990
2000
2010
2020

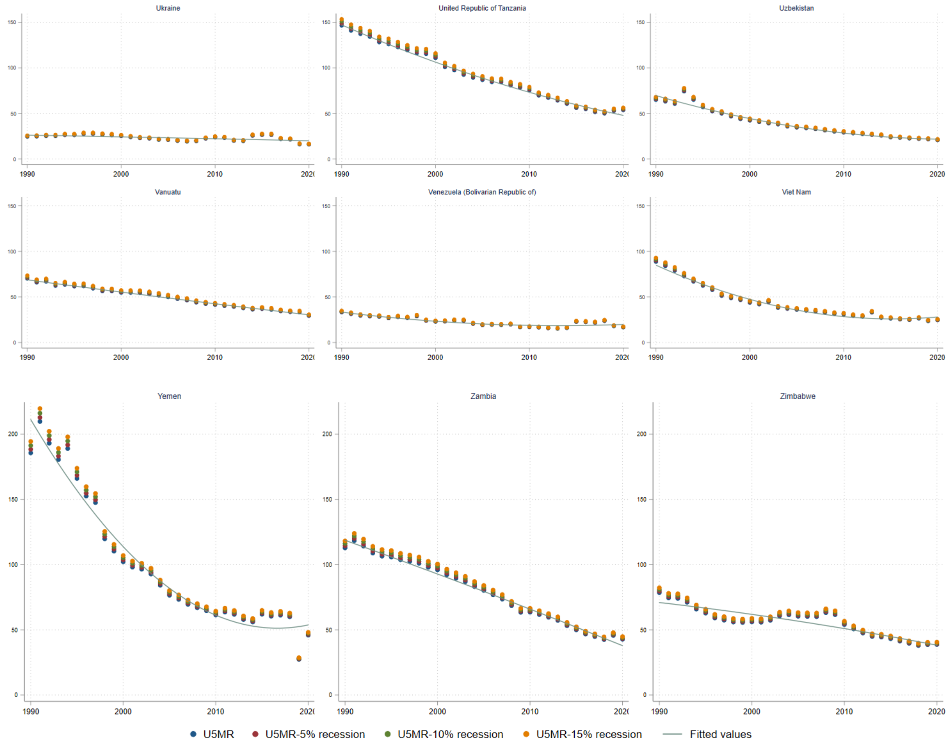

Source: Authors' elaboration
