# Supplementary material for: Estimated impact of the 2020 economic downturn on under-5 mortality for 129 countries
Source: PLoS One. 2022 Feb 23;17(2):e0263245. doi: 10.1371/journal.pone.0263245 (PMC8865697; doi:10.1371/journal.pone.0263245)
Supplement: S4 Appendix — (ZIP) [file pone.0263245.s004.zip › S4 Appendix.pdf]

S4 Appendix

Estimated effect of GDP reduction on U5M (model without controls)

Country	Under-5 deaths	95% CI-lower bound	95% CI-upper bound	U5 deaths 5% reduction on GDP	95% CI-lower bound	95% CI-upper bound	Incremental Deaths at 5% Downturn	U5 deaths 10% reduction on GDP	95% CI-lower bound	95% CI-upper bound	Incremental Deaths at 10% Downturn	U5 deaths 15% reduction on GDP	95% CI-lower bound	95% CI-upper bound	Incremental Deaths at 15% Downturn
Afghanistan	279,869	113,507	690,059	286,568	116,685	703,787	6,699	293,803	120,129	718,566	13,935	301,651	123,878	734,543	21,782
Albania	3,055	1,037	8,998	3,128	1,067	9,172	73	3,207	1,099	9,360	152	3,293	1,134	9,562	238
Algeria	93,430	31,775	274,723	95,667	32,682	280,038	2,236	98,082	33,665	285,756	4,652	100,702	34,737	291,931	7,272
Angola	125,643	44,014	358,663	128,650	45,267	365,628	3,008	131,899	46,626	373,120	6,256	135,422	48,107	381,212	9,779
Argentina	48,814	15,518	153,549	49,982	15,963	156,500	1,168	51,244	16,446	159,672	2,430	52,613	16,972	163,098	3,799
Armenia	4,020	1,382	11,693	4,116	1,421	11,920	96	4,220	1,464	12,164	200	4,332	1,510	12,427	313
Azerbaijan	13,915	4,647	41,661	14,248	4,780	42,465	333	14,607	4,924	43,330	693	14,998	5,081	44,265	1,083
Bangladesh	515,704	198,110	1,342,442	528,048	203,698	1,368,869	12,345	541,381	209,755	1,397,309	25,677	555,841	216,352	1,428,043	40,137
Belarus	8,845	2,930	26,706	9,057	3,013	27,221	212	9,286	3,104	27,775	440	9,534	3,203	28,374	688
Belize	774	266	2,251	793	274	2,295	19	813	282	2,342	39	834	291	2,393	60
Benin	67,402	25,807	176,040	69,015	26,535	179,504	1,613	70,758	27,324	183,231	3,356	72,648	28,184	187,259	5,246
Bhutan	1,470	521	4,149	1,505	535	4,230	35	1,543	551	4,316	73	1,584	569	4,410	114
Bolivia (Plurinational State of)	29,709	10,691	82,555	30,420	10,995	84,164	711	31,188	11,324	85,895	1,479	32,021	11,683	87,765	2,312
Bosnia and Herzegovina	2,259	755	6,757	2,313	777	6,887	54	2,372	800	7,028	112	2,435	826	7,179	176
Botswana	3,998	1,301	12,290	4,094	1,338	12,526	96	4,197	1,379	12,781	199	4,310	1,423	13,056	311
Brazil	181,631	57,287	575,867	185,979	58,931	586,924	4,348	190,674	60,715	598,814	9,043	195,767	62,658	611,651	14,136
Bulgaria	4,484	1,452	13,851	4,591	1,493	14,118	107	4,707	1,538	14,404	223	4,833	1,587	14,714	349
Burkina Faso	149,031	59,030	376,251	152,598	60,689	383,700	3,567	156,451	62,486	391,718	7,420	160,630	64,443	400,384	11,599
Burundi	158,638	69,135	364,015	162,435	71,045	371,390	3,797	166,537	73,113	379,335	7,899	170,985	75,364	387,929	12,347
Cabo Verde	1,091	380	3,133	1,117	391	3,193	26	1,145	402	3,259	54	1,176	415	3,329	85
Cambodia	63,848	24,515	166,288	65,377	25,207	169,561	1,528	67,027	25,957	173,084	3,179	68,818	26,773	176,891	4,969
Cameroon	130,780	49,140	348,060	133,911	50,529	354,886	3,131	137,292	52,036	362,232	6,511	140,959	53,677	370,169	10,179
Central African Republic	44,619	18,710	106,402	45,687	19,231	108,535	1,068	46,840	19,796	110,832	2,222	48,091	20,410	113,316	3,473
Chad	120,242	47,260	305,927	123,120	48,589	311,974	2,878	126,229	50,030	318,483	5,987	129,600	51,598	325,519	9,358
China	1,275,963	417,772	3,897,060	1,306,506	429,731	3,972,155	30,543	1,339,493	442,703	4,052,918	63,530	1,375,271	456,838	4,140,128	99,308
Colombia	55,252	18,018	169,430	56,575	18,534	172,694	1,323	58,003	19,093	176,204	2,751	59,552	19,703	179,994	4,300
Comoros	4,052	1,531	10,723	4,149	1,575	10,934	97	4,254	1,622	11,160	202	4,368	1,673	11,405	315
Congo	19,549	6,967	54,850	20,017	7,165	55,918	468	20,522	7,380	57,066	973	21,070	7,614	58,306	1,521
Costa Rica	4,653	1,486	14,570	4,764	1,528	14,850	111	4,884	1,575	15,151	232	5,015	1,625	15,476	362
Cuba	9,095	3,005	27,532	9,313	3,090	28,063	218	9,548	3,184	28,634	453	9,803	3,285	29,251	708
Côte d'Ivoire	126,899	47,392	339,791	129,937	48,733	346,449	3,038	133,217	50,187	353,613	6,318	136,776	51,771	361,354	9,877
Dem. People's Republic of Korea	41,451	11,376	151,042	42,443	11,688	154,123	992	43,515	12,027	157,440	2,064	44,677	12,396	161,028	3,226

Democratic Republic of the Congo	906,013	376,596	2,179,685	927,701	387,097	2,223,291	21,687	951,123	398,475	2,270,244	45,110	976,528	410,858	2,321,013	70,515
Djibouti	1,882	513	6,908	1,927	527	7,047	45	1,976	542	7,198	94	2,029	559	7,360	146
Dominican Republic	15,264	4,999	46,608	15,629	5,142	47,506	365	16,024	5,297	48,472	760	16,452	5,466	49,515	1,188
Ecuador	29,711	10,029	88,014	30,422	10,316	89,715	711	31,190	10,627	91,545	1,479	32,023	10,965	93,522	2,312
Egypt	299,137	106,426	840,803	306,298	109,452	857,162	7,161	314,031	112,734	874,761	14,894	322,419	116,309	893,773	23,282
El Salvador	12,418	4,345	35,489	12,715	4,469	36,178	297	13,036	4,603	36,919	618	13,385	4,749	37,720	966
Equatorial Guinea	2,489	784	7,900	2,548	806	8,052	60	2,612	831	8,215	124	2,682	857	8,391	194
Eritrea	14,973	4,487	49,971	15,332	4,610	50,987	358	15,719	4,744	52,082	746	16,139	4,890	53,265	1,165
Eswatini	2,676	911	7,862	2,740	937	8,014	64	2,810	965	8,178	133	2,885	996	8,354	208
Ethiopia	848,857	345,676	2,084,485	869,176	355,348	2,125,991	20,319	891,121	365,829	2,170,676	42,264	914,923	377,238	2,218,982	66,066
Fiji	1,683	574	4,935	1,724	591	5,030	40	1,767	608	5,133	84	1,814	628	5,244	131
Gabon	4,373	1,402	13,639	4,478	1,442	13,901	105	4,591	1,486	14,183	218	4,713	1,534	14,488	340
Gambia	17,593	6,970	44,409	18,014	7,166	45,288	421	18,469	7,378	46,235	876	18,963	7,609	47,257	1,369
Georgia	5,092	1,740	14,907	5,214	1,789	15,195	122	5,346	1,843	15,506	254	5,489	1,902	15,841	396
Ghana	123,156	45,670	332,105	126,104	46,963	338,606	2,948	129,287	48,366	345,600	6,132	132,741	49,893	353,156	9,585
Grenada	126	41	392	129	42	399	3	133	43	407	6	136	44	416	10
Guatemala	45,402	15,946	129,272	46,489	16,400	131,783	1,087	47,663	16,892	134,484	2,261	48,936	17,429	137,402	3,534
Guinea	86,397	33,973	219,717	88,465	34,928	224,061	2,068	90,699	35,964	228,736	4,302	93,121	37,091	233,790	6,724
Guinea-Bissau	14,630	5,905	36,245	14,980	6,070	36,966	350	15,358	6,250	37,741	728	15,768	6,445	38,580	1,139
Guyana	1,504	521	4,344	1,540	535	4,428	36	1,579	552	4,519	75	1,621	569	4,617	117
Haiti	55,775	22,200	140,124	57,110	22,824	142,901	1,335	58,552	23,499	145,890	2,777	60,116	24,235	149,121	4,341
Honduras	27,161	9,889	74,599	27,811	10,170	76,055	650	28,514	10,474	77,622	1,352	29,275	10,806	79,314	2,114
India	3,259,649	1,196,249	8,882,194	3,337,676	1,230,164	9,055,765	78,027	3,421,945	1,266,939	9,242,523	162,296	3,513,347	1,306,992	9,444,286	253,698
Indonesia	469,123	161,602	1,361,848	480,353	166,210	1,388,238	11,230	492,481	171,208	1,416,624	23,357	505,635	176,653	1,447,283	36,512
Iran (Islamic Republic of)	121,985	40,322	369,037	124,905	41,476	376,155	2,920	128,059	42,727	383,811	6,074	131,480	44,090	392,078	9,494
Iraq	92,774	31,121	276,570	94,995	32,010	281,913	2,221	97,393	32,974	287,660	4,619	99,995	34,025	293,866	7,221
Jamaica	4,268	1,451	12,556	4,370	1,492	12,798	102	4,481	1,537	13,060	213	4,600	1,586	13,342	332
Jordan	23,325	8,196	66,377	23,883	8,429	67,666	558	24,486	8,683	69,053	1,161	25,140	8,958	70,551	1,815
Kazakhstan	24,274	7,667	76,850	24,855	7,887	78,326	581	25,483	8,126	79,913	1,209	26,163	8,386	81,626	1,889
Kenya	250,460	96,008	653,386	256,455	98,716	666,243	5,995	262,930	101,653	680,080	12,470	269,953	104,850	695,033	19,493
Kiribati	445	165	1,201	455	169	1,225	11	467	174	1,250	22	479	180	1,277	35
Kyrgyzstan	28,103	10,847	72,814	28,776	11,153	74,249	673	29,503	11,484	75,793	1,399	30,291	11,845	77,461	2,187
Lao People's Democratic Republic	23,809	8,848	64,072	24,379	9,098	65,327	570	24,995	9,370	66,676	1,185	25,662	9,666	68,135	1,853
Lebanon	9,257	3,074	27,878	9,478	3,162	28,416	222	9,718	3,257	28,995	461	9,977	3,361	29,620	720
Lesotho	8,308	3,139	21,990	8,507	3,228	22,422	199	8,722	3,324	22,886	414	8,955	3,429	23,388	647
Liberia	37,345	15,202	91,742	38,239	15,627	93,569	894	39,204	16,088	95,535	1,859	40,252	16,590	97,661	2,907
Libya	9,768	3,216	29,666	10,002	3,308	30,238	234	10,254	3,408	30,853	486	10,528	3,517	31,517	760
Madagascar	219,003	89,969	533,099	224,246	92,483	543,734	5,242	229,907	95,207	555,186	10,904	236,048	98,172	567,566	17,045
Malawi	151,606	61,999	370,722	155,235	63,732	378,111	3,629	159,154	65,611	386,066	7,548	163,405	67,656	394,666	11,800
Malaysia	32,324	10,150	102,942	33,098	10,441	104,918	774	33,934	10,757	107,043	1,609	34,840	11,102	109,336	2,516
Maldives	525	171	1,612	537	176	1,643	13	551	181	1,677	26	566	187	1,713	41
Mali	155,656	61,717	392,581	159,382	63,451	400,354	3,726	163,406	65,330	408,722	7,750	167,771	67,375	417,766	12,115
Mauritania	20,460	7,593	55,134	20,950	7,808	56,213	490	21,479	8,041	57,374	1,019	22,053	8,295	58,629	1,592
Mexico	142,829	45,385	449,488	146,248	46,687	458,124	3,419	149,940	48,099	467,411	7,111	153,945	49,638	477,439	11,116

Micronesia (Fed. States of)	294	105	824	301	108	840	7	309	111	858	15	317	115	876	23
Mongolia	7,499	2,587	21,739	7,678	2,660	22,161	180	7,872	2,740	22,614	373	8,083	2,828	23,103	584
Montenegro	543	177	1,670	556	182	1,702	13	570	187	1,736	27	586	193	1,774	42
Morocco	73,082	25,667	208,088	74,831	26,397	212,130	1,749	76,720	27,190	216,478	3,639	78,769	28,053	221,175	5,688
Mozambique	251,430	101,771	621,169	257,449	104,622	633,521	6,019	263,949	107,710	646,819	12,519	270,999	111,073	661,194	19,569
Myanmar	143,552	53,958	381,912	146,988	55,484	389,403	3,436	150,699	57,138	397,463	7,147	154,724	58,939	406,173	11,173
Namibia	5,595	1,864	16,792	5,729	1,917	17,116	134	5,873	1,975	17,464	279	6,030	2,038	17,841	435
Nepal	115,550	45,727	291,991	118,316	47,012	297,771	2,766	121,303	48,404	303,992	5,753	124,543	49,920	310,716	8,993
Nicaragua	18,802	6,933	50,993	19,252	7,129	51,991	450	19,738	7,342	53,064	936	20,266	7,574	54,223	1,463
Niger	243,175	99,106	596,677	248,996	101,878	608,559	5,821	255,283	104,883	621,353	12,108	262,102	108,153	635,183	18,926
Nigeria	856,896	308,792	2,377,887	877,408	317,562	2,424,234	20,512	899,561	327,073	2,474,096	42,665	923,588	337,432	2,527,962	66,692
North Macedonia	1,969	663	5,846	2,016	682	5,959	47	2,067	702	6,081	98	2,122	725	6,212	153
Pakistan	1,000,517	383,952	2,607,185	1,024,467	394,784	2,658,500	23,950	1,050,333	406,526	2,713,725	49,815	1,078,388	419,312	2,773,403	77,870
Papua New Guinea	27,906	10,053	77,463	28,573	10,338	78,973	668	29,295	10,648	80,597	1,389	30,077	10,985	82,351	2,172
Paraguay	12,496	4,218	37,016	12,795	4,339	37,732	299	13,118	4,469	38,501	622	13,468	4,612	39,332	973
Peru	46,124	15,302	139,025	47,228	15,740	141,707	1,104	48,420	16,215	144,592	2,296	49,713	16,732	147,708	3,590
Philippines	242,622	85,832	685,817	248,429	88,274	699,151	5,808	254,702	90,923	713,495	12,080	261,505	93,808	728,990	18,883
Republic of Moldova	4,440	1,559	12,649	4,547	1,603	12,894	106	4,661	1,651	13,159	221	4,786	1,704	13,444	346
Russian Federation	114,014	35,818	362,922	116,744	36,846	369,888	2,729	119,691	37,962	377,378	5,677	122,888	39,177	385,465	8,874
Rwanda	78,646	31,000	199,522	80,528	31,871	203,469	1,883	82,561	32,816	207,716	3,916	84,767	33,844	212,307	6,121
Saint Lucia	150	48	466	154	50	475	4	157	51	485	7	162	53	495	12
Saint Vincent and the Grenadines	122	40	369	125	41	376	3	128	43	384	6	131	44	392	9
Samoa	558	194	1,607	571	199	1,638	13	586	205	1,672	28	601	212	1,708	43
Sao Tome and Principe	1,081	411	2,842	1,107	423	2,898	26	1,135	435	2,958	54	1,165	449	3,023	84
Senegal	83,013	31,185	220,974	85,000	32,067	225,308	1,987	87,146	33,023	229,971	4,133	89,473	34,064	235,010	6,461
Serbia	6,675	2,205	20,203	6,834	2,268	20,593	160	7,007	2,337	21,012	332	7,194	2,411	21,464	519
Sierra Leone	62,849	25,894	152,541	64,353	26,617	155,586	1,504	65,978	27,401	158,865	3,129	67,740	28,254	162,410	4,892
Solomon Islands	3,296	1,240	8,764	3,375	1,275	8,936	79	3,461	1,313	9,121	164	3,553	1,354	9,321	257
Somalia	96,456	30,164	308,437	98,764	30,994	314,724	2,309	101,258	31,892	321,494	4,802	103,963	32,870	328,817	7,507
South Africa	86,983	28,438	266,051	89,065	29,252	271,177	2,082	91,314	30,136	276,690	4,331	93,753	31,098	282,643	6,770
South Sudan	75,191	29,915	188,996	76,991	30,755	192,741	1,800	78,935	31,665	196,772	3,744	81,043	32,656	201,129	5,852
Sri Lanka	34,005	11,786	98,117	34,819	12,122	100,020	814	35,698	12,486	102,066	1,693	36,652	12,883	104,277	2,647
Sudan	183,077	67,615	495,706	187,459	69,530	505,402	4,382	192,192	71,608	515,835	9,115	197,325	73,870	527,107	14,249
Suriname	759	246	2,337	777	253	2,382	18	797	261	2,431	38	818	269	2,483	59

Syrian Arab Republic	36,882	10,790	126,062	37,764	11,092	128,576	883	38,718	11,419	131,282	1,836	39,752	11,775	134,206	2,870
Tajikistan	51,143	19,807	132,056	52,367	20,365	134,659	1,224	53,690	20,970	137,461	2,546	55,124	21,629	140,489	3,980
Thailand	59,407	19,765	178,558	60,829	20,330	182,004	1,422	62,364	20,943	185,711	2,958	64,030	21,611	189,714	4,624
Timor-Leste	7,193	2,819	18,350	7,365	2,899	18,713	172	7,551	2,985	19,103	358	7,753	3,078	19,525	560
Togo	56,223	22,542	140,232	57,569	23,174	143,015	1,346	59,023	23,859	146,011	2,799	60,599	24,605	149,249	4,376
Tonga	245	85	709	251	87	723	6	257	90	738	12	264	92	754	19
Tunisia	19,417	6,657	56,635	19,882	6,847	57,732	465	20,383	7,053	58,912	967	20,928	7,277	60,186	1,511
Turkey	72,774	22,398	236,452	74,516	23,042	240,982	1,742	76,397	23,740	245,853	3,623	78,438	24,501	251,112	5,664
Turkmenistan	10,168	3,337	30,985	10,411	3,432	31,582	243	10,674	3,536	32,225	506	10,959	3,649	32,918	791
Uganda	308,549	120,516	789,954	315,934	123,908	805,551	7,386	323,911	127,585	822,339	15,362	332,563	131,589	840,482	24,014
Ukraine	48,486	17,165	136,963	49,647	17,653	139,626	1,161	50,901	18,183	142,491	2,414	52,260	18,760	145,586	3,774
United Republic of Tanzania	383,628	149,719	982,974	392,811	153,934	1,002,380	9,183	402,728	158,503	1,023,266	19,101	413,486	163,477	1,045,838	29,858
Uzbekistan	89,151	32,296	246,099	91,285	33,212	250,899	2,134	93,590	34,207	256,064	4,439	96,090	35,289	261,643	6,939
Vanuatu	992	353	2,787	1,016	363	2,842	24	1,041	374	2,900	49	1,069	386	2,963	77
Venezuela (Bolivarian Republic of)	39,993	10,758	148,677	40,950	11,057	151,662	957	41,984	11,381	154,876	1,991	43,105	11,734	158,350	3,113
Viet Nam	226,042	83,356	612,969	231,452	85,718	624,956	5,411	237,296	88,279	637,854	11,254	243,634	91,069	651,789	17,593
Yemen	186,570	74,594	466,635	191,036	76,687	475,890	4,466	195,859	78,956	485,854	9,289	201,091	81,425	496,623	14,521
Zambia	88,936	33,110	238,893	91,065	34,047	243,572	2,129	93,365	35,063	248,606	4,428	95,858	36,170	254,045	6,922
Zimbabwe	72,349	27,585	189,754	74,081	28,364	193,484	1,732	75,951	29,208	197,499	3,602	77,980	30,127	201,837	5,631
**Total**	**16,829,307**			**17,232,154**			**402,847**	**17,667,229**			**837,922**	**18,139,129**			**1,309,822**

Source: Authors' elaboration
