# Supplementary material for: Estimated impact of the 2020 economic downturn on under-5 mortality for 129 countries
Source: PLoS One. 2022 Feb 23;17(2):e0263245. doi: 10.1371/journal.pone.0263245 (PMC8865697; doi:10.1371/journal.pone.0263245)
Supplement: S5 Appendix — (ZIP) [file pone.0263245.s005.zip › S5 Appendix.pdf]

S5 Appendix

Estimated effect of GDP reduction on U5M (model with controls)

Country	Under-5 deaths	95% Confidence Interval - lower bound	95% Confidence Interval - upper bound	Under-5 deaths 5% reduction on GDP	95% Confidence Interval - lower bound	95% Confidence Interval - upper bound	Incremental Deaths at 5% Downturn	Under-5 deaths 10% reduction on GDP	95% Confidence Interval - lower bound	95% Confidence Interval - upper bound	Incremental Deaths at 10% Downturn	Under-5 deaths 15% reduction on GDP	95% Confidence Interval - lower bound	95% Confidence Interval - upper bound	Incremental Deaths at 15% Downturn
Afghanistan	323,613	120,308	870,476	328,370	122,533	879,983	4,757	333,460	124,920	890,133	9,848	338,927	127,492	901,013	15,315
Albania	2,394	752	7,622	2,429	766	7,701	35	2,466	781	7,785	73	2,507	798	7,875	113
Algeria	110,808	34,603	354,838	112,437	35,262	358,513	1,629	114,180	35,971	362,432	3,372	116,052	36,735	366,626	5,244
Angola	235,624	75,729	733,123	239,088	77,167	740,769	3,464	242,794	78,712	748,924	7,170	246,775	80,377	757,656	11,151
Argentina	53,650	15,722	183,073	54,438	16,025	184,938	789	55,282	16,349	186,926	1,633	56,189	16,700	189,054	2,539
Armenia	3,166	1,000	10,018	3,212	1,019	10,123	47	3,262	1,040	10,234	96	3,316	1,062	10,353	150
Azerbaijan	12,099	3,740	39,138	12,277	3,812	39,542	178	12,467	3,888	39,971	368	12,671	3,971	40,431	573
Bangladesh	435,117	153,268	1,235,269	441,513	156,135	1,248,500	6,396	448,358	159,212	1,262,622	13,241	455,709	162,528	1,277,750	20,592
Belarus	7,379	2,244	24,266	7,487	2,287	24,517	108	7,603	2,333	24,784	225	7,728	2,382	25,070	349
Belize	749	237	2,367	760	241	2,392	11	772	246	2,418	23	784	252	2,446	35
Benin	98,813	34,464	283,314	100,266	35,109	286,346	1,453	101,820	35,801	289,583	3,007	103,489	36,547	293,050	4,676
Bhutan	1,332	435	4,078	1,352	444	4,120	20	1,373	453	4,166	41	1,395	462	4,214	63
Bolivia (Plurinational State of)	29,494	9,774	88,998	29,928	9,959	89,932	434	30,391	10,158	90,929	898	30,890	10,372	91,996	1,396
Bosnia and Herzegovina	1,702	521	5,557	1,727	531	5,614	25	1,753	542	5,676	52	1,782	553	5,741	81
Botswana	5,091	1,534	16,899	5,166	1,563	17,072	75	5,246	1,595	17,257	155	5,332	1,629	17,455	241
Brazil	182,846	53,523	624,640	185,534	54,554	630,985	2,688	188,410	55,662	637,751	5,564	191,499	56,857	644,989	8,653
Bulgaria	3,723	1,113	12,449	3,778	1,135	12,577	55	3,836	1,158	12,713	113	3,899	1,182	12,858	176
Burkina Faso	218,887	79,526	602,461	222,105	81,005	608,984	3,218	225,548	82,591	615,948	6,661	229,246	84,300	623,410	10,359
Burundi	198,778	78,436	503,757	201,700	79,854	509,463	2,922	204,827	81,376	515,559	6,049	208,185	83,013	522,098	9,407
Cabo Verde	1,146	367	3,582	1,163	374	3,620	17	1,181	381	3,660	35	1,200	389	3,702	54
Cambodia	59,438	20,803	169,830	60,312	21,191	171,659	874	61,247	21,607	173,611	1,809	62,251	22,056	175,702	2,813
Cameroon	183,530	63,386	531,403	186,228	64,575	537,061	2,698	189,115	65,852	543,100	5,585	192,215	67,229	549,568	8,685
Central African Republic	54,126	20,663	141,779	54,922	21,043	143,347	796	55,773	21,449	145,023	1,647	56,688	21,887	146,819	2,561
Chad	191,519	68,658	534,235	194,334	69,935	540,011	2,815	197,347	71,306	546,177	5,828	200,582	72,783	552,783	9,064
China	1,235,908	372,924	4,095,918	1,254,076	380,064	4,138,001	18,169	1,273,517	387,734	4,182,878	37,609	1,294,396	396,005	4,230,905	58,489
Colombia	51,670	15,615	170,968	52,429	15,915	172,715	760	53,242	16,237	174,578	1,572	54,115	16,585	176,571	2,445
Comoros	5,310	1,842	15,306	5,388	1,877	15,468	78	5,471	1,914	15,642	162	5,561	1,954	15,827	251
Congo	29,476	9,685	89,706	29,909	9,869	90,644	433	30,373	10,066	91,644	897	30,871	10,279	92,715	1,395
Costa Rica	4,588	1,351	15,584	4,656	1,377	15,743	67	4,728	1,405	15,912	140	4,806	1,435	16,093	217
Cuba	8,100	2,462	26,655	8,219	2,509	26,929	119	8,347	2,559	27,222	246	8,484	2,614	27,535	383
Côte d'Ivoire	185,002	63,376	540,040	187,721	64,569	545,761	2,720	190,631	65,850	551,866	5,630	193,757	67,230	558,404	8,755
Dem. People's Republic of Korea	37,542	10,563	133,430	38,094	10,757	134,899	552	38,684	10,966	136,467	1,142	39,318	11,191	138,146	1,777

Democratic Republic of the Congo	1,388,004	524,706	3,671,682	1,408,409	534,338	3,712,285	20,405	1,430,241	544,670	3,755,652	42,237	1,453,691	555,796	3,802,143	65,687
Djibouti	2,076	583	7,397	2,107	593	7,478	31	2,139	605	7,563	63	2,174	618	7,655	98
Dominican Republic	16,310	4,937	53,880	16,549	5,032	54,433	240	16,806	5,133	55,022	496	17,081	5,243	55,653	772
Ecuador	30,859	9,623	98,961	31,312	9,806	99,980	454	31,798	10,004	101,068	939	32,319	10,217	102,231	1,460
Egypt	339,094	110,682	1,038,877	344,079	112,777	1,049,774	4,985	349,413	115,027	1,061,399	10,319	355,141	117,452	1,073,846	16,047
El Salvador	11,381	3,664	35,348	11,548	3,734	35,718	167	11,727	3,808	36,112	346	11,919	3,889	36,534	539
Equatorial Guinea	4,411	1,284	15,155	4,476	1,309	15,309	65	4,546	1,335	15,473	134	4,620	1,364	15,649	209
Eritrea	19,506	6,022	63,177	19,793	6,133	63,871	287	20,099	6,253	64,611	594	20,429	6,381	65,405	923
Eswatini	3,328	1,046	10,595	3,377	1,066	10,705	49	3,430	1,087	10,821	101	3,486	1,110	10,946	158
Ethiopia	992,985	369,329	2,669,755	1,007,582	376,148	2,698,999	14,598	1,023,202	383,463	2,730,228	30,217	1,039,977	391,343	2,763,698	46,993
Fiji	1,887	593	6,005	1,915	604	6,067	28	1,944	616	6,133	57	1,976	629	6,204	89
Gabon	6,873	2,046	23,087	6,974	2,085	23,322	101	7,082	2,128	23,573	209	7,198	2,173	23,841	325
Gambia	25,061	9,085	69,132	25,430	9,254	69,879	368	25,824	9,436	70,676	763	26,247	9,631	71,531	1,186
Georgia	4,272	1,350	13,518	4,334	1,376	13,657	63	4,402	1,403	13,806	130	4,474	1,433	13,966	202
Ghana	156,000	53,051	458,733	158,294	54,049	463,595	2,293	160,748	55,121	468,784	4,747	163,383	56,276	474,341	7,383
Grenada	128	38	430	130	39	435	2	132	40	439	4	134	41	444	6
Guatemala	47,406	15,369	146,228	48,103	15,661	147,751	697	48,848	15,974	149,376	1,443	49,649	16,312	151,115	2,243
Guinea	117,136	42,447	323,242	118,858	43,239	326,721	1,722	120,700	44,089	330,436	3,564	122,679	45,004	334,415	5,543
Guinea-Bissau	18,386	6,779	49,865	18,656	6,905	50,409	270	18,946	7,039	50,989	559	19,256	7,184	51,612	870
Guyana	1,467	469	4,590	1,488	478	4,638	22	1,511	487	4,689	45	1,536	497	4,743	69
Haiti	49,167	17,882	135,189	49,890	18,213	136,658	723	50,663	18,569	138,226	1,496	51,494	18,953	139,907	2,327
Honduras	24,945	8,339	74,620	25,312	8,497	75,405	367	25,705	8,666	76,244	759	26,126	8,848	77,141	1,181
India	2,929,298	986,082	8,701,895	2,972,361	1,004,659	8,793,951	43,063	3,018,437	1,024,604	8,892,182	89,139	3,067,926	1,046,101	8,997,378	138,628
Indonesia	461,840	147,090	1,450,106	468,629	149,891	1,465,156	6,789	475,893	152,899	1,481,208	14,054	483,696	156,142	1,498,392	21,856
Iran (Islamic Republic of)	125,719	38,512	410,401	127,567	39,248	414,631	1,848	129,544	40,038	419,143	3,826	131,668	40,891	423,973	5,950
Iraq	121,740	37,598	394,186	123,529	38,317	398,246	1,790	125,444	39,089	402,575	3,705	127,501	39,922	407,209	5,761
Jamaica	3,971	1,238	12,740	4,030	1,262	12,872	58	4,092	1,287	13,013	121	4,159	1,314	13,163	188
Jordan	24,669	7,962	76,434	25,031	8,113	77,233	363	25,419	8,275	78,086	751	25,836	8,449	78,999	1,167
Kazakhstan	25,519	7,467	87,216	25,894	7,610	88,105	375	26,295	7,764	89,052	777	26,726	7,931	90,066	1,208
Kenya	277,566	97,930	786,710	281,646	99,765	795,117	4,080	286,012	101,734	804,090	8,446	290,701	103,855	813,702	13,136
Kiribati	508	173	1,494	515	176	1,510	7	523	179	1,527	15	532	183	1,545	24
Kyrgyzstan	22,676	8,028	64,047	23,009	8,178	64,736	333	23,366	8,339	65,473	690	23,749	8,512	66,261	1,073
Lao People's Democratic Republic	22,522	7,702	65,854	22,853	7,847	66,552	331	23,207	8,003	67,298	685	23,587	8,170	68,096	1,066
Lebanon	9,088	2,782	29,693	9,222	2,835	29,999	134	9,365	2,892	30,326	277	9,518	2,953	30,676	430
Lesotho	8,928	3,091	25,792	9,060	3,149	26,066	131	9,200	3,211	26,359	272	9,351	3,278	26,672	423
Liberia	44,683	16,597	120,296	45,340	16,903	121,616	657	46,042	17,232	123,025	1,360	46,797	17,585	124,536	2,115
Libya	9,972	3,033	32,782	10,119	3,091	33,120	147	10,276	3,154	33,480	303	10,444	3,221	33,865	472
Madagascar	245,912	91,406	661,589	249,528	93,087	668,881	3,615	253,396	94,890	676,669	7,483	257,550	96,833	685,016	11,638
Malawi	173,658	64,879	464,823	176,211	66,074	469,933	2,553	178,942	67,356	475,391	5,284	181,876	68,737	481,241	8,218
Malaysia	33,845	9,852	116,269	34,343	10,042	117,453	498	34,875	10,245	118,715	1,030	35,447	10,465	120,066	1,602
Maldives	529	159	1,760	537	162	1,778	8	545	166	1,797	16	554	169	1,817	25
Mali	257,889	93,417	711,934	261,680	95,149	719,675	3,791	265,737	97,008	727,938	7,848	270,094	99,011	736,794	12,205
Mauritania	28,358	9,644	83,384	28,774	9,825	84,268	417	29,221	10,020	85,211	863	29,700	10,230	86,221	1,342
Mexico	151,002	44,437	513,117	153,221	45,292	518,343	2,220	155,597	46,210	523,914	4,595	158,148	47,201	529,875	7,146

Micronesia (Fed. States of)	320	105	973	325	107	983	5	330	109	994	10	335	112	1,006	15
Mongolia	8,259	2,632	25,918	8,381	2,682	26,187	121	8,511	2,736	26,474	251	8,650	2,794	26,781	391
Montenegro	457	137	1,529	464	139	1,545	7	471	142	1,562	14	479	145	1,580	22
Morocco	71,179	22,985	220,422	72,225	23,421	222,725	1,046	73,345	23,890	225,181	2,166	74,547	24,395	227,811	3,369
Mozambique	328,481	121,560	887,628	333,310	123,805	897,341	4,829	338,476	126,214	907,714	9,996	344,026	128,809	918,831	15,545
Myanmar	123,623	42,782	357,219	125,441	43,585	361,022	1,817	127,385	44,447	365,082	3,762	129,474	45,377	369,430	5,850
Namibia	7,403	2,282	24,016	7,512	2,326	24,262	109	7,628	2,373	24,525	225	7,753	2,423	24,806	350
Nepal	88,023	32,010	242,046	89,317	32,605	244,667	1,294	90,701	33,244	247,465	2,679	92,188	33,932	250,463	4,166
Nicaragua	16,495	5,553	48,995	16,738	5,658	49,515	242	16,997	5,770	50,069	502	17,276	5,891	50,663	781
Niger	461,338	171,118	1,243,775	468,120	174,273	1,257,434	6,782	475,377	177,657	1,272,020	14,039	483,171	181,302	1,287,654	21,833
Nigeria	1,503,219	497,646	4,540,714	1,525,317	507,077	4,588,238	22,098	1,548,962	517,205	4,638,937	45,743	1,574,358	528,124	4,693,221	71,139
North Macedonia	1,505	469	4,825	1,527	478	4,875	22	1,551	488	4,928	46	1,576	498	4,985	71
Pakistan	1,054,683	371,239	2,996,334	1,070,187	378,185	3,028,413	15,505	1,086,777	385,641	3,062,651	32,094	1,104,595	393,676	3,099,329	49,912
Papua New Guinea	32,868	10,947	98,689	33,351	11,154	99,722	483	33,868	11,377	100,823	1,000	34,424	11,617	102,002	1,555
Paraguay	12,704	3,950	40,860	12,891	4,025	41,283	187	13,091	4,106	41,733	387	13,305	4,193	42,216	601
Peru	46,296	14,179	151,157	46,976	14,451	152,711	681	47,704	14,742	154,368	1,409	48,486	15,056	156,142	2,191
Philippines	240,542	78,574	736,383	244,078	80,067	744,053	3,536	247,862	81,671	752,234	7,320	251,925	83,400	760,993	11,384
Republic of Moldova	3,073	998	9,467	3,118	1,016	9,565	45	3,166	1,037	9,671	94	3,218	1,059	9,783	145
Russian Federation	100,190	29,103	344,916	101,663	29,663	348,426	1,473	103,239	30,265	352,168	3,049	104,932	30,914	356,172	4,741
Rwanda	91,223	33,071	251,630	92,564	33,686	254,348	1,341	93,999	34,347	257,250	2,776	95,540	35,059	260,360	4,317
Saint Lucia	134	40	452	136	40	456	2	138	41	461	4	140	42	466	6
Saint Vincent and the Grenadines	115	35	378	117	36	382	2	119	37	386	4	121	37	390	5
Samoa	740	236	2,322	751	241	2,346	11	763	245	2,371	23	776	251	2,399	35
Sao Tome and Principe	1,419	495	4,064	1,440	505	4,107	21	1,462	515	4,154	43	1,486	525	4,203	67
Senegal	114,939	39,655	333,149	116,629	40,400	336,690	1,690	118,437	41,200	340,469	3,498	120,379	42,062	344,516	5,439
Serbia	5,194	1,579	17,091	5,271	1,609	17,269	76	5,352	1,641	17,458	158	5,440	1,676	17,660	246
Sierra Leone	71,002	26,707	188,764	72,046	27,198	190,842	1,044	73,163	27,726	193,062	2,161	74,362	28,294	195,442	3,360
Solomon Islands	4,201	1,453	12,150	4,263	1,480	12,279	62	4,329	1,509	12,416	128	4,400	1,541	12,563	199
Somalia	173,482	53,816	559,233	176,032	54,802	565,442	2,550	178,761	55,859	572,072	5,279	181,691	56,998	579,178	8,210
South Africa	99,249	29,951	328,878	100,708	30,525	332,249	1,459	102,269	31,142	335,844	3,020	103,946	31,807	339,691	4,697
South Sudan	97,763	35,661	268,011	99,200	36,325	270,904	1,437	100,737	37,038	273,993	2,975	102,389	37,805	277,302	4,627
Sri Lanka	33,501	10,656	105,320	33,994	10,859	106,418	492	34,521	11,076	107,589	1,019	35,087	11,311	108,842	1,585
Sudan	256,024	86,492	757,851	259,787	88,122	765,863	3,764	263,814	89,872	774,412	7,791	268,140	91,758	783,567	12,116
Suriname	809	243	2,697	821	247	2,725	12	834	252	2,754	25	847	258	2,786	38

Syrian Arab Republic	39,592	11,602	135,111	40,174	11,820	136,548	582	40,797	12,054	138,082	1,205	41,466	12,306	139,724	1,874
Tajikistan	46,998	16,668	132,524	47,689	16,979	133,949	691	48,429	17,312	135,471	1,430	49,223	17,672	137,100	2,224
Thailand	52,894	16,314	171,499	53,672	16,626	173,261	778	54,504	16,962	175,139	1,610	55,397	17,323	177,150	2,503
Timor-Leste	7,888	2,833	21,967	8,004	2,886	22,204	116	8,129	2,942	22,458	240	8,262	3,003	22,729	373
Togo	67,490	24,571	185,380	68,482	25,025	187,402	992	69,544	25,513	189,562	2,054	70,684	26,039	191,876	3,194
Tonga	298	94	940	302	96	950	4	307	98	960	9	312	100	971	14
Tunisia	18,764	5,945	59,217	19,039	6,059	59,830	276	19,335	6,181	60,484	571	19,652	6,312	61,184	888
Turkey	68,971	19,652	242,060	69,985	20,031	244,522	1,014	71,070	20,437	247,147	2,099	72,235	20,876	249,955	3,264
Turkmenistan	10,026	3,057	32,882	10,174	3,116	33,219	147	10,331	3,179	33,580	305	10,501	3,246	33,965	474
Uganda	415,087	149,123	1,155,405	421,189	151,906	1,167,830	6,102	427,718	154,893	1,181,093	12,631	434,731	158,111	1,195,303	19,644
Ukraine	33,813	10,979	104,134	34,310	11,187	105,228	497	34,842	11,410	106,395	1,029	35,413	11,650	107,645	1,600
United Republic of Tanzania	523,317	187,417	1,461,240	531,010	190,917	1,476,931	7,693	539,241	194,674	1,493,681	15,925	548,083	198,723	1,511,625	24,766
Uzbekistan	71,320	23,685	214,757	72,368	24,132	217,024	1,048	73,490	24,611	219,441	2,170	74,695	25,128	222,031	3,375
Vanuatu	1,234	406	3,753	1,252	413	3,793	18	1,272	422	3,835	38	1,292	431	3,879	58
Venezuela (Bolivarian Republic of)	39,457	10,678	145,805	40,037	10,877	147,375	580	40,658	11,090	149,051	1,201	41,324	11,321	150,846	1,867
Viet Nam	193,749	65,629	571,977	196,597	66,863	578,053	2,848	199,644	68,187	584,536	5,896	202,918	69,614	591,481	9,169
Yemen	188,879	69,331	514,566	191,656	70,618	520,149	2,777	194,627	71,999	526,110	5,748	197,818	73,487	532,498	8,939
Zambia	125,984	43,264	366,868	127,836	44,079	370,745	1,852	129,818	44,955	374,883	3,834	131,946	45,898	379,313	5,962
Zimbabwe	81,231	28,492	231,589	82,425	29,026	234,066	1,194	83,703	29,598	236,710	2,472	85,075	30,215	239,542	3,844
**Total**	**19,250,634**			**19,533,631**			**282,996**	**19,836,436**			**585,802**	**20,161,661**			**911,026**

Source: Authors' elaboration
