# Supplementary material for: Estimated impact of the 2020 economic downturn on under-5 mortality for 129 countries
Source: PLoS One. 2022 Feb 23;17(2):e0263245. doi: 10.1371/journal.pone.0263245 (PMC8865697; doi:10.1371/journal.pone.0263245)
Supplement: S6 Appendix — (ZIP) [file pone.0263245.s006.zip › S6 Appendix.pdf]

S6 Appendix

Estimated Under-5 Lives Lost from 2020 Downturns Scaled from 5% to 15%.

	**Scenario 1**	**Scenario 2**	**Scenario 3**	**Scenario 4**
	**No Downturn**	**5% Downturn**	**10% Downturn**	**15% Downturn**
Country	Under-5 mortality rate per 100	Under-5 mortality rate per 100	Under-5 mortality rate per 100	Under-5 mortality rate per 100
Burundi	9.68	9.82	9.97	10.14
Niger	9.64	9.78	9.93	10.09
Democratic Republic of the Congo	8.77	8.9	9.04	9.18
Central African Republic	7.33	7.44	7.55	7.68
Mali	7.15	7.26	7.37	7.49
Chad	6.54	6.63	6.73	6.84
Mozambique	6.37	6.46	6.56	6.67
Burkina Faso	6.3	6.4	6.5	6.6
Somalia	6.14	6.23	6.32	6.43
Sierra Leone	6.13	6.22	6.31	6.42

Source: Authors' elaboration
