# Supplementary material for: Estimated impact of the 2020 economic downturn on under-5 mortality for 129 countries
Source: PLoS One. 2022 Feb 23;17(2):e0263245. doi: 10.1371/journal.pone.0263245 (PMC8865697; doi:10.1371/journal.pone.0263245)
Supplement: S7 Appendix — (ZIP) [file pone.0263245.s007.zip › S7 Appendix.pdf]

S7 Appendix

Sensitivity analysis of incremental deaths (95% Confidence Intervals).

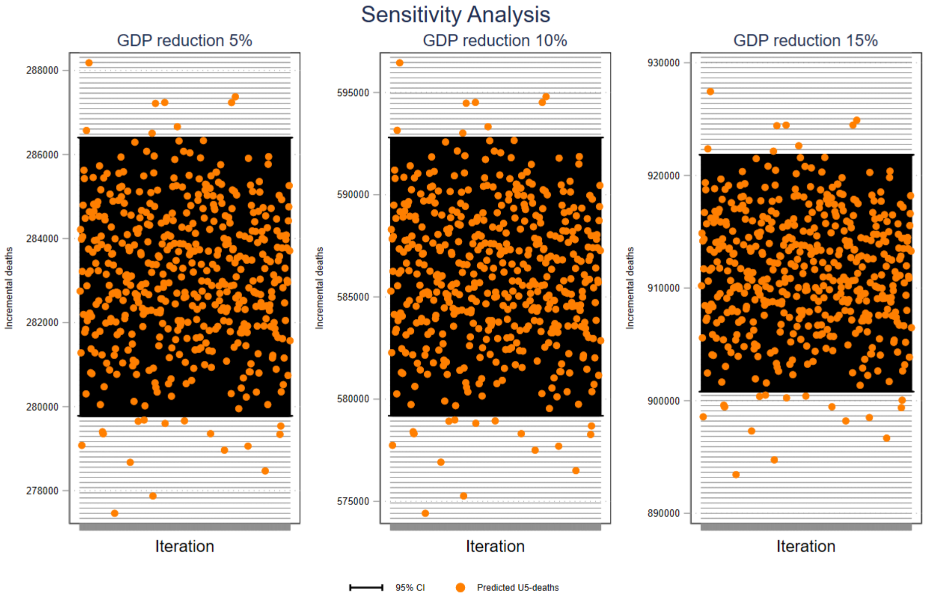

Source: Authors' elaboration
