# Supplementary material for: Estimated impact of the 2020 economic downturn on under-5 mortality for 129 countries
Source: PLoS One. 2022 Feb 23;17(2):e0263245. doi: 10.1371/journal.pone.0263245 (PMC8865697; doi:10.1371/journal.pone.0263245)

PLOS ONE

Estimated impact of the 2020 economic downturn on under-5 mortality for 129 countries

--Manuscript Draft--

**Manuscript Number:**	PONE-D-21-22130
**Article Type:**	Research Article
**Full Title:**	Estimated impact of the 2020 economic downturn on under-5 mortality for 129 countries
**Short Title:**	Impact of the economic downturn on under-5 mortality
**Corresponding Author:**	Marcelo Cardona, MSc<br>Rockwool Fonden<br>Copenhagen, DENMARK
**Keywords:**	Under-5 mortality; economic recession; low- and middle-income countries
**Abstract:**	In low- and middle-income countries (LMICs), economic recessions and downturns can lead to increased child mortality by affecting dietary, environmental, and care-seeking factors. This study estimates the potential loss of life in children under five years old attributable to economic downturns in 2020. We used a multi-level, mixed effects model to estimate the relationship between gross domestic product (GDP) per capita and under-5 mortality rates (U5MRs) specific to each of 129 LMICs . Public data were retrieved from the World Bank World Development Indicators database and the United Nations World Populations Prospects estimates for the years 1990-2020. Each country's individual slope relationship between child mortality and GDP was used to estimate the impact on U5MR of reductions in GDP per capita of 5%, 10%, and 15%. A 5% reduction in GDP per capita in 2020 was estimated to cause an additional 282,996 deaths in children under 5 in 2020. Recessions at 10% and 15% lead to higher losses of under-5 lives, increasing to 585,802 and 911,026 additional deaths, respectively. Nearly half of all the potential under-5 lives lost in LMICs were estimated to occur in Sub-Saharan Africa. Because most of these deaths will likely be due to nutrition and environmental factors amenable to intervention, countries should ensure continued investments in food supplementation, growth monitoring, and comprehensive primary health care to mitigate protentional burdens.
**Order of Authors:**	Marcelo Cardona, MSc
	Joseph Millward
	Katelyn Yoo
	Alison Gemmill
	David Bishai
**Additional Information:**	
**Question**	**Response**
**Financial Disclosure**<br><br>Enter a financial disclosure statement that describes the sources of funding for the work included in this submission. Review the submission guidelines for detailed requirements. View published research articles from *PLOS ONE* for specific examples.<br><br>This statement is required for submission and **will appear in the published article** if	A grant from Big Win Philanthropy (https://www.bigwin.org/) partially funded this study. A.G and D.B. were partially supported by the Robert Wood Johnson Foundation (https://www.rwjf.org/).<br>The funder/sponsor did not participate in the work.

Powered by Editorial Manager® and ProduXion Manager® from Aries Systems Corporation

the submission is accepted. Please make sure it is accurate.<br><br>**Unfunded studies**<br>Enter: *The author(s) received no specific funding for this work.*<br><br>**Funded studies**<br>Enter a statement with the following details:<br>• Initials of the authors who received each award<br>• Grant numbers awarded to each author<br>• The full name of each funder<br>• URL of each funder website<br>• Did the sponsors or funders play any role in the study design, data collection and analysis, decision to publish, or preparation of the manuscript?<br>• **NO** - Include this sentence at the end of your statement: *The funders had no role in study design, data collection and analysis, decision to publish, or preparation of the manuscript.*<br>• **YES** - Specify the role(s) played.<br><br>* typeset	
**Competing Interests**<br><br>Use the instructions below to enter a competing interest statement for this submission. On behalf of all authors, disclose any competing interests that could be perceived to bias this work—acknowledging all financial support and any other relevant financial or non-financial competing interests.<br><br>This statement is required for submission and **will appear in the published article** if the submission is accepted. Please make sure it is accurate and that any funding sources listed in your Funding Information later in the submission form are also declared in your Financial Disclosure statement.<br><br>View published research articles from *PLOS ONE* for specific examples.	The authors have declared that no competing interests exist.

**NO authors have competing interests**<br><br>Enter: *The authors have declared that no competing interests exist.*<br><br>**Authors with competing interests**<br><br>Enter competing interest details beginning with this statement:<br><br>*I have read the journal's policy and the authors of this manuscript have the following competing interests: [insert competing interests here]*<br><br>* typeset	
**Ethics Statement**<br><br>Enter an ethics statement for this submission. This statement is required if the study involved:<br><br>• Human participants<br>• Human specimens or tissue<br>• Vertebrate animals or cephalopods<br>• Vertebrate embryos or tissues<br>• Field research<br><br>Write "N/A" if the submission does not require an ethics statement.<br><br>General guidance is provided below. Consult the submission guidelines for detailed instructions. **Make sure that all information entered here is included in the Methods section of the manuscript.**	N/A

**Format for specific study types**<br><br>**Human Subject Research (involving human participants and/or tissue)**<br>• Give the name of the institutional review board or ethics committee that approved the study<br>• Include the approval number and/or a statement indicating approval of this research<br>• Indicate the form of consent obtained (written/oral) or the reason that consent was not obtained (e.g. the data were analyzed anonymously)<br><br>**Animal Research (involving vertebrate animals, embryos or tissues)**<br>• Provide the name of the Institutional Animal Care and Use Committee (IACUC) or other relevant ethics board that reviewed the study protocol, and indicate whether they approved this research or granted a formal waiver of ethical approval<br>• Include an approval number if one was obtained<br>• If the study involved *non-human primates*, add *additional details* about animal welfare and steps taken to ameliorate suffering<br>• If anesthesia, euthanasia, or any kind of animal sacrifice is part of the study, include briefly which substances and/or methods were applied<br><br>**Field Research**<br><br>Include the following details if this study involves the collection of plant, animal, or other materials from a natural setting:<br>• Field permit number<br>• Name of the institution or relevant body that granted permission	
**Data Availability**<br><br>Authors are required to make all data underlying the findings described fully available, without restriction, and from the time of publication. PLOS allows rare exceptions to address legal and ethical concerns. See the PLOS Data Policy and FAQ for detailed information.	Yes - all data are fully available without restriction

Powered by Editorial Manager® and ProduXion Manager® from Aries Systems Corporation

A Data Availability Statement describing where the data can be found is required at submission. Your answers to this question constitute the Data Availability Statement and **will be published in the article**, if accepted.<br><br>**Important:** Stating 'data available on request from the author' is not sufficient. If your data are only available upon request, select 'No' for the first question and explain your exceptional situation in the text box.<br><br>Do the authors confirm that all data underlying the findings described in their manuscript are fully available without restriction?	
**Describe where the data may be found in full sentences. If you are copying our sample text, replace any instances of XXX with the appropriate details.**<br><br>• If the data are **held or will be held in a public repository**, include URLs, accession numbers or DOIs. If this information will only be available after acceptance, indicate this by ticking the box below. For example: *All XXX files are available from the XXX database (accession number(s) XXX, XXX.)*.<br>• If the data are all contained **within the manuscript and/or Supporting Information files**, enter the following: *All relevant data are within the manuscript and its Supporting Information files.*<br>• If neither of these applies but you are able to provide **details of access elsewhere**, with or without limitations, please do so. For example:<br><br>*Data cannot be shared publicly because of [XXX]. Data are available from the XXX Institutional Data Access / Ethics Committee (contact via XXX) for researchers who meet the criteria for access to confidential data.*<br><br>*The data underlying the results presented in the study are available from (include the name of the third party*	The data underlying the results presented in the study are available from United Nation's World Population Prospects 2019 Revision (https://population.un.org/wpp/#:~:text=The%202019%20Revision%20of%20World%20Population%20Prospects%20is,and%20Social%20Affairs%20of%20the%20United%20Nations%20Secretariat.) and from the World Bank World Development Indicators (https://databank.worldbank.org/reports.aspx?source=world-development-indicators)

Powered by Editorial Manager® and ProduXion Manager® from Aries Systems Corporation

and contact information or URL).<br>• This text is appropriate if the data are owned by a third party and authors do not have permission to share the data.<br><br>* typeset	
Additional data availability information:	Tick here if the URLs/accession numbers/DOIs will be available only after acceptance of the manuscript for publication so that we can ensure their inclusion before publication.

Powered by Editorial Manager® and ProduXion Manager® from Aries Systems Corporation

Manuscript Click here to access/download;Manuscript;Manuscript.docx

Estimated impact of the 2020 economic downturn on under-5 mortality for 129 countries

Marcelo Cardona[1¶*], Joseph Millward[2, 4 ¶], Katelyn Jison Yoo[3, 4 ¶], Alison Gemmill[5 ¶], David M. Bishai[5]

Affiliations:

1 The Rockwool Foundation, Copenhagen, Denmark
2 Johns Hopkins University Center for Communications Programs, Baltimore, Maryland.
3 Health, Nutrition, and Population, World Bank, Washington, DC.
4 Johns Hopkins Bloomberg School of Public Health, Baltimore, Maryland.
5 Department of Population, Family and Reproductive Health, Johns Hopkins Bloomberg School of Public Health, Baltimore, Maryland.

***Corresponding author:**

E-mail: mcc@rff.dk (MC)

[¶]These authors contributed equally to this work

ABSTRACT

In low- and middle-income countries (LMICs), economic recessions and downturns can lead to increased child mortality by affecting dietary, environmental, and care-seeking factors. This study estimates the potential loss of life in children under five years old attributable to economic downturns in 2020. We used a multi-level, mixed effects model to estimate the relationship between gross domestic product (GDP) per capita and under-5 mortality rates (U5MRs) specific to each of 129 LMICs. Public data were retrieved from the World Bank World Development Indicators database and the United Nations World Populations Prospects estimates for the years 1990-2020. Each country's individual slope relationship between child mortality and GDP was used to estimate the impact on U5MR of reductions in GDP per capita of 5%, 10%, and 15%. A 5% reduction in GDP per capita in 2020 was estimated to cause an additional 282,996 deaths in children under 5 in 2020. Recessions at 10% and 15% lead to higher losses of under-5 lives, increasing to 585,802 and 911,026 additional deaths, respectively. Nearly half of all the potential under-5 lives lost in LMICs were estimated to occur in Sub-Saharan Africa. Because most of these deaths will likely be due to nutrition and environmental factors amenable to intervention, countries should ensure continued investments in food supplementation, growth monitoring, and comprehensive primary health care to mitigate protentional burdens.

Introduction

Economic downturns have occurred in almost all countries as a result of COVID-19. We know from prior research that these economic downturns have a disproportionate effect on child health and mortality in low- and middle-income countries (but not high-income countries) and that these effects are likely independent of whether or not children acquire COVID-19 disease.[1–4]

The mechanisms relating distal social health determinants like GDP per capita to child mortality in low- and middle-income countries are presumed to act through the combined effects of environmental contamination, nutrient deficiency, maternal factors, injury, and personal illness control.[4] For example, reductions in household income can unleash dual effects of environmental contamination and nutrient deficiency as households cope with poverty by moving to poorer housing with less sanitation and more crowding, as well as shifting diets away from costly sources of protein, and micronutrients.[5] A spiral of successive gastrointestinal, skin, and respiratory infections can further deplete nutritional reserves. Parents securing supplemental income during economic hardship can subject children to less parental supervision heightening the risk of injury. Both the demand for and the supply of essential childhood health services including immunizations, micronutrients, and primary care can falter during a severe economic recession. The pediatric community, therefore, plays an essential role in mitigating the health harms of sudden economic downturns by offering growth monitoring, routine vaccinations, and care for respiratory and gastrointestinal infections.

Summary of Comments on PONE-D-21-22130.pdf

Page: 9

Number: 1 [redacted] [redacted]

Are we here already distinguishing between downturn and recession? If so, please define and distinguish between the two definitions at the outset.

Number: 2 [redacted] [redacted]

"Distal" seems like an odd choice of words here. Can this be replaced with something more descriptive?

Number: 3 [redacted] [redacted]

Please define the abbreviation at first use in the main text.

Number: 4 [redacted] [redacted]

Please cite this assertion specifically.

Number: 5 [redacted] [redacted]

Awkward phrasing here...suggest revising to something like "caregivers for children".

Knowing how the global 2020 economic downturn led to worsening child health and mortality can help inform policymakers, clinicians, and advocates about the magnitude of such effects and can improve strategies to reduce disease burden, especially in the face of a prolonged pandemic. To our knowledge, however, [illegible] y ha [illegible] indirect health effects [illegible] he [illegible] onom [illegible] downturn, even though past economic recessions have been shown to lead to health declines, especially among children. For example, studies from Ebola outbreaks in Africa and SARS in East Asia have highlighted the importance of national and international organizations in combating the indirect economic effects of disease on the most economically disadvantaged communities.[6,7] Further, a systematic review of the social and economic burden of seasonal influenza in low- and lower-middle-income countries found that influenza's indirect costs, namely productivity loss, were significantly higher in low and middle income countries (LMICs) than high-income countries.[8]

Prior studies of the indirect death toll due to an epidemic-related recession or downturn may not be relevant to 2020 because prior epidemics did not spur an economic slowdown of the same magnitude as those experienced in 2020. Estimates indicate that the world economy was expected to shrink more than 5% in 2020 alone.[9–12] The economic downturns of 2020 have also been projected to reverse a sustained trend of decline in global poverty, with an estimated 42–66 million additional children falling into extreme poverty.[13,14] Additional estimates suggest that the economic effects of the COVID-19 pandemic could reverse the past 2 to 3 years of progress in infant mortality.[14]

Number: 1 [redacted] [redacted]

Please define here how you are distinguishing direct from indirect health effects.

Number: 2 [redacted] [redacted]

Please define what is meant by "additional". Is this in addition to what was expected?

This study assesses the indirect economic effects of the COVID-19 pandemic by estimating the impact of different economic recession scenarios on under-5 mortality in low, lower-middle, and upper-middle-income countries. While there is some uncertainty about the final magnitude of the economic recessions of 2020, our model projects excess under-5 mortality by country that are relevant for recessions as small as [illegible]% and as large as 15% to be used as reference points. Our approach draws from the empirical relationship between mortality and national income that was first noted by Preston and has been widely documented.[3] [illegible]ecent studies have confirmed the adverse effect of recessions on under-5 mortality, showing that the impact in LMICs is three times larger than in countries with better economic indicators.[15–18]

Methods

Overview and Data Sources

The methodology is presented in three sections. In section one, we present the methods used to re-estimate and update [illegible]reston curves specific to each LMIC [illegible] t [illegible] rom [illegible] hi provides multivariate adjusted estimates of the slope parameter relating GDP and Under-5 mortality individualized to each country's most recent data along with 95% confidence intervals. In the second section, we apply each country's GDP-Under-5 mortality slope parameter to estimate the one-year mortality impact of a 5%, 10%, and 15% reduction in GDP. Finally, using Monte Carlo methods, we obtain uncertainty ranges around these excess mortality projections. [illegible]dmittedly, this method will offer a lower bound estimate of the full health impact of recession because the model excludes delayed mortality effects after one year, economically mediated deaths

Page: 11

Number: 1 [redacted] [redacted]
Does this refer to the proportional constriction of the economy?

Number: 2 [redacted] [redacted]
This last sentence seems like it should either be earlier in the introduction section or elsewhere (e.g., discussion section).

Number: 3 [redacted] [redacted]
This is the first mention of the "Preston curves". Suggest providing more information up front.

Number: 4 [redacted] [redacted]
These last two sentences belong in the discussion section.

in adults, and non-fatal effects on health, social development, and cognition that are known to follow famines and adverse childhood experiences. However, estimates of just the mortality effects of the 2020 downturns can help alert policymakers of the need to plan additional efforts to mitigate the economic threats faced by vulnerable groups.

Because recessions in high income countries are known to have only negligible if any effects on child mortality, we only included 129 countries that were classified as low-, lower-middle-, or upper-middle income in our study based on the 2020 World Bank income classification requiring an income below 12,375 US$. Annual estimates of under-5 mortality for each country were obtained from the United Nation's World Population Prospects 2019 Revision. Data on GDP per capita (constant 2010 US$) were obtained from the World Bank World Development Indicators database. Covariates include country-year-specific characteristics and health-specific services obtained from the World Bank World Development Indicators database. We imputed missing values in GDP per capita using a five-year moving average and in some covariates using multivariate normal regression. (See the Appendix Table 1 for additional description of our imputation approach.) All estimated effects of recessions on under-5 mortality were calculated within a one-year time horizon, meaning that the increased mortality rates presented are representative of different recession scenarios occurring during 2020.

Multilevel Mixed Effects Multivariable Regression Analysis

Regression analysis was used to estimate the Preston curve relationship between national income and under-5 mortality. First, we regressed the U5MR on GDP per capita and a set of socio-

Number: 1 [redacted] [redacted]

This statement requires a reference. Also suggest softening the language so as not to suggest the complete absence of effects in high-income countries...just minimal ones (relatively speaking).

Number: 2 [redacted] [redacted]

Please be precise about how income is defined here? For example, is this annual family income?

economic covariates. A model-based approach using an iterative process was used to fill in missing values in the set of covariates. To estimate country-specific effects of a recession, we applied a multilevel mixed-effect linear regression to the relationship between GDP per capita and U5MR for each country. To control for heterogeneity across countries, the multilevel mixed-effect linear regression included fixed effects for a country's region and income level. (Sensitivity analyses showed that results were not sensitive to inclusion of fixed effects.) A generalized log-linear model was estimated to [1]ase the retransformation of impacts from a log-scale to natural units. Recession estimates were bracketed at 5%, 10%, and 15% reductions in country GDP per capita. Our baseline model to estimate the relationship between GDP per capita and U5MR had the following form:

(1) $$logU5mr_{jt} = \alpha_j + \beta_{1j} logGDP_{jt} + \epsilon_{jt}$$

Where β_{1j} captures a country-specific relationship between GDP and under-5 mortality for years t =1990-2020 in country j. Because equation (1) might omit other factors that are closely related to changes in the under-5 mortality rate, we extend equation (1) to include other country-specific factors that could affect the relationship between GDP and U5MR, as shown in equation (2) below:

(2) $$logU5MR_{jt} = \alpha_j + \beta_{1j} logGDP_{jt} + \beta_2 Z_{jt} + \beta_3 H_{jt} + \epsilon_{jt}$$

Where Z_{jt} represents a vector of country-year-specific characteristics. These control variables were as follows: electric power consumption per capita, the proportion of seats held by women in national parliaments, and total fertility rate for country. The last vector of controls (H_{jt}) captures health-specific services for each country-year and includes: the number of physicians per thousand

Number: 1 [redacted] [redacted]

What does it mean to "ease the retransformation"?

inhabitants and the share of children (between 12 and 23 months) who had been immunized with a diphtheria pertussis and tetanus vaccine (DPT). By measuring GDP effects on mortality net of immunization coverage, our final model (equation 2) isolates the GDP mortality effect primarily through effects on wasting, non-vaccine-related diseases, as well as parental caregiving and injury.

Lives Lost Estimation

Country-specific estimates of β_{1j} were then applied to GDP per capita data to predict an estimated mortality impact under the four different scenarios – no reduction in GDP per capita (scenario 1), 5% reduction (scenario 2), a 10% reduction (scenario 3), and 15% (scenario 4). We estimate potential recession-attributable loss of life by subtracting the deaths observed in scenario 1 from the projected number of deaths under scenarios 2-4.

Estimates of Uncertainty

We carried out additional analyses to e [illegible] i [illegible] [1]ntegrity [illegible] m e [illegible] s m [illegible] ng from a normal distribution parameterized with a mean of β_{1j} and the standard error of β_{1j} at the country level. We performed a Monte Carlo experiment using 500 iterations to draw each country's GDP-U5MR impact parameter from normal distributions based on estimates of the coefficient and standard error estimated from equation (). For the simulation, the estimated log(U5MR) from each scenario was retransformed to a mortality rate and then multiplied by the population of children under 5 to produce an estimated total number of deaths under each scenario. The means and standard deviations of the incremental death projections emerging from each sample of 500

Page: 14

Number: 1 [redacted] [redacted]

I'm not sure how this addresses the integrity of the estimates, which would seem to be driven more by the data sources and analysis rather than the range.

iterations is shown in Table 3. Because the Monte Carlo results emerge from 500 iterations they differ slightly from the single iteration estimates.

Results

Between 1990 and 2019, there has been a sustained trend of decline in global poverty and infant mortality in LMICs. However, as hypothesized above, COVID-19 related economic downturns of 2020 are likely to reverse these positive trends.

Table 1 presents select summary statistics for variables used in the analysis for the years 2010, 2015, and 2019. (Appendix Table 1 presents annual statistics for the entire study period, 1990-2020. Values for 2020 are based on projections from various sources that do not take into account the 2020 pandemic.)

Table 1: Descriptive statistics of main variables in the sample of 129 LMIC countries (Values prior to imputation)

	Year		
	2010	2015	2019
**Under-five mortality rate (deaths under age 5 per 1,000 live births)**			
Mean	66.69	43.52	38.37
Standard Deviation	42.26	32.34	28.81
Share of missing observations	0.00	0.00	0.00
**GDP per capita constant 2010$**			
Mean	2,973	3,738	3,996
Standard Deviation	3,306	3,163	3,273
Share of missing observations	2.33	4.65	10.85
**Physicians (per 1,000 people)**			
Mean	0.61	1.19	1.17
Standard Deviation	0.90	1.43	1.37
Share of missing observations	17.05	53.49	100.00
**Electric power consumption (kWh per capita)**			
Mean	955	1,384	1,467
Standard Deviation	1,279	1,366	1,369
Share of missing observations	32.56	100.00	100.00
**Proportion of seats held by women in national parliaments (%)**			
Mean	14.80	19.76	22.65
Standard Deviation	10.70	12.16	12.33
Share of missing observations	3.88	2.33	0.78
**Total fertility (live births per woman)**			
Mean	4.10	3.28	3.11
Standard Deviation	1.51	1.35	1.24
Share of missing observations	0.00	0.00	0.00
**Immunization, DPT (% of children ages 12-23 months)**[1]			
Mean	81.69	84.74	88.24
Standard Deviation	15.60	16.37	18.41
Share of missing observations	0.78	0.00	100.00

Source: Authors' elaboration
Note: For a detailed description for every year, see Appendix Table 1

[1] Child immunization, DPT, measures the percentage of children ages 12-23 months who received DPT vaccinations before 12 months or at any time before the survey. A child is considered adequately immunized against diphtheria, pertussis (or whooping cough), and tetanus (DPT) after receiving three doses of vaccine. [19]

Under-5 Mortality

The results from fitting models of U5MR and GDP for each country are shown in the Appendix Figure 3. Our baseline projection is a benchmark where there is no reduction in GDP per capita (i.e., Scenario 1), and in this case the expected total number of annual under-5 lives lost in LMICs would be around 19.2 million. Under a conservative recession scenario (5% reduction on GDP per capita; Scenario 2), the total number of under-5 deaths increases to 19.5 million, or an additional 282,996 number of deaths (95% CI: 279,779-286,400). The results for each recession scenario at the country level suggest that for the scenarios of 10% and 15% GDP reductions, there is an estimated under-5 loss of life of 19.8 and 20.2 million, which corresponds to an additional 585,802 (95% CI: 579,184-592,799) and 911,026 (95% CI: 900,804-921,825) lives lost, respectively. Moreover, we estimate that 49% of the total under-5 lives lost would occur in Sub-Saharan Africa, a pattern that is observed across the four scenarios, where the total number of lives lost in this region increased up to over 470,000 between a no recession scenario and a 15% reduction in GDP per capita.

The estimated number of deaths is the largest in countries with a higher population. Consequently,

Table 2 presents results for the ten countries with the highest additional under-5 lives lost in 2020 under the four different scenarios[2]. Results suggest that India will be the country with the highest number of under-5 lives lost, followed by Nigeria and the Democratic Republic of the Congo. Moreover, estimates for the top 10 countries with the highest under-5 mortality rates are presented in Appendix Table 4.

[2] See Appendix Table 4 for the complete table

Number: 1 [redacted] [redacted]

Certainly all lives lost are meaningful, but would it not be relevant to first present as a proportion of the general population or of the under-5 general population?

Table 2: Estimated Under-5 Lives Lost from 2020 Recessions Scaled from 5% to 15%.

Country	Under 5 deaths	Lower bound (95% CI)	Upper bound (95% CI)	Under 5 deaths 5% reduction on GDP	Additional deaths 5%	Lower bound (95% CI)	Upper bound (95% CI)	Under 5 deaths 10% reduction on GDP	Additional deaths 10%	Lower bound (95% CI)	Upper bound (95% CI)	Under 5 deaths 15% reduction on GDP	Additional deaths 15%	Lower bound (95% CI)	Upper bound (95% CI)
India	2,929,298	986,082	8,701,895	2,972,361	43,063	1,004,659	8,793,951	3,018,437	89,139	1,024,604	8,892,182	3,067,926	138,628	1,046,101	8,997,378
Nigeria	1,503,219	497,646	4,540,714	1,525,317	22,098	507,077	4,588,238	1,548,962	45,743	517,205	4,638,937	1,574,358	71,139	528,124	4,693,221
Democratic Republic of the Congo	1,388,004	524,706	3,671,682	1,408,409	20,405	534,338	3,712,285	1,430,241	42,237	544,670	3,755,652	1,453,691	65,687	555,796	3,802,143
China	1,235,908	372,924	4,095,918	1,254,076	18,169	380,064	4,138,001	1,273,517	37,609	387,734	4,182,878	1,294,396	58,489	396,005	4,230,905
Pakistan	1,054,683	371,239	2,996,334	1,070,187	15,505	378,185	3,028,413	1,086,777	32,094	385,641	3,062,651	1,104,595	49,912	393,676	3,099,329
Ethiopia	992,985	369,329	2,669,755	1,007,582	14,598	376,148	2,698,999	1,023,202	30,217	383,463	2,730,228	1,039,977	46,993	391,343	2,763,698
United Republic of Tanzania	523,317	187,417	1,461,240	531,010	7,693	190,917	1,476,931	539,241	15,925	194,674	1,493,681	548,083	24,766	198,723	1,511,625
Indonesia	461,840	147,090	1,450,106	468,629	6,789	149,891	1,465,156	475,893	14,054	152,899	1,481,208	483,696	21,856	156,142	1,498,392
Niger	461,338	171,118	1,243,775	468,120	6,782	174,273	1,257,434	475,377	14,039	177,657	1,272,020	483,171	21,833	181,302	1,287,654
Bangladesh	435,117	153,268	1,235,269	441,513	6,396	156,135	1,248,500	448,358	13,241	159,212	1,262,622	455,709	20,592	162,528	1,277,750

Source: Authors' elaboration

Figure 1 presents the number of total additional deaths from a 15% reduction in GDP per capita (e.g. Scenario 4), according to income group classification. Results show that a 15% reduction in GDP per capita will have a substantial increase in the under-5 mortality rate in LMICs, with larger estimated impacts in lower-middle income countries, where under-5 mortality rates tend to be higher.

Figure 1. Changes in under-five mortality from a 15% recession, by country and income group

Sensitivity Analysis and robustness

Table 3 presents the results from the Monte Carlo experiment on the estimated logarithm of U5MR for each country in every scenario. We observe that in all the scenarios, estimations remain between the 95 percent confidence interval, thereby validating the robustness of our approach.

Table 3 Uncertainty analysis from a Monte Carlo experiment using estimates of Model 2.

	**Additional under-5 deaths**					
**Scenario**	Model 1	Model 2	Monte Carlo Version of Model 2 Mean (SD)		Monte Carlo Version of Model 2 95% CI Lower bound	Upper bound
5% Recession	402,847	282,996	283,090	(1,689)	279,779	286,400
10% Recession	837,922	585,802	585,991	(3,473)	579,184	592,799
15% Recession	1,309,822	911,026	911,314	(5,362)	900,804	921,825

Source: Authors' elaboration

Discussion

We estimate that the economic downturns of 2020 significantly increased loss of life among children younger than five years old in LMICs. Many of the countries in this analysis have relatively young populations with tenuous access to stable housing, clean water, food, and primary care. The health of these children is highly susceptible to reductions in the economic well-being of their families. Children in these lower income countries are also subject to a high rate of exposure to other infectious diseases, besides COVID-19, which makes them more susceptible when the economy reduces their access to nutrition, housing, water, sanitation, and parental care.[4] Disruptions to primary health care service supply and demand will compound these threats, and thus may be a likely driver of increased mortality in these settings. Efforts to shore up the delivery of pediatric primary health care services during a recession can mitigate the mortality impact of a recession.

Our estimates match the lower range of other estimates of the indirect effects of the COVID-19 pandemic on child mortality which have primarily focused on excess mortality attributed to disruptions in delivery of key health services affecting children and mothers. Reductions in service delivery could range between 10–52% and the prevalence of wasting could increase by 10–50%.[20] The estimated death toll due to health service reductions was estimated to range from 253,500 to 1,157,000 additional child deaths over a 6 month period with 60% of these deaths, linked to reduced coverage of childbirth services and 18–23% of deaths tied to wasting.[20] Another paper which focused on malaria service delivery disruption found that 25%–75%

reductions in coverage of preventative and curative supplies and care may result in anywhere from 23,600 to 382,100 additional deaths in the most and least conservative scenarios, respectively.[21] In comparison, our analysis finds that 5%–15% reductions in GDP are estimated to lead to additional loss of life in children under five between 282,996 to 911,026. Our estimates are focused on those due to the reduction in GDP and do not include any direct effects of COVID-19 on children. Because our model controls for DPT vaccine delivery (i.e., our model assumes that DPT vaccine delivery is fixed) it underestimates the potential impact of economic recession through these secondary effects on services. We find that the estimated additional lives lost from 5% and 15% recessions would equate to 1.5% and 4.7% increases above baseline, respectively.

The uncertainty surrounding the actual intensity and duration of COVID-19-induced economic effects is a significant limitation of this study. The study aimed to control for uncertainty by offering a bracketed range of likely recession magnitudes from 5% to 15%, which allows countries to situate their own estimated recession rates within this range to customize results. Further limitations exist in the data that were used in this study. For example, many observations from the United Nations Inter-agency Group for Child Mortality Estimation and World Bank World Development Indicators required imputation up to 2020. Measurement of U5MR in many LMICs cannot be based on vital registration systems and must be based on demographic models of survey data produced by the United Nations. Authors also recognize alternative data sources for child mortality such as those available from the University of Washington Institute for Health Metrics and Evaluation, and acknowledge that both datasets are widely used in global health research. In addition, the study only focuses on the lives lost to children under 5 and does not examine other

short- and long-term health-related impacts due to COVID-19 related recessions. Further research should focus on the non-fatal health effects of the 2020 recessions on health, cognition, development, and school attainment.

The empirical evidence correlating health and wealth initially outlined by Samuel Preston, and later expanded by authors such as Angus Deaton, highlighted that mortality in children under-5 is one of the most significantly affected health outcomes from changes in GDP in low and lower-middle income settings.[2,3,22] This should come as no surprise, as the majority of illnesses and complications that affect children under-5 are those that can be largely avoided by routine access to pediatric and post-natal services. Malnutrition and infectious diseases like malaria are particularly lethal for young children, with both of these issues increasing in severity as socioeconomic well-being declines. Further research may benefit from further breaking down under-5 mortality rates into subset rates such as infant mortality and neonatal mortality to even more clearly define areas of intervention. Countermeasures can help to reduce these impacts through food supplementation, growth monitoring, and comprehensive primary health care. Hopefully these estimates of the magnitude of the non-COVID-19 related child mortality can help marshal the resources needed to mitigate the burden.

Acknowledgments

The COVID-Busters – a research team, formed by Dr. David Bishai – has provided invaluable feedback and support for this project. We also thank colleagues and students at the Johns Hopkins Bloomberg School of Public Health for their feedback and insight on this research.

REFERENCES

1. Bishai DM, Cohen R, Alfonso YN, Adam T, Kuruvilla S, Schweitzer J. Factors Contributing to Maternal and Child Mortality Reductions in 146 Low- and Middle-Income Countries between 1990 and 2010. PLOS ONE. 2016;11: e0144908. doi:10.1371/journal.pone.0144908

2. Deaton A. Health, Inequality, and Economic Development. National Bureau of Economic Research; 2001 Jun. Report No.: 8318. doi:10.3386/w8318

3. Preston SH. The Changing Relation between Mortality and Level of Economic Development. Popul Stud. 1975;29: 231–248. doi:10.2307/2173509

4. Mosley WH, Chen LC. An Analytical Framework for the Study of Child Survival in Developing Countries. Popul Dev Rev. 1984;10: 25–45. doi:10.2307/2807954

5. Scrimshaw NS, SanGiovanni JP. Synergism of nutrition, infection, and immunity: an overview. Am J Clin Nutr. 1997;66: 464S-477S. doi:10.1093/ajcn/66.2.464S

6. Bausch DG, Schwarz L. Outbreak of Ebola Virus Disease in Guinea: Where Ecology Meets Economy. PLoS Negl Trop Dis. 2014;8: e3056. doi:10.1371/journal.pntd.0003056

7. Moon S, Sridhar D, Pate MA, Jha AK, Clinton C, Delaunay S, et al. Will Ebola change the game? Ten essential reforms before the next pandemic. The report of the Harvard-LSHTM Independent Panel on the Global Response to Ebola. The Lancet. 2015;386: 2204–2221. doi:10.1016/S0140-6736(15)00946-0

8. de Francisco (Shapovalova) N, Donadel M, Jit M, Hutubessy R. A systematic review of the social and economic burden of influenza in low- and middle-income countries. Vaccine. 2015;33: 6537–6544. doi:10.1016/j.vaccine.2015.10.066

9. Nicola M, Alsafi Z, Sohrabi C, Kerwan A, Al-Jabir A, Iosifidis C, et al. The socio-economic implications of the coronavirus pandemic (COVID-19): A review. Int J Surg Lond Engl. 2020;78: 185–193. doi:10.1016/j.ijsu.2020.04.018

10. Fernandes N. Economic Effects of Coronavirus Outbreak (COVID-19) on the World Economy. Rochester, NY: Social Science Research Network; 2020 Mar. Report No.: ID 3557504. doi:10.2139/ssrn.3557504

11. IMFBlog. The Great Lockdown: Worst Economic Downturn Since the Great Depression. In: IMF Blog [Internet]. [cited 30 Aug 2020]. Available: https://blogs.imf.org/2020/04/14/the-great-lockdown-worst-economic-downturn-since-the-great-depression/

12. Global Economic Prospects, June 2020. The World Bank; 2020. doi:10.1596/978-1-4648-1553-9

13. Decerf B, Ferreira FHG, Mahler DG, Sterck O. Lives and Livelihoods: Estimates of the Global Mortality and Poverty Effects of the Covid-19 Pandemic. The World Bank; 2020. doi:10.1596/1813-9450-9277

14. UNSDG | Policy Brief: The Impact of COVID-19 on children. [cited 10 Sep 2020]. Available: https://unsdg.un.org/resources/policy-brief-impact-covid-19-children

15. Pérez-Moreno S, Blanco-Arana MC, Bárcena-Martín E. Economic cycles and child mortality: A cross-national study of the least developed countries. Econ Hum Biol. 2016;22: 14–23. doi:10.1016/j.ehb.2016.02.005

16. Maruthappu M, Watson RA, Watkins J, Zeltner T, Raine R, Atun R. Effects of economic downturns on child mortality: a global economic analysis, 1981–2010. BMJ Glob Health. 2017;2: e000157. doi:10.1136/bmjgh-2016-000157

17. Tejada CAO, Triaca LM, Liermann NH, Ewerling F, Costa JC, Tejada CAO, et al. Economic crises, child mortality and the protective role of public health expenditure. Ciênc Amp Saúde Coletiva. 2019;24: 4395–4404. doi:10.1590/1413-812320182412.25082019

18. Ensor T, Cooper S, Davidson L, Fitzmaurice A, Graham WJ. The impact of economic recession on maternal and infant mortality: lessons from history. BMC Public Health. 2010;10: 727. doi:10.1186/1471-2458-10-727

19. World Development Indicators | DataBank. [cited 30 Aug 2020]. Available: https://databank.worldbank.org/source/world-development-indicators

20. Roberton T, Carter ED, Chou VB, Stegmuller AR, Jackson BD, Tam Y, et al. Early estimates of the indirect effects of the COVID-19 pandemic on maternal and child mortality in low-income and middle-income countries: a modelling study. Lancet Glob Health. 2020;8: e901–e908. doi:10.1016/S2214-109X(20)30229-1

21. Weiss DJ, Bertozzi-Villa A, Rumisha SF, Amratia P, Arambepola R, Battle KE, et al. Indirect effects of the COVID-19 pandemic on malaria intervention coverage, morbidity, and mortality in Africa: a geospatial modelling analysis. Lancet Infect Dis. 2021;21: 59–69. doi:10.1016/S1473-3099(20)30700-3

22. Piketty T. Capital and Ideology. Harvard University Press; 2020.

APPENDIX

Descriptive statistics

Appendix Table 1 Descriptive Statistics of variables used for the analysis

	Year															
	1990	1991	1992	1993	1994	1995	1996	1997	1998	1999	2000	2001	2002	2003	2004	2005
**Under-five mortality (deaths under age 5 per 1,000 live**																
Mean	102.49	100.82	99.06	97.14	95.01	92.66	90.13	87.48	84.79	82.08	89.90	88.08	82.79	80.78	70.77	76.26
Standard Deviation	71.62	72.23	72.33	71.64	70.09	67.89	65.40	63.02	60.91	59.03	58.80	58.30	55.66	53.62	52.09	49.73
Share of missing observations	0.00	0.00	0.00	0.00	0.00	0.00	0.00	0.00	0.00	0.00	0.00	0.00	0.00	0.00	0.00	0.00
**GDP per capita constant 2010$**																
Mean	2,454	2,431	2,334	2,356	2,331	2,346	2,402	2,475	2,509	2,591	2,451	2,462	2,619	2,614	2,806	2,861
Standard Deviation	2,444	2,444	2,422	2,381	2,369	2,338	2,346	2,430	2,457	2,430	2,430	2,313	2,480	2,531	2,717	2,998
Share of missing observations	13.18	13.18	12.40	11.63	10.85	8.53	8.53	7.75	7.75	6.98	5.43	4.65	3.88	3.88	3.88	3.88
**Physicians (per 1,000 people)**																
Mean	0.96	1.08	1.04	0.95	1.07	1.03	1.02	1.05	1.12	1.09	0.78	0.79	0.87	0.81	0.76	0.82
Standard Deviation	1.30	1.41	1.39	1.33	1.40	1.32	1.31	1.29	1.36	1.34	1.22	1.23	1.26	1.22	1.06	1.21
Share of missing observations	30.23	62.02	61.24	46.51	62.02	42.64	53.49	48.06	62.02	63.57	52.71	58.91	62.02	62.02	31.78	52.71
**Electric power consumption (kWh per capita)**																
Mean	978	963	921	881	889	898	924	933	972	981	824	785	904	864	926	999
Standard Deviation	1,412	1,394	1,327	1,223	1,201	1,167	1,186	1,177	1,201	1,165	1,165	1,103	1,179	1,146	1,142	1,345
Share of missing observations	37.21	36.43	35.66	35.66	35.66	34.88	34.88	34.88	34.88	34.88	33.33	33.33	33.33	33.33	33.33	32.56
**Proportion of seats held by women in national**																
Mean	4.06	4.80	5.23	5.85	6.52	7.44	7.99	8.25	9.12	9.32	9.48	10.28	11.69	12.44	12.44	13.70
Standard Deviation	10.15	10.15	10.09	10.17	10.20	10.33	10.21	7.33	7.72	7.51	8.08	7.90	9.12	9.22	9.03	9.29
Share of missing observations	100.00	100.00	100.00	100.00	100.00	100.00	100.00	20.16	18.60	24.03	18.60	14.73	18.60	10.85	7.75	4.65
**Total fertility (live births per woman)**																
Mean	4.79	4.70	4.61	4.52	4.43	4.34	4.25	4.17	4.09	4.02	4.36	4.36	4.21	4.20	3.92	4.17
Standard Deviation	1.64	1.65	1.66	1.66	1.67	1.68	1.69	1.70	1.70	1.71	1.66	1.59	1.60	1.60	1.58	1.56
Share of missing observations	0.00	0.00	0.00	0.00	0.00	0.00	0.00	0.00	0.00	0.00	0.00	0.00	0.00	0.00	0.00	0.00
**Immunization, DPT (% of children ages 12-23 months)**																
Mean	74.71	71.89	70.88	71.15	72.64	74.45	74.19	74.44	74.89	74.78	72.44	72.99	73.59	76.32	78.10	78.45
Standard Deviation	21.56	22.22	21.69	21.45	21.11	20.55	20.82	22.09	22.02	21.88	20.66	20.91	19.28	18.52	18.24	18.26
Share of missing observations	17.05	16.28	5.43	3.88	3.88	3.88	3.88	3.88	3.88	2.33	2.33	2.33	1.55	1.55	1.55	1.55
**Immunization, measles (% of children ages 12-23 months)**																
Mean	73.39	71.53	69.77	71.77	72.50	74.39	74.59	74.66	75.41	75.68	72.34	73.54	74.62	75.83	77.82	76.74
Standard Deviation	19.28	20.41	20.28	20.21	19.10	19.18	19.59	20.38	20.66	19.90	19.64	19.69	18.69	17.64	18.98	17.85
Share of missing observations	17.05	16.28	5.43	3.88	3.88	3.88	3.88	3.88	3.88	2.33	2.33	2.33	1.55	1.55	1.55	1.55

	Year														
	2006	2007	2008	2009	2010	2011	2012	2013	2014	2015	2016	2017	2018	2019	2020
**Under-five mortality (deaths under age 5 per 1,000 live**															
Mean	73.73	72.08	66.84	64.12	66.69	57.82	57.17	53.73	53.03	43.52	42.07	40.74	39.52	38.37	37.29
Standard Deviation	47.46	46.79	44.43	41.74	42.26	39.49	37.97	34.94	35.01	32.34	31.32	30.41	29.57	28.81	28.09
Share of missing observations	0.00	0.00	0.00	0.00	0.00	0.00	0.00	0.00	0.00	0.00	0.00	0.00	0.00	0.00	0.00
**GDP per capita constant 2010$**															
Mean	2,941	2,995	3,144	3,145	2,973	3,209	3,387	3,362	3,485	3,738	3,782	3,824	3,940	3,996	3,957
Standard Deviation	3,122	3,453	3,419	3,387	3,306	3,178	3,406	3,225	3,342	3,163	3,148	3,228	3,237	3,273	2,835
Share of missing observations	3.88	3.88	3.10	3.10	2.33	3.10	3.88	3.88	3.88	4.65	5.43	5.43	6.20	10.85	100.00
**Physicians (per 1,000 people)**															
Mean	0.88	0.87	0.91	0.73	0.61	0.95	0.93	0.92	0.93	1.19	1.05	1.06	1.14	1.17	1.16
Standard Deviation	1.23	1.19	1.18	1.06	0.90	1.19	1.19	1.15	1.25	1.43	1.39	1.47	1.51	1.37	1.36
Share of missing observations	58.14	48.84	35.66	35.66	17.05	48.84	49.61	47.29	49.61	53.49	48.06	51.94	67.44	100.00	100.00
**Electric power consumption (kWh per capita)**															
Mean	1,017	994	1,050	990	955	1,095	1,105	1,108	1,133	1,384	1,351	1,357	1,414	1,467	1,453
Standard Deviation	1,324	1,335	1,333	1,252	1,279	1,252	1,230	1,239	1,276	1,366	1,361	1,375	1,394	1,369	1,314
Share of missing observations	32.56	32.56	32.56	32.56	32.56	32.56	32.56	32.56	32.56	100.00	100.00	100.00	100.00	100.00	100.00
**Proportion of seats held by women in national**															
Mean	14.13	14.83	15.31	15.32	14.80	16.47	16.71	17.56	18.30	19.76	20.29	20.52	21.17	21.65	22.76
Standard Deviation	9.57	9.83	10.93	10.45	10.70	10.61	10.80	11.37	12.20	12.16	12.36	11.98	12.35	12.33	10.35
Share of missing observations	2.33	3.10	3.88	3.88	3.88	3.10	2.33	6.20	3.88	2.33	0.78	1.55	1.55	0.78	100.00
**Total fertility (live births per woman)**															
Mean	4.12	4.13	3.96	3.92	4.10	3.88	3.84	3.69	3.67	3.28	3.24	3.20	3.16	3.11	3.07
Standard Deviation	1.51	1.56	1.48	1.44	1.51	1.47	1.42	1.32	1.35	1.35	1.32	1.30	1.27	1.24	1.22
Share of missing observations	0.00	0.00	0.00	0.00	0.00	0.00	0.00	0.00	0.00	0.00	0.00	0.00	0.00	0.00	0.00
**Immunization, DPT (% of children ages 12-23 months)**															
Mean	79.60	81.53	81.17	83.22	81.69	83.79	83.54	83.11	81.88	84.74	85.05	84.95	84.70	88.24	88.61
Standard Deviation	18.42	16.14	17.68	15.08	15.60	15.41	16.24	17.81	17.21	16.37	15.87	14.75	15.50	18.41	18.49
Share of missing observations	0.78	0.78	0.78	0.78	0.78	0.00	0.00	0.00	0.00	0.00	0.00	0.00	0.00	100.00	100.00
**Immunization, measles (% of children ages 12-23 months)**															
Mean	78.22	80.04	80.28	81.13	80.43	82.67	82.55	83.03	81.56	84.42	84.20	84.26	84.12	87.70	88.04
Standard Deviation	17.39	16.23	16.62	15.47	14.99	14.37	14.70	15.30	15.43	15.13	15.36	14.67	15.34	17.55	17.49
Share of missing observations	0.78	0.78	0.78	0.78	0.78	0.00	0.00	0.00	0.00	0.00	0.00	0.00	0.00	100.00	100.00

Multiple Imputation

In order to fill in missing values in the independent control variables of interest, we performed multiple imputation using multivariate normal regression. We selected this approach because all the variables used in the analysis are continuous and because it uses an iterative Markov chain Monte Carlo method to impute missing values.

The method uses data augmentation to simulate unobserved missing values. The data augmentation process consists of two steps. In the first step, missing values in x_i a replaced by draws from the conditional posterior distribution of $x_{i(m)}$ given the observed data and current values of model parameters independently for each i. In a second step, the new values of the parameters are drawn from their conditional posterior distribution given the observed data and the data imputed in the previous step. These processes are repeated for a number of iterations, which is determined by the length of the burn-in period and the number of iterations between imputations. The length of the burn-in period must be large enough in order to ensure that the chain converges to the stationary distribution. The number of iterations between imputation should be large enough to ensure that the random draws are approximately independent.

For this study, we imputed 65 additional data-sets, since the largest share of missing values in a variable was around 55%. The number of iterations used in the burn-in period to reach stationarity was 2500. In order to reduce the correlation between sets of imputed values, 900 iterations of the Markov chain Monte Carlo were performed between imputations. We used an informative ridge prior distribution for the Markov chain Monte Carlo procedure; we selected this prior because in

some countries had few observations. Appendix table 2 presents a summary of our multiple imputation and Appendix Figure 1 demonstrates convergence of the Markov chain Monte Carlo algortihm.

Appendix Table 2 Multiple imputation summary

Variable	Complete	Incomplete	Imputed	Total
GDP per capita constant 2010$	3625	374	374	3999
Log GDP per capita constant 2010$	3748	251	251	3999
Physicians (per 1,000 people)	1853	2146	2146	3999
Electric power consumption (kWh per capita)	2132	1867	1867	3999
Proportion of seats held by women in national parliaments (%)	2738	1261	1261	3999
Immunization, DPT (% of children ages 12-23 months)	3639	360	360	3999

Source: Authors' elaboration

Appendix Figure 1 Convergence of the Markov chain Monte Carlo (MCMC)

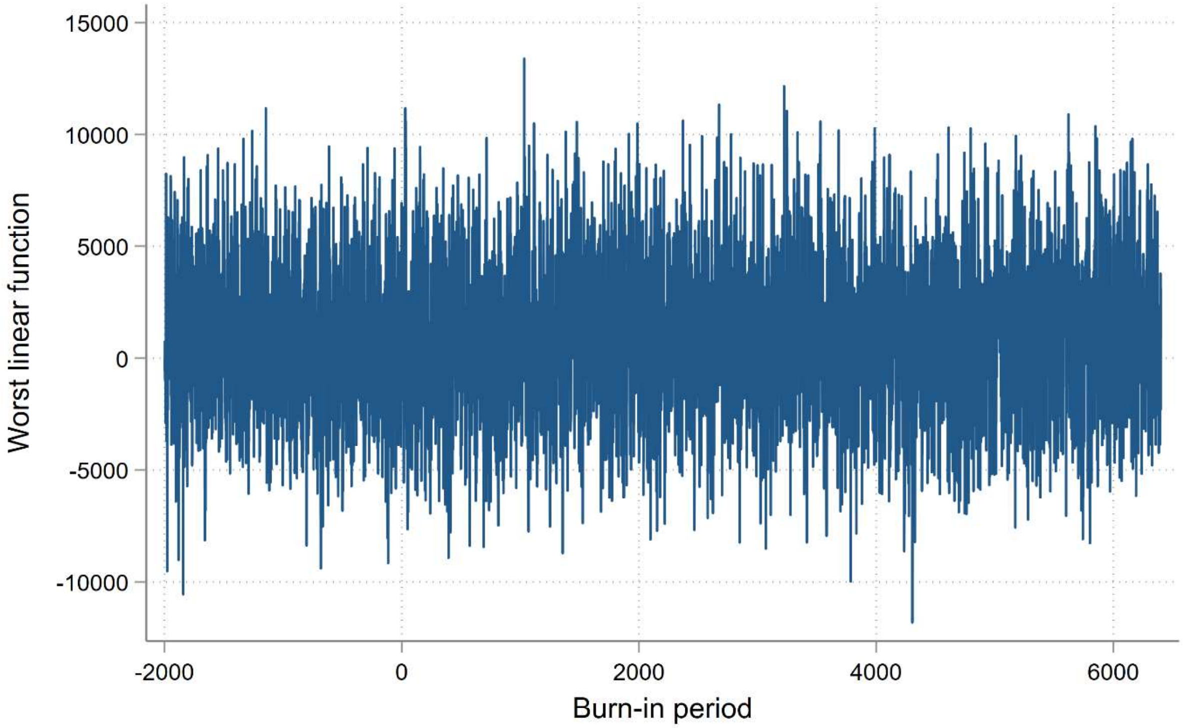

Source: Authors' elaboration

Appendix Figure 2 Autocorrelation of the Worst linear function

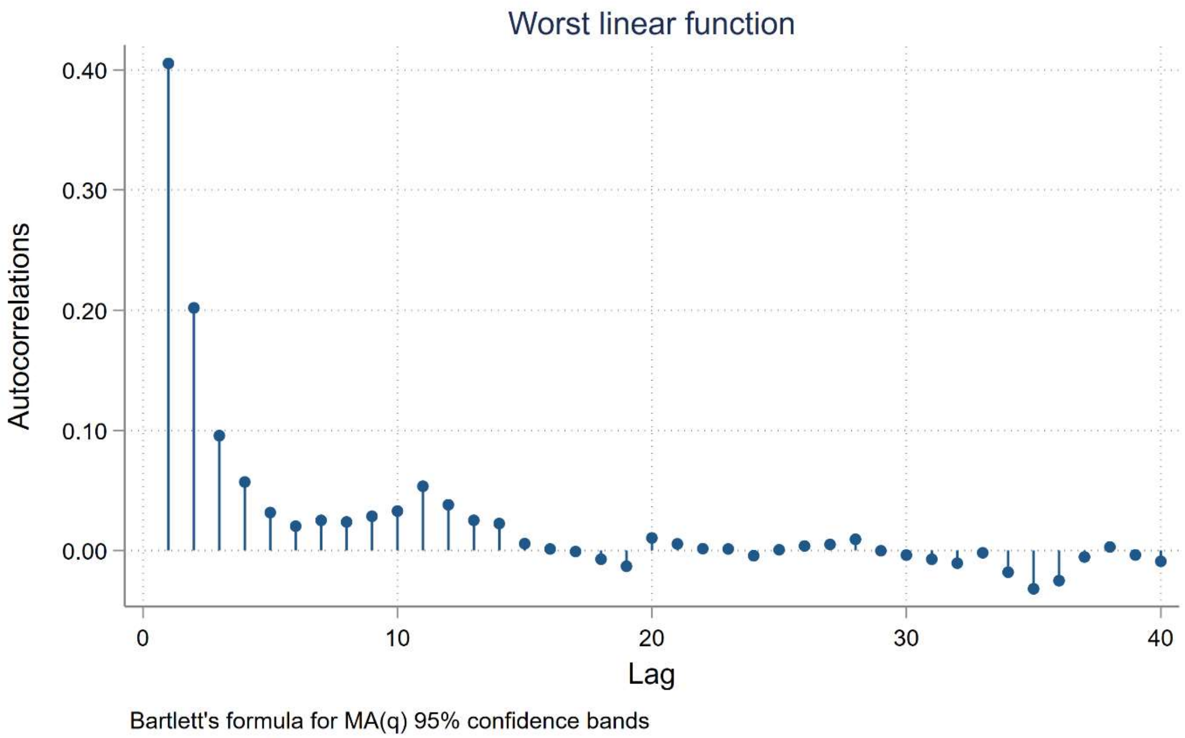

Source: Authors' elaboration

The auto-correlation disappears after 25 lags, which ensures that there is independence between imputations.

Estimation of the U5MR time trajectories at country level

Appendix Figure 3 Estimated effect of GDP reductions on U5MR at country level (fitted values)

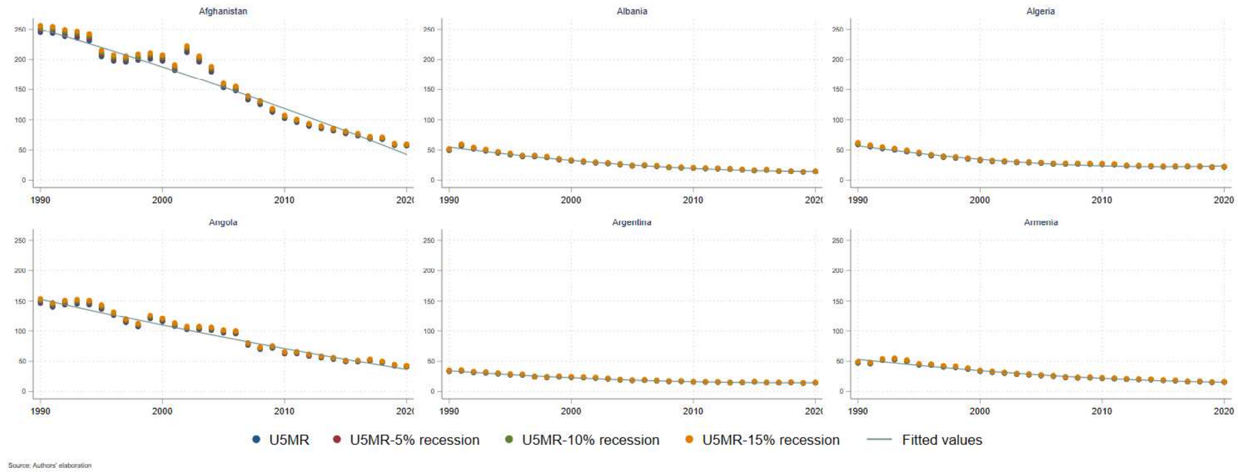

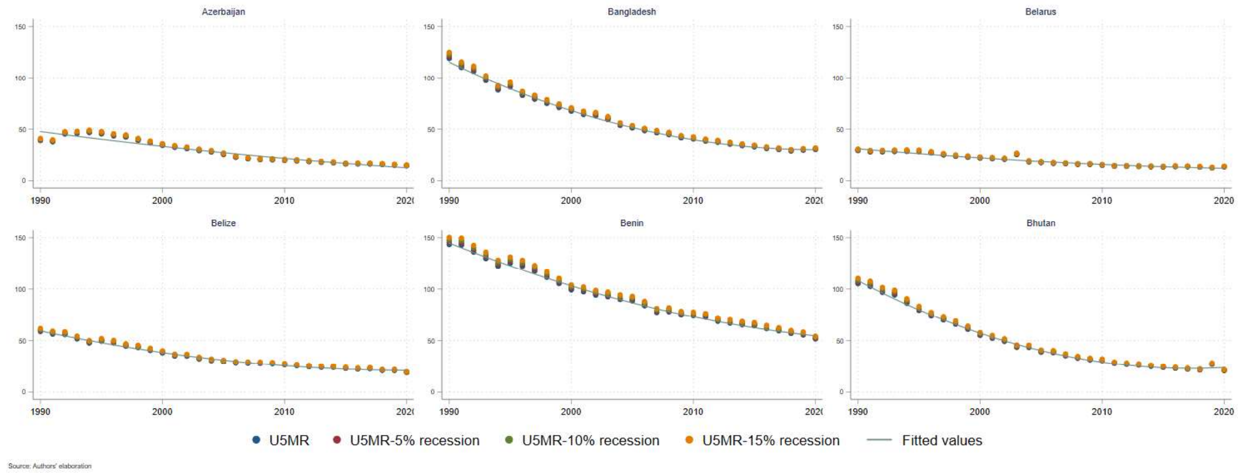

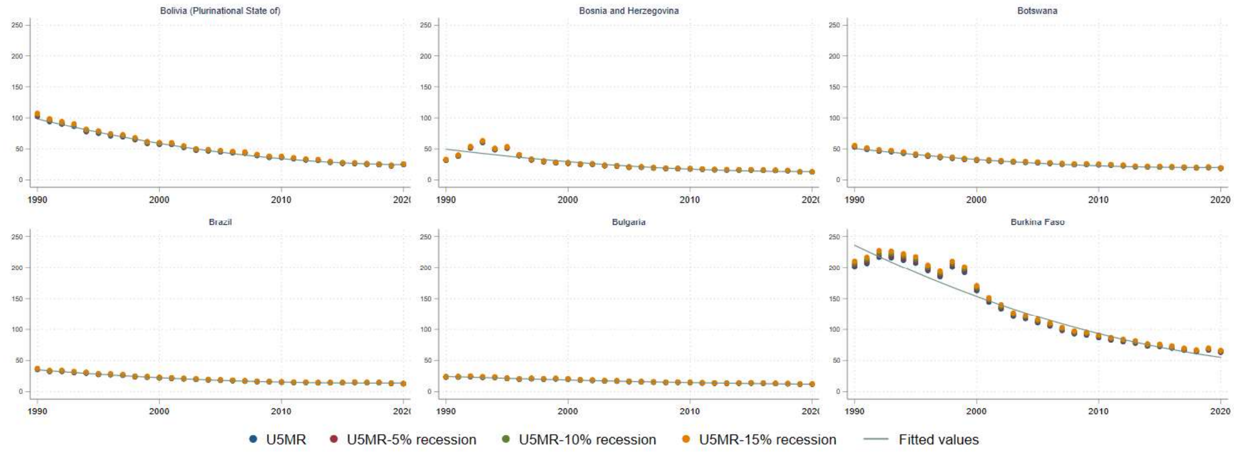

Source: Authors' elaboration

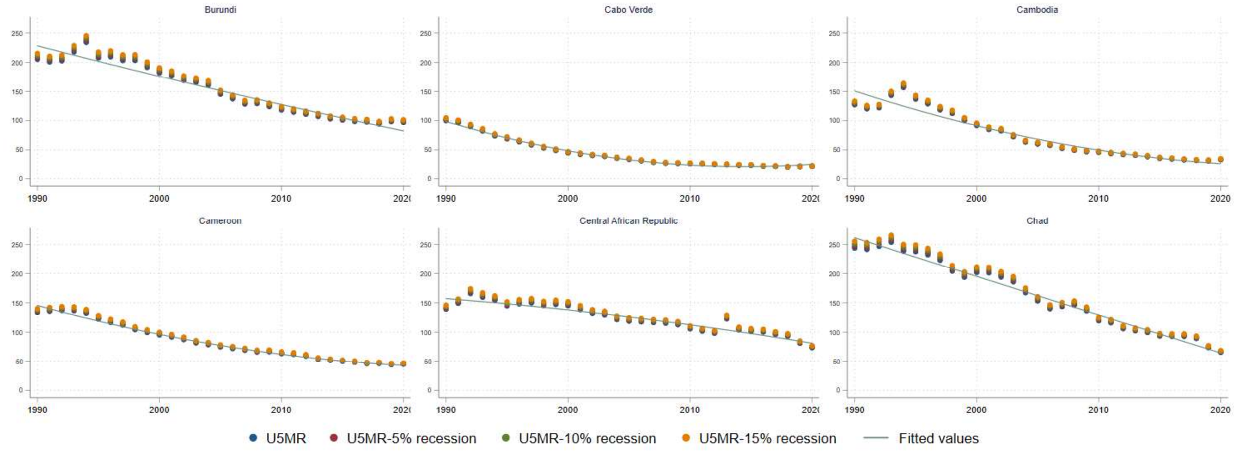

Source: Authors' elaboration

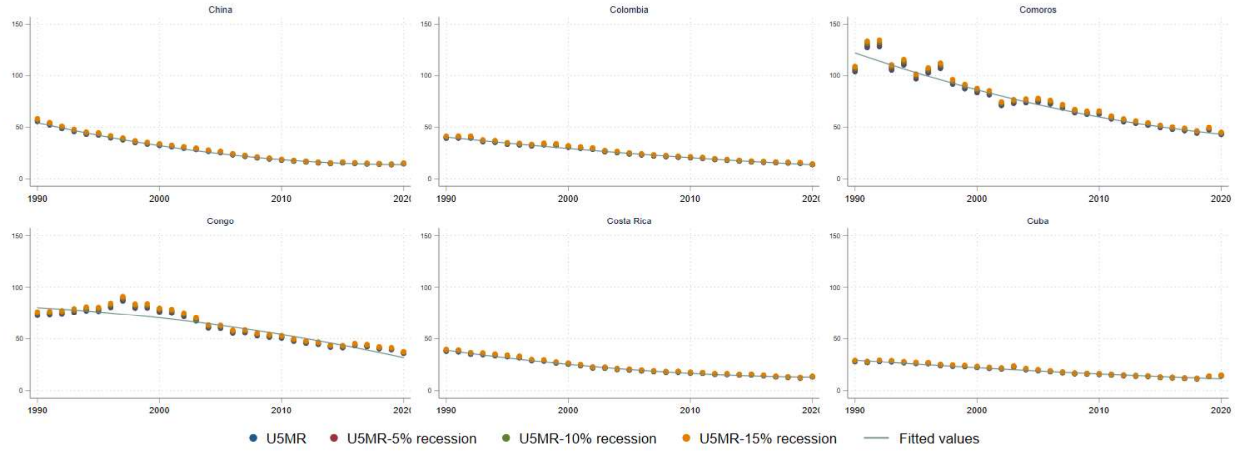

Source: Authors' elaboration

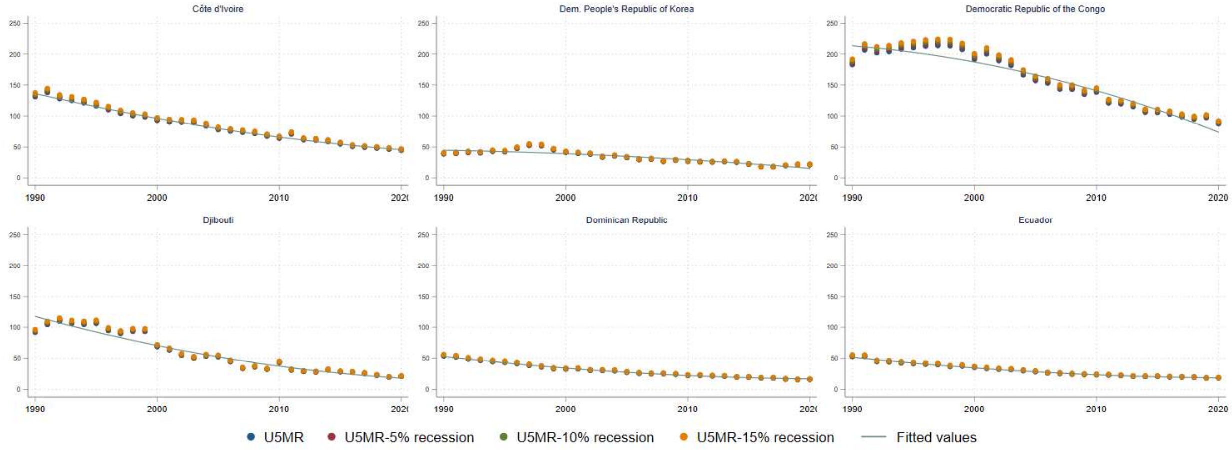

Source: Authors' elaboration

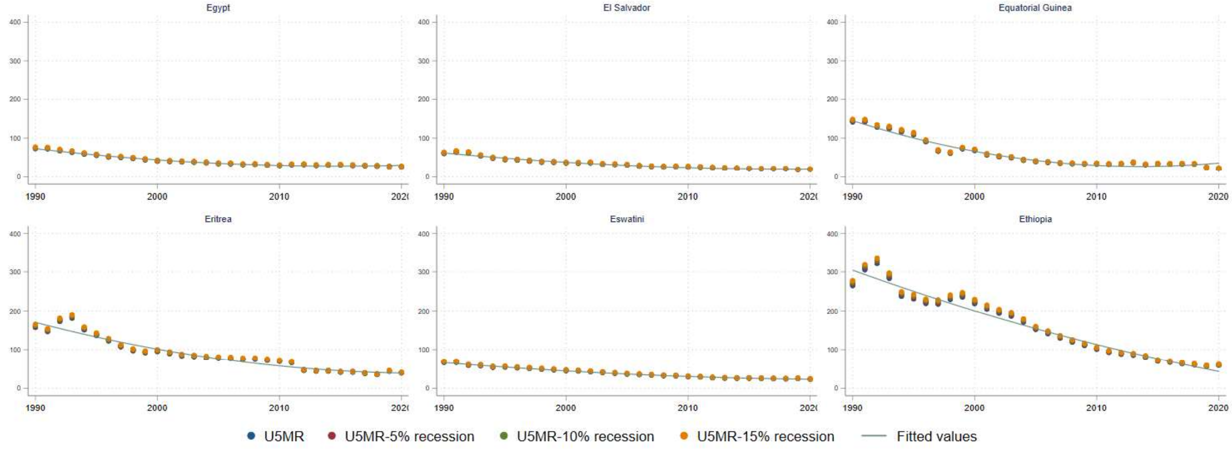

Source: Authors' elaboration

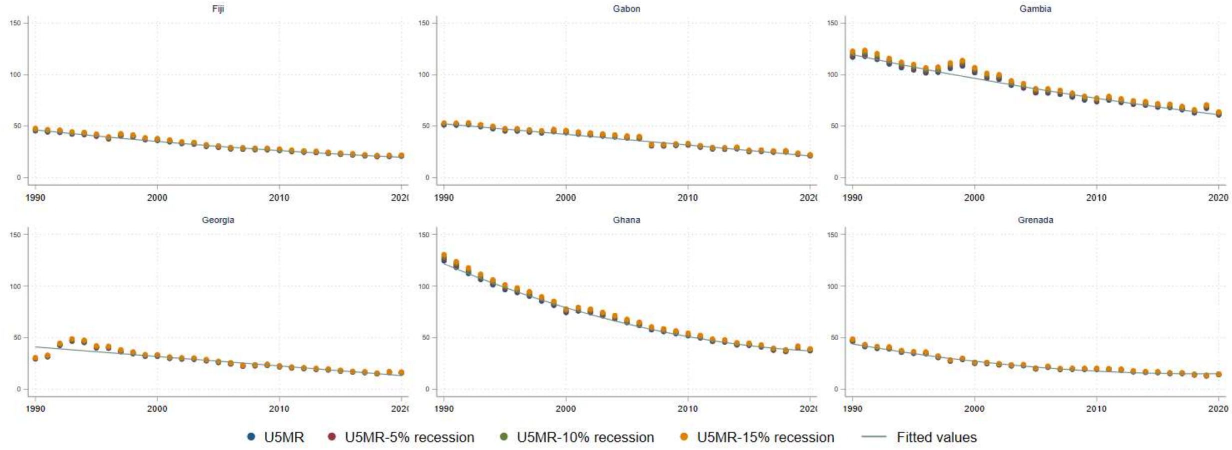

Source: Authors' elaboration

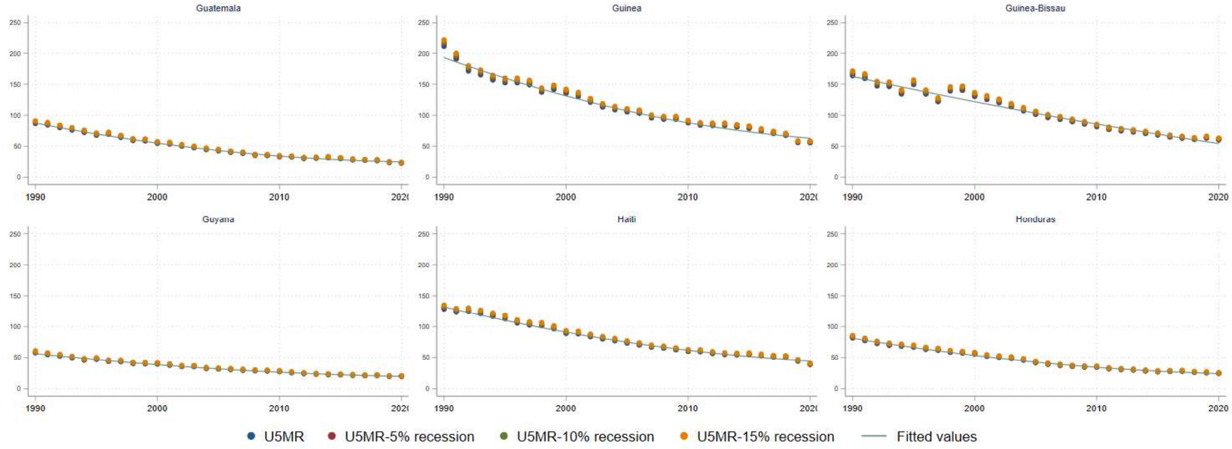

Source: Authors' elaboration

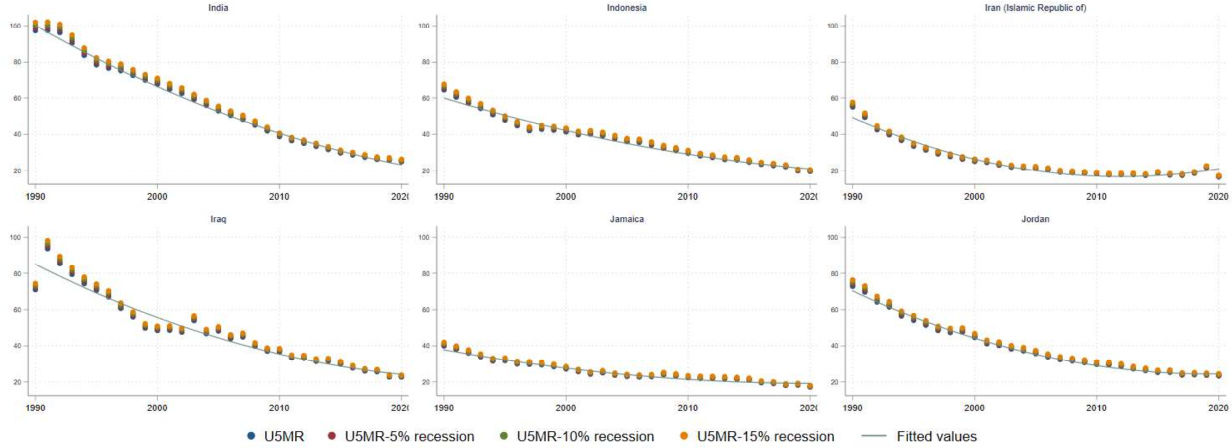

Source: Authors' elaboration

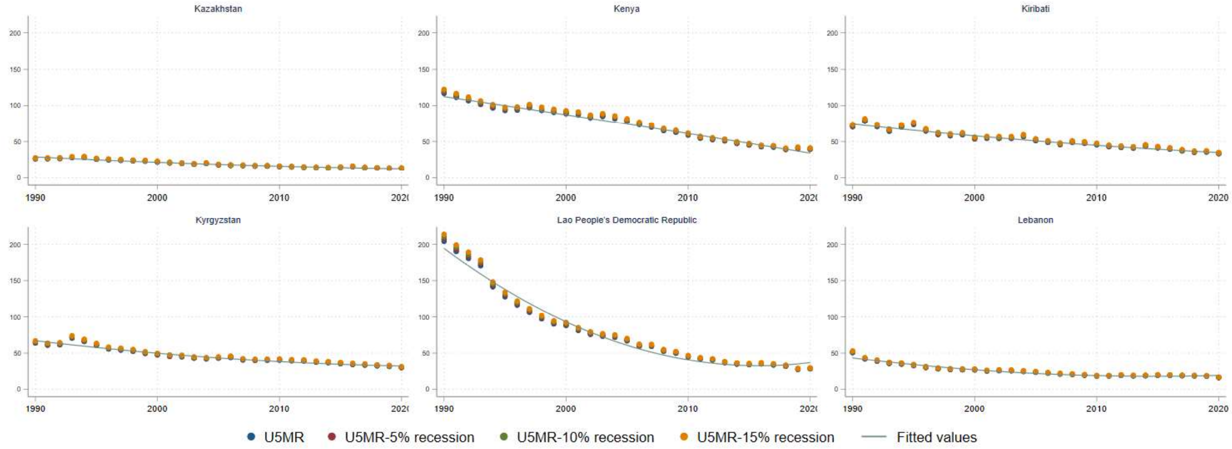

Source: Authors' elaboration

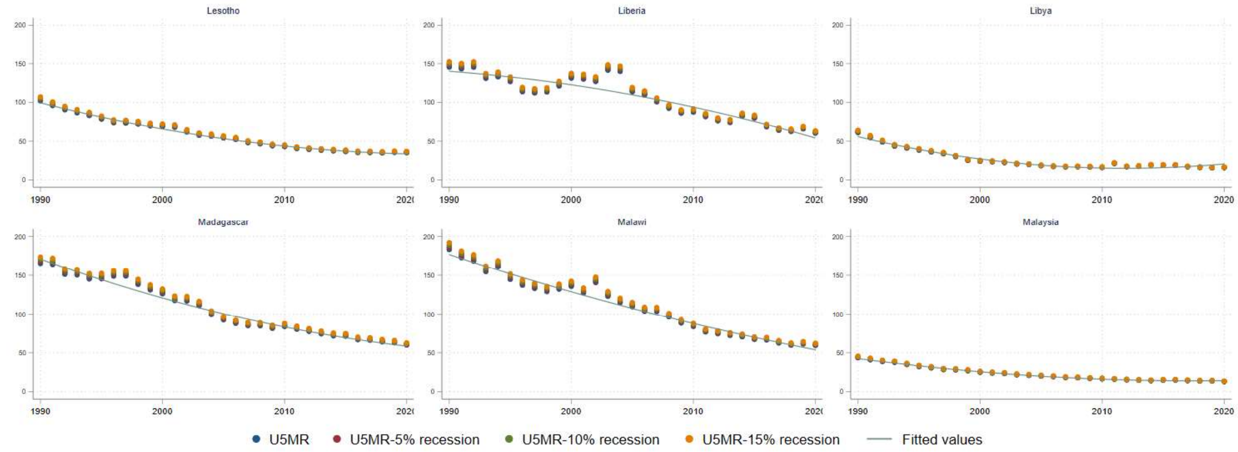

Source: Authors' elaboration

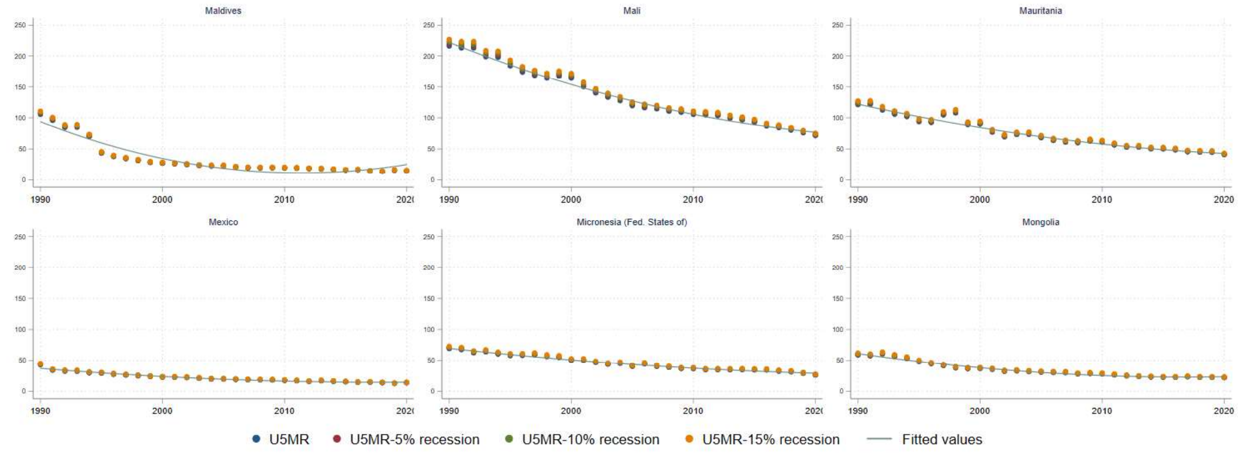

Source: Authors' elaboration

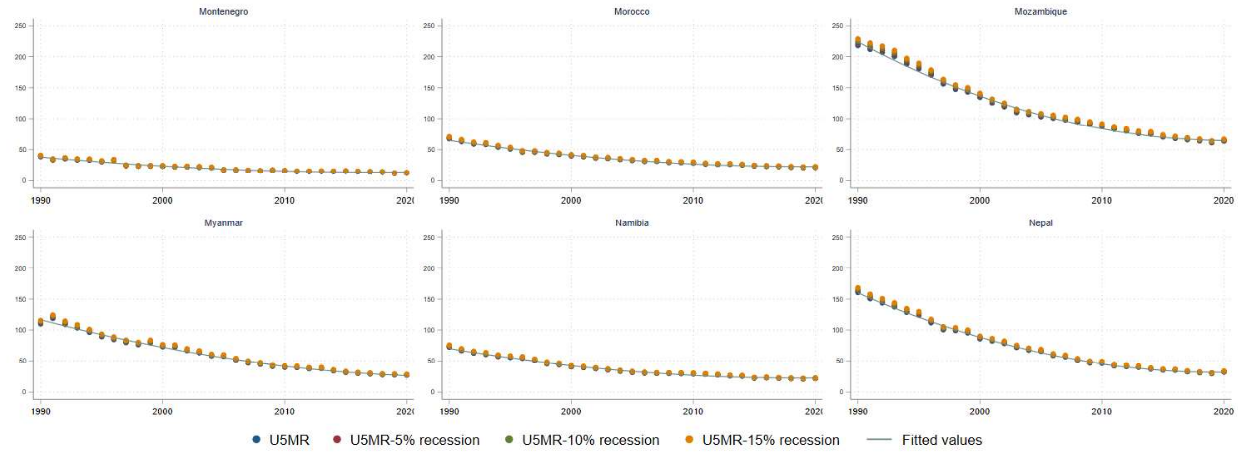

Source: Authors' elaboration

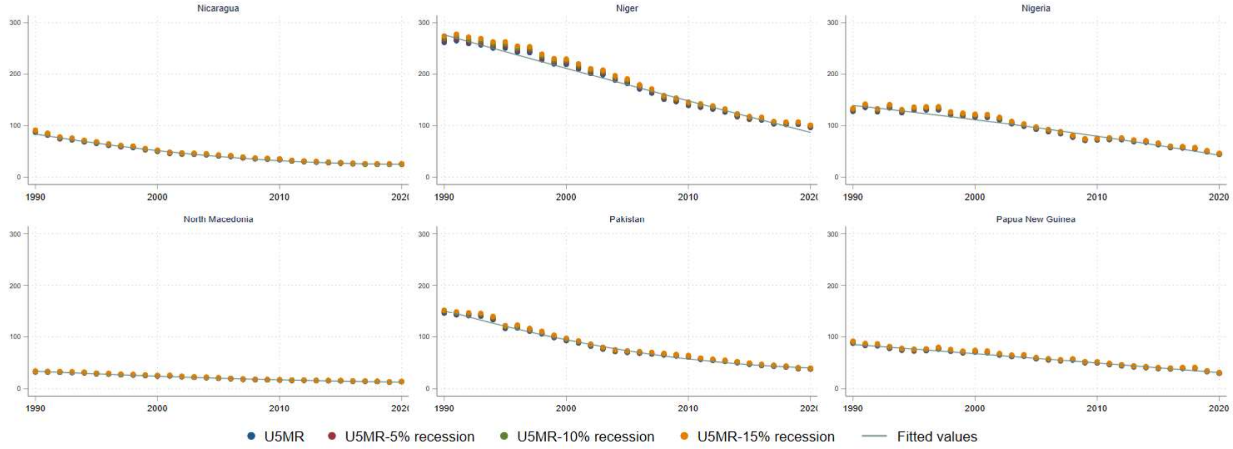

Source: Authors' elaboration

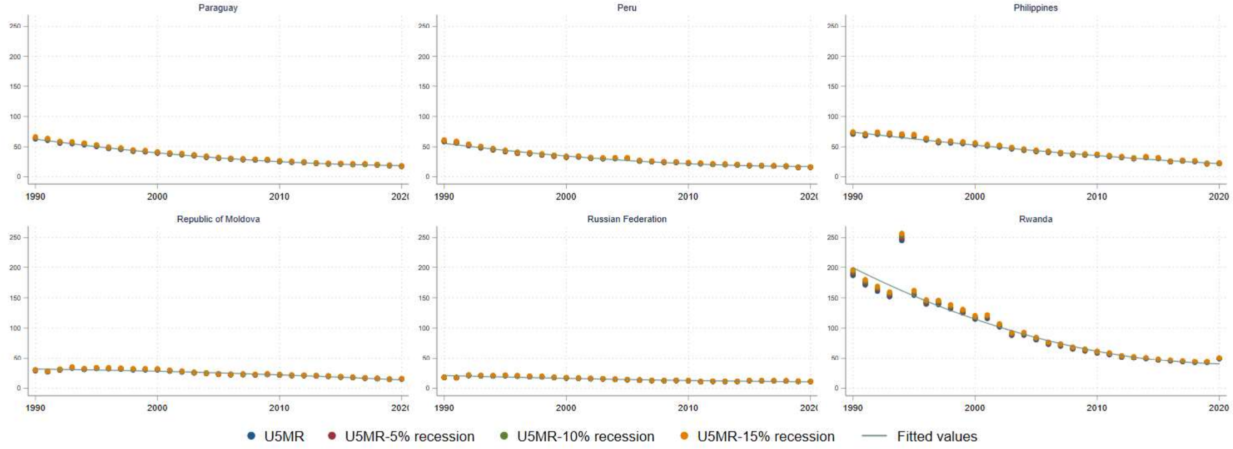

Source: Authors' elaboration

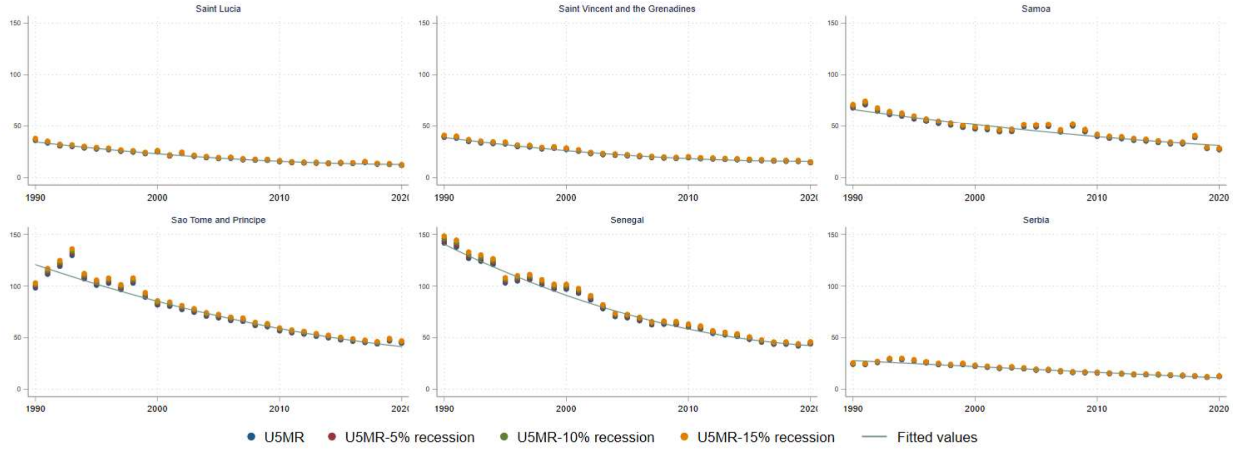

Source: Authors' elaboration

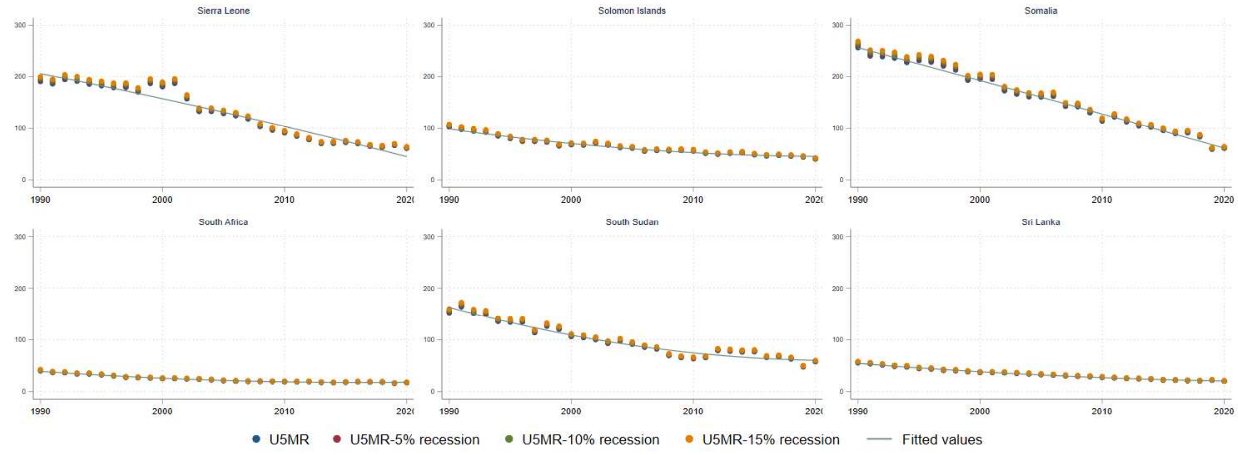

Source: Authors' elaboration

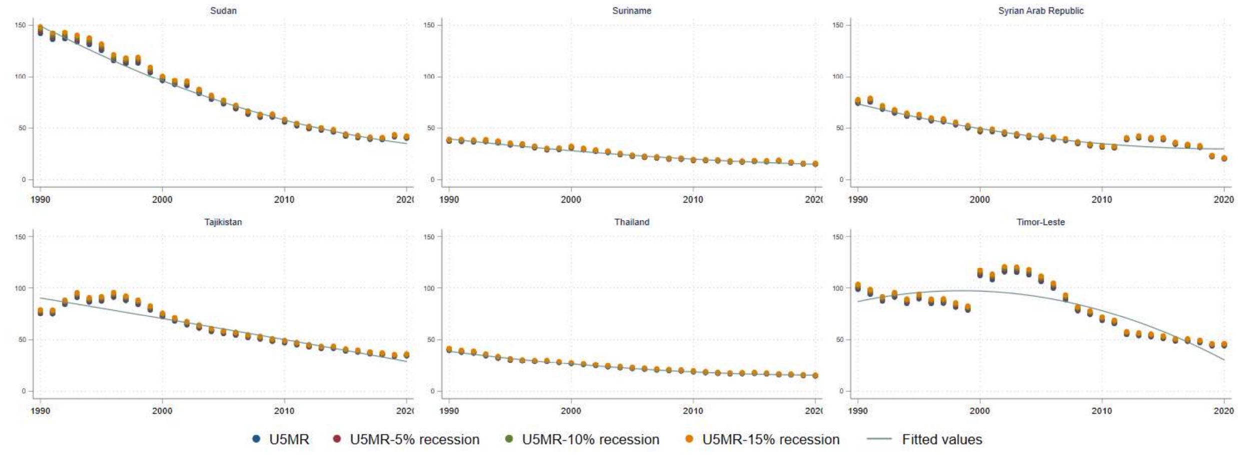

Source: Authors' elaboration

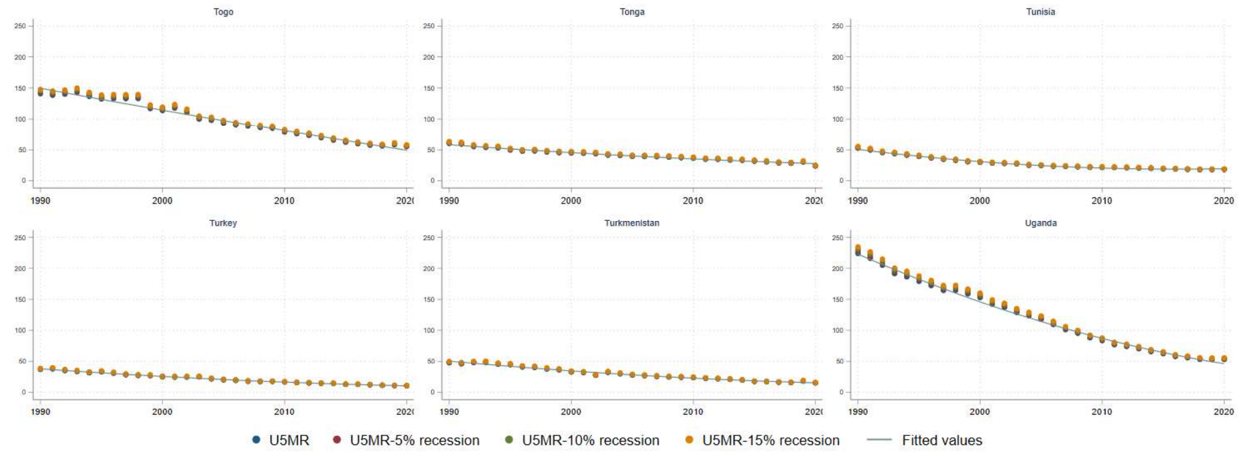

Source: Authors' elaboration

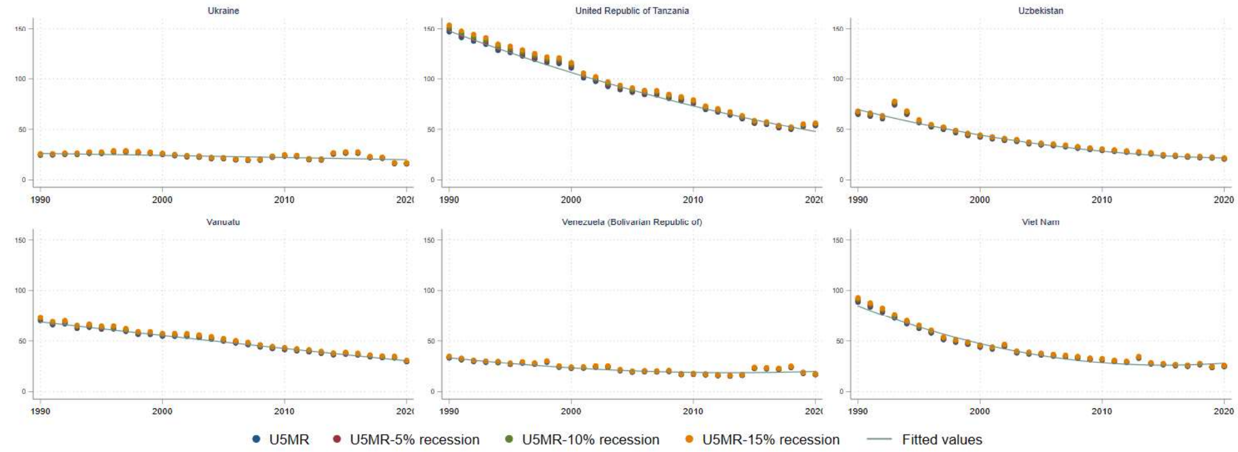

Source: Authors' elaboration

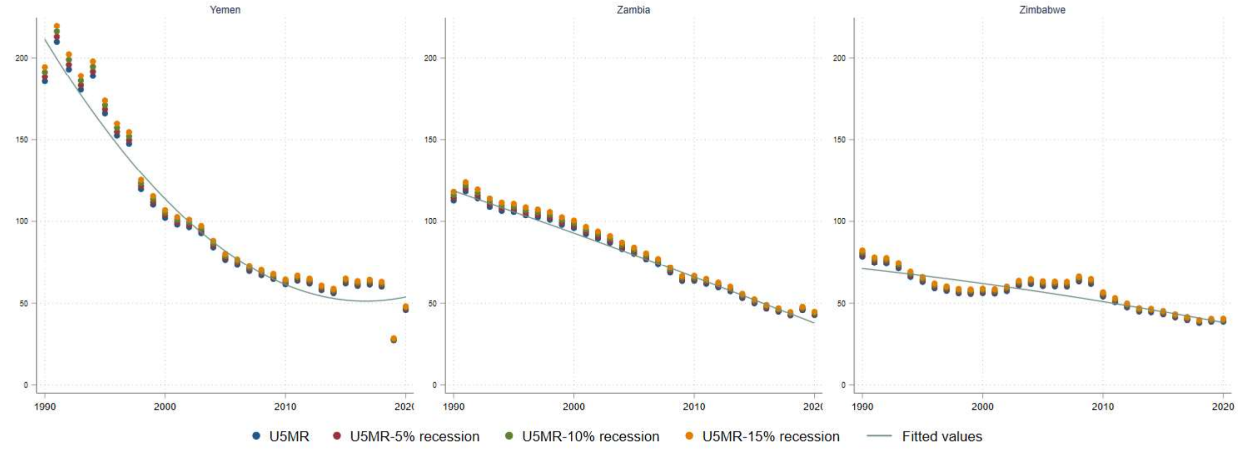

Source: Authors' elaboration

Estimations

Model 1

Appendix Table 3 Estimated effect of GDP reduction on U5M (model without controls)

Country	Under 5 deaths	95% Confidence Interval - lower bound	95% Confidence Interval - upper bound	Under-5 deaths 5% reduction on GDP	95% Confidence Interval - lower bound	95% Confidence Interval - upper bound	Incremental Deaths at 5% Recession	Under 5 deaths 10% reduction on GDP	95% Confidence Interval - lower bound	95% Confidence Interval - upper bound	Incremental Deaths at 10% Recession	Under 5 deaths 15% reduction on GDP	95% Confidence Interval - lower bound	95% Confidence Interval - upper bound	Incremental Deaths at 15% Recession
Afghanistan	279,869	113,507	690,059	286,568	116,685	703,787	6,699	293,803	120,129	718,566	13,935	301,651	123,878	734,543	21,782
Albania	3,055	1,037	8,998	3,128	1,067	9,172	73	3,207	1,099	9,360	152	3,293	1,134	9,562	238
Algeria	93,430	31,775	274,723	95,667	32,682	280,038	2,236	98,082	33,665	285,756	4,652	100,702	34,737	291,931	7,272
Angola	125,643	44,014	358,663	128,650	45,267	365,628	3,008	131,899	46,626	373,120	6,256	135,422	48,107	381,212	9,779
Argentina	48,814	15,518	153,549	49,982	15,963	156,500	1,168	51,244	16,446	159,672	2,430	52,613	16,972	163,098	3,799
Armenia	4,020	1,382	11,693	4,116	1,421	11,920	96	4,220	1,464	12,164	200	4,332	1,510	12,427	313
Azerbaijan	13,915	4,647	41,661	14,248	4,780	42,465	333	14,607	4,924	43,330	693	14,998	5,081	44,265	1,083
Bangladesh	515,704	198,110	1,342,442	528,048	203,698	1,368,869	12,345	541,381	209,755	1,397,309	25,677	555,841	216,352	1,428,043	40,137
Belarus	8,845	2,930	26,706	9,057	3,013	27,221	212	9,286	3,104	27,775	440	9,534	3,203	28,374	688
Belize	774	266	2,251	793	274	2,295	19	813	282	2,342	39	834	291	2,393	60
Benin	67,402	25,807	176,040	69,015	26,535	179,504	1,613	70,758	27,324	183,231	3,356	72,648	28,184	187,259	5,246
Bhutan	1,470	521	4,149	1,505	535	4,230	35	1,543	551	4,316	73	1,584	569	4,410	114
Bolivia (Plurinational State of)	29,709	10,691	82,555	30,420	10,995	84,164	711	31,188	11,324	85,895	1,479	32,021	11,683	87,765	2,312
Bosnia and Herzegovina	2,259	755	6,757	2,313	777	6,887	54	2,372	800	7,028	112	2,435	826	7,179	176
Botswana	3,998	1,301	12,290	4,094	1,338	12,526	96	4,197	1,379	12,781	199	4,310	1,423	13,056	311
Brazil	181,631	57,287	575,867	185,979	58,931	586,924	4,348	190,674	60,715	598,814	9,043	195,767	62,658	611,651	14,136

Bulgaria	4,484	1,452	13,851	4,591	1,493	14,118	107	4,707	1,538	14,404	223	4,833	1,587	14,714	349
Burkina Faso	149,031	59,030	376,251	152,598	60,689	383,700	3,567	156,451	62,486	391,718	7,420	160,630	64,443	400,384	11,599
Burundi	158,638	69,135	364,015	162,435	71,045	371,390	3,797	166,537	73,113	379,335	7,899	170,985	75,364	387,929	12,347
Cabo Verde	1,091	380	3,133	1,117	391	3,193	26	1,145	402	3,259	54	1,176	415	3,329	85
Cambodia	63,848	24,515	166,288	65,377	25,207	169,561	1,528	67,027	25,957	173,084	3,179	68,818	26,773	176,891	4,969
Cameroon	130,780	49,140	348,060	133,911	50,529	354,886	3,131	137,292	52,036	362,232	6,511	140,959	53,677	370,169	10,179
Central African Republic	44,619	18,710	106,402	45,687	19,231	108,535	1,068	46,840	19,796	110,832	2,222	48,091	20,410	113,316	3,473
Chad	120,242	47,260	305,927	123,120	48,589	311,974	2,878	126,229	50,030	318,483	5,987	129,600	51,598	325,519	9,358
China	1,275,963	417,772	3,897,060	1,306,506	429,731	3,972,155	30,543	1,339,493	442,703	4,052,918	63,530	1,375,271	456,838	4,140,128	99,308
Colombia	55,252	18,018	169,430	56,575	18,534	172,694	1,323	58,003	19,093	176,204	2,751	59,552	19,703	179,994	4,300
Comoros	4,052	1,531	10,723	4,149	1,575	10,934	97	4,254	1,622	11,160	202	4,368	1,673	11,405	315
Congo	19,549	6,967	54,850	20,017	7,165	55,918	468	20,522	7,380	57,066	973	21,070	7,614	58,306	1,521
Costa Rica	4,653	1,486	14,570	4,764	1,528	14,850	111	4,884	1,575	15,151	232	5,015	1,625	15,476	362
Cuba	9,095	3,005	27,532	9,313	3,090	28,063	218	9,548	3,184	28,634	453	9,803	3,285	29,251	708
Côte d'Ivoire	126,899	47,392	339,791	129,937	48,733	346,449	3,038	133,217	50,187	353,613	6,318	136,776	51,771	361,354	9,877
Dem. People's Republic of Korea	41,451	11,376	151,042	42,443	11,688	154,123	992	43,515	12,027	157,440	2,064	44,677	12,396	161,028	3,226
Democratic Republic of the Congo	906,013	376,596	2,179,685	927,701	387,097	2,223,291	21,687	951,123	398,475	2,270,244	45,110	976,528	410,858	2,321,013	70,515
Djibouti	1,882	513	6,908	1,927	527	7,047	45	1,976	542	7,198	94	2,029	559	7,360	146
Dominican Republic	15,264	4,999	46,608	15,629	5,142	47,506	365	16,024	5,297	48,472	760	16,452	5,466	49,515	1,188
Ecuador	29,711	10,029	88,014	30,422	10,316	89,715	711	31,190	10,627	91,545	1,479	32,023	10,965	93,522	2,312
Egypt	299,137	106,426	840,803	306,298	109,452	857,162	7,161	314,031	112,734	874,761	14,894	322,419	116,309	893,773	23,282
El Salvador	12,418	4,345	35,489	12,715	4,469	36,178	297	13,036	4,603	36,919	618	13,385	4,749	37,720	966
Equatorial Guinea	2,489	784	7,900	2,548	806	8,052	60	2,612	831	8,215	124	2,682	857	8,391	194
Eritrea	14,973	4,487	49,971	15,332	4,610	50,987	358	15,719	4,744	52,082	746	16,139	4,890	53,265	1,165

Eswatini	2,676	911	7,862	2,740	937	8,014	64	2,810	965	8,178	133	2,885	996	8,354	208
Ethiopia	848,857	345,676	2,084,485	869,176	355,348	2,125,991	20,319	891,121	365,829	2,170,676	42,264	914,923	377,238	2,218,982	66,066
Fiji	1,683	574	4,935	1,724	591	5,030	40	1,767	608	5,133	84	1,814	628	5,244	131
Gabon	4,373	1,402	13,639	4,478	1,442	13,901	105	4,591	1,486	14,183	218	4,713	1,534	14,488	340
Gambia	17,593	6,970	44,409	18,014	7,166	45,288	421	18,469	7,378	46,235	876	18,963	7,609	47,257	1,369
Georgia	5,092	1,740	14,907	5,214	1,789	15,195	122	5,346	1,843	15,506	254	5,489	1,902	15,841	396
Ghana	123,156	45,670	332,105	126,104	46,963	338,606	2,948	129,287	48,366	345,600	6,132	132,741	49,893	353,156	9,585
Grenada	126	41	392	129	42	399	3	133	43	407	6	136	44	416	10
Guatemala	45,402	15,946	129,272	46,489	16,400	131,783	1,087	47,663	16,892	134,484	2,261	48,936	17,429	137,402	3,534
Guinea	86,397	33,973	219,717	88,465	34,928	224,061	2,068	90,699	35,964	228,736	4,302	93,121	37,091	233,790	6,724
Guinea-Bissau	14,630	5,905	36,245	14,980	6,070	36,966	350	15,358	6,250	37,741	728	15,768	6,445	38,580	1,139
Guyana	1,504	521	4,344	1,540	535	4,428	36	1,579	552	4,519	75	1,621	569	4,617	117
Haiti	55,775	22,200	140,124	57,110	22,824	142,901	1,335	58,552	23,499	145,890	2,777	60,116	24,235	149,121	4,341
Honduras	27,161	9,889	74,599	27,811	10,170	76,055	650	28,514	10,474	77,622	1,352	29,275	10,806	79,314	2,114
India	3,259,649	1,196,249	8,882,194	3,337,676	1,230,164	9,055,765	78,027	3,421,945	1,266,939	9,242,523	162,296	3,513,347	1,306,992	9,444,286	253,698
Indonesia	469,123	161,602	1,361,848	480,353	166,210	1,388,238	11,230	492,481	171,208	1,416,624	23,357	505,635	176,653	1,447,283	36,512
Iran (Islamic Republic of)	121,985	40,322	369,037	124,905	41,476	376,155	2,920	128,059	42,727	383,811	6,074	131,480	44,090	392,078	9,494
Iraq	92,774	31,121	276,570	94,995	32,010	281,913	2,221	97,393	32,974	287,660	4,619	99,995	34,025	293,866	7,221
Jamaica	4,268	1,451	12,556	4,370	1,492	12,798	102	4,481	1,537	13,060	213	4,600	1,586	13,342	332
Jordan	23,325	8,196	66,377	23,883	8,429	67,666	558	24,486	8,683	69,053	1,161	25,140	8,958	70,551	1,815
Kazakhstan	24,274	7,667	76,850	24,855	7,887	78,326	581	25,483	8,126	79,913	1,209	26,163	8,386	81,626	1,889
Kenya	250,460	96,008	653,386	256,455	98,716	666,243	5,995	262,930	101,653	680,080	12,470	269,953	104,850	695,033	19,493
Kiribati	445	165	1,201	455	169	1,225	11	467	174	1,250	22	479	180	1,277	35
Kyrgyzstan	28,103	10,847	72,814	28,776	11,153	74,249	673	29,503	11,484	75,793	1,399	30,291	11,845	77,461	2,187
Lao People's Democratic Republic	23,809	8,848	64,072	24,379	9,098	65,327	570	24,995	9,370	66,676	1,185	25,662	9,666	68,135	1,853
Lebanon	9,257	3,074	27,878	9,478	3,162	28,416	222	9,718	3,257	28,995	461	9,977	3,361	29,620	720
Lesotho	8,308	3,139	21,990	8,507	3,228	22,422	199	8,722	3,324	22,886	414	8,955	3,429	23,388	647
Liberia	37,345	15,202	91,742	38,239	15,627	93,569	894	39,204	16,088	95,535	1,859	40,252	16,590	97,661	2,907
Libya	9,768	3,216	29,666	10,002	3,308	30,238	234	10,254	3,408	30,853	486	10,528	3,517	31,517	760

Madagascar	219,003	89,969	533,099	224,246	92,483	543,734	5,242	229,907	95,207	555,186	10,904	236,048	98,172	567,566	17,045
Malawi	151,606	61,999	370,722	155,235	63,732	378,111	3,629	159,154	65,611	386,066	7,548	163,405	67,656	394,666	11,800
Malaysia	32,324	10,150	102,942	33,098	10,441	104,918	774	33,934	10,757	107,043	1,609	34,840	11,102	109,336	2,516
Maldives	525	171	1,612	537	176	1,643	13	551	181	1,677	26	566	187	1,713	41
Mali	155,656	61,717	392,581	159,382	63,451	400,354	3,726	163,406	65,330	408,722	7,750	167,771	67,375	417,766	12,115
Mauritania	20,460	7,593	55,134	20,950	7,808	56,213	490	21,479	8,041	57,374	1,019	22,053	8,295	58,629	1,592
Mexico	142,829	45,385	449,488	146,248	46,687	458,124	3,419	149,940	48,099	467,411	7,111	153,945	49,638	477,439	11,116
Micronesia (Fed. States of)	294	105	824	301	108	840	7	309	111	858	15	317	115	876	23
Mongolia	7,499	2,587	21,739	7,678	2,660	22,161	180	7,872	2,740	22,614	373	8,083	2,828	23,103	584
Montenegro	543	177	1,670	556	182	1,702	13	570	187	1,736	27	586	193	1,774	42
Morocco	73,082	25,667	208,088	74,831	26,397	212,130	1,749	76,720	27,190	216,478	3,639	78,769	28,053	221,175	5,688
Mozambique	251,430	101,771	621,169	257,449	104,622	633,521	6,019	263,949	107,710	646,819	12,519	270,999	111,073	661,194	19,569
Myanmar	143,552	53,958	381,912	146,988	55,484	389,403	3,436	150,699	57,138	397,463	7,147	154,724	58,939	406,173	11,173
Namibia	5,595	1,864	16,792	5,729	1,917	17,116	134	5,873	1,975	17,464	279	6,030	2,038	17,841	435
Nepal	115,550	45,727	291,991	118,316	47,012	297,771	2,766	121,303	48,404	303,992	5,753	124,543	49,920	310,716	8,993
Nicaragua	18,802	6,933	50,993	19,252	7,129	51,991	450	19,738	7,342	53,064	936	20,266	7,574	54,223	1,463
Niger	243,175	99,106	596,677	248,996	101,878	608,559	5,821	255,283	104,883	621,353	12,108	262,102	108,153	635,183	18,926
Nigeria	856,896	308,792	2,377,887	877,408	317,562	2,424,234	20,512	899,561	327,073	2,474,096	42,665	923,588	337,432	2,527,962	66,692
North Macedonia	1,969	663	5,846	2,016	682	5,959	47	2,067	702	6,081	98	2,122	725	6,212	153
Pakistan	1,000,517	383,952	2,607,185	1,024,467	394,784	2,658,500	23,950	1,050,333	406,526	2,713,725	49,815	1,078,388	419,312	2,773,403	77,870
Papua New Guinea	27,906	10,053	77,463	28,573	10,338	78,973	668	29,295	10,648	80,597	1,389	30,077	10,985	82,351	2,172
Paraguay	12,496	4,218	37,016	12,795	4,339	37,732	299	13,118	4,469	38,501	622	13,468	4,612	39,332	973
Peru	46,124	15,302	139,025	47,228	15,740	141,707	1,104	48,420	16,215	144,592	2,296	49,713	16,732	147,708	3,590
Philippines	242,622	85,832	685,817	248,429	88,274	699,151	5,808	254,702	90,923	713,495	12,080	261,505	93,808	728,990	18,883
Republic of Moldova	4,440	1,559	12,649	4,547	1,603	12,894	106	4,661	1,651	13,159	221	4,786	1,704	13,444	346
Russian Federation	114,014	35,818	362,922	116,744	36,846	369,888	2,729	119,691	37,962	377,378	5,677	122,888	39,177	385,465	8,874
Rwanda	78,646	31,000	199,522	80,528	31,871	203,469	1,883	82,561	32,816	207,716	3,916	84,767	33,844	212,307	6,121

Saint Lucia	150	48	466	154	50	475	4	157	51	485	7	162	53	495	12
Saint Vincent and the Grenadines	122	40	369	125	41	376	3	128	43	384	6	131	44	392	9
Samoa	558	194	1,607	571	199	1,638	13	586	205	1,672	28	601	212	1,708	43
Sao Tome and Principe	1,081	411	2,842	1,107	423	2,898	26	1,135	435	2,958	54	1,165	449	3,023	84
Senegal	83,013	31,185	220,974	85,000	32,067	225,308	1,987	87,146	33,023	229,971	4,133	89,473	34,064	235,010	6,461
Serbia	6,675	2,205	20,203	6,834	2,268	20,593	160	7,007	2,337	21,012	332	7,194	2,411	21,464	519
Sierra Leone	62,849	25,894	152,541	64,353	26,617	155,586	1,504	65,978	27,401	158,865	3,129	67,740	28,254	162,410	4,892
Solomon Islands	3,296	1,240	8,764	3,375	1,275	8,936	79	3,461	1,313	9,121	164	3,553	1,354	9,321	257
Somalia	96,456	30,164	308,437	98,764	30,994	314,724	2,309	101,258	31,892	321,494	4,802	103,963	32,870	328,817	7,507
South Africa	86,983	28,438	266,051	89,065	29,252	271,177	2,082	91,314	30,136	276,690	4,331	93,753	31,098	282,643	6,770
South Sudan	75,191	29,915	188,996	76,991	30,755	192,741	1,800	78,935	31,665	196,772	3,744	81,043	32,656	201,129	5,852
Sri Lanka	34,005	11,786	98,117	34,819	12,122	100,020	814	35,698	12,486	102,066	1,693	36,652	12,883	104,277	2,647
Sudan	183,077	67,615	495,706	187,459	69,530	505,402	4,382	192,192	71,608	515,835	9,115	197,325	73,870	527,107	14,249
Suriname	759	246	2,337	777	253	2,382	18	797	261	2,431	38	818	269	2,483	59
Syrian Arab Republic	36,882	10,790	126,062	37,764	11,092	128,576	883	38,718	11,419	131,282	1,836	39,752	11,775	134,206	2,870
Tajikistan	51,143	19,807	132,056	52,367	20,365	134,659	1,224	53,690	20,970	137,461	2,546	55,124	21,629	140,489	3,980
Thailand	59,407	19,765	178,558	60,829	20,330	182,004	1,422	62,364	20,943	185,711	2,958	64,030	21,611	189,714	4,624
Timor-Leste	7,193	2,819	18,350	7,365	2,899	18,713	172	7,551	2,985	19,103	358	7,753	3,078	19,525	560
Togo	56,223	22,542	140,232	57,569	23,174	143,015	1,346	59,023	23,859	146,011	2,799	60,599	24,605	149,249	4,376
Tonga	245	85	709	251	87	723	6	257	90	738	12	264	92	754	19
Tunisia	19,417	6,657	56,635	19,882	6,847	57,732	465	20,383	7,053	58,912	967	20,928	7,277	60,186	1,511
Turkey	72,774	22,398	236,452	74,516	23,042	240,982	1,742	76,397	23,740	245,853	3,623	78,438	24,501	251,112	5,664
Turkmenistan	10,168	3,337	30,985	10,411	3,432	31,582	243	10,674	3,536	32,225	506	10,959	3,649	32,918	791
Uganda	308,549	120,516	789,954	315,934	123,908	805,551	7,386	323,911	127,585	822,339	15,362	332,563	131,589	840,482	24,014
Ukraine	48,486	17,165	136,963	49,647	17,653	139,626	1,161	50,901	18,183	142,491	2,414	52,260	18,760	145,586	3,774

United Republic of Tanzania	383,628	149,719	982,974	392,811	153,934	1,002,380	9,183	402,728	158,503	1,023,266	19,101	413,486	163,477	1,045,838	29,858
Uzbekistan	89,151	32,296	246,099	91,285	33,212	250,899	2,134	93,590	34,207	256,064	4,439	96,090	35,289	261,643	6,939
Vanuatu	992	353	2,787	1,016	363	2,842	24	1,041	374	2,900	49	1,069	386	2,963	77
Venezuela (Bolivarian Republic of)	39,993	10,758	148,677	40,950	11,057	151,662	957	41,984	11,381	154,876	1,991	43,105	11,734	158,350	3,113
Viet Nam	226,042	83,356	612,969	231,452	85,718	624,956	5,411	237,296	88,279	637,854	11,254	243,634	91,069	651,789	17,593
Yemen	186,570	74,594	466,635	191,036	76,687	475,890	4,466	195,859	78,956	485,854	9,289	201,091	81,425	496,623	14,521
Zambia	88,936	33,110	238,893	91,065	34,047	243,572	2,129	93,365	35,063	248,606	4,428	95,858	36,170	254,045	6,922
Zimbabwe	72,349	27,585	189,754	74,081	28,364	193,484	1,732	75,951	29,208	197,499	3,602	77,980	30,127	201,837	5,631
**Total**	**16,829,307**			**17,232,154**			**402,847**	**17,667,229**			**837,922**	**18,139,129**			**1,309,822**

Source: Authors' elaboration

Appendix Table 4: Estimated Under-5 Lives Lost from 2020 Recessions Scaled from 5% to 15%.

	Scenario 1 No Recession	Scenario 2 5% Recession	Scenario 3 10% Recession	Scenario 4 15% Recession
Country	Under-5 mortality rate per 100	Under-5 mortality rate per 100	Under-5 mortality rate per 100	Under-5 mortality rate per 100
Burundi	9.68	9.82	9.97	10.14
Niger	9.64	9.78	9.93	10.09
Democratic Republic of the Congo	8.77	8.9	9.04	9.18
Central African Republic	7.33	7.44	7.55	7.68
Mali	7.15	7.26	7.37	7.49
Chad	6.54	6.63	6.73	6.84
Mozambique	6.37	6.46	6.56	6.67
Burkina Faso	6.3	6.4	6.5	6.6
Somalia	6.14	6.23	6.32	6.43
Sierra Leone	6.13	6.22	6.31	6.42

Source: Authors' elaboration

Model 2

Appendix Table 4 Estimated effect of GDP reduction on U5M (model with controls)

Country	Under 5 deaths	95% Confidence Interval - lower bound	95% Confidence Interval - upper bound	Under 5 deaths 5% reduction on GDP	95% Confidence Interval - lower bound	95% Confidence Interval - upper bound	Incremental Deaths at 5% Recession	Under 5 deaths 10% reduction on GDP	95% Confidenc e Interval - lower bound	95% Confidence Interval - upper bound	Incremental Deaths at 10% Recession	Under 5 deaths 15% reduction on GDP	95% Confidenc e Interval - lower bound	95% Confidence Interval - upper bound	Incremental Deaths at 15% Recession
Afghanistan	323,613	120,308	870,476	328,370	122,533	879,983	4,757	333,460	124,920	890,133	9,848	338,927	127,492	901,013	15,315
Albania	2,394	752	7,622	2,429	766	7,701	35	2,466	781	7,785	73	2,507	798	7,875	113
Algeria	110,808	34,603	354,838	112,437	35,262	358,513	1,629	114,180	35,971	362,432	3,372	116,052	36,735	366,626	5,244
Angola	235,624	75,729	733,123	239,088	77,167	740,769	3,464	242,794	78,712	748,924	7,170	246,775	80,377	757,656	11,151
Argentina	53,650	15,722	183,073	54,438	16,025	184,938	789	55,282	16,349	186,926	1,633	56,189	16,700	189,054	2,539
Armenia	3,166	1,000	10,018	3,212	1,019	10,123	47	3,262	1,040	10,234	96	3,316	1,062	10,353	150
Azerbaijan	12,099	3,740	39,138	12,277	3,812	39,542	178	12,467	3,888	39,971	368	12,671	3,971	40,431	573
Bangladesh	435,117	153,268	1,235,269	441,513	156,135	1,248,500	6,396	448,358	159,212	1,262,622	13,241	455,709	162,528	1,277,750	20,592
Belarus	7,379	2,244	24,266	7,487	2,287	24,517	108	7,603	2,333	24,784	225	7,728	2,382	25,070	349
Belize	749	237	2,367	760	241	2,392	11	772	246	2,418	23	784	252	2,446	35
Benin	98,813	34,464	283,314	100,266	35,109	286,346	1,453	101,820	35,801	289,583	3,007	103,489	36,547	293,050	4,676
Bhutan	1,332	435	4,078	1,352	444	4,120	20	1,373	453	4,166	41	1,395	462	4,214	63
Bolivia (Plurinational State of)	29,494	9,774	88,998	29,928	9,959	89,932	434	30,391	10,158	90,929	898	30,890	10,372	91,996	1,396
Bosnia and Herzegovina	1,702	521	5,557	1,727	531	5,614	25	1,753	542	5,676	52	1,782	553	5,741	81
Botswana	5,091	1,534	16,899	5,166	1,563	17,072	75	5,246	1,595	17,257	155	5,332	1,629	17,455	241
Brazil	182,846	53,523	624,640	185,534	54,554	630,985	2,688	188,410	55,662	637,751	5,564	191,499	56,857	644,989	8,653
Bulgaria	3,723	1,113	12,449	3,778	1,135	12,577	55	3,836	1,158	12,713	113	3,899	1,182	12,858	176
Burkina Faso	218,887	79,526	602,461	222,105	81,005	608,984	3,218	225,548	82,591	615,948	6,661	229,246	84,300	623,410	10,359
Burundi	198,778	78,436	503,757	201,700	79,854	509,463	2,922	204,827	81,376	515,559	6,049	208,185	83,013	522,098	9,407

Cabo Verde	1,146	367	3,582	1,163	374	3,620	17	1,181	381	3,660	35	1,200	389	3,702	54
Cambodia	59,438	20,803	169,830	60,312	21,191	171,659	874	61,247	21,607	173,611	1,809	62,251	22,056	175,702	2,813
Cameroon	183,530	63,386	531,403	186,228	64,575	537,061	2,698	189,115	65,852	543,100	5,585	192,215	67,229	549,568	8,685
Central African Republic	54,126	20,663	141,779	54,922	21,043	143,347	796	55,773	21,449	145,023	1,647	56,688	21,887	146,819	2,561
Chad	191,519	68,658	534,235	194,334	69,935	540,011	2,815	197,347	71,306	546,177	5,828	200,582	72,783	552,783	9,064
China	1,235,908	372,924	4,095,918	1,254,076	380,064	4,138,001	18,169	1,273,517	387,734	4,182,878	37,609	1,294,396	396,005	4,230,905	58,489
Colombia	51,670	15,615	170,968	52,429	15,915	172,715	760	53,242	16,237	174,578	1,572	54,115	16,585	176,571	2,445
Comoros	5,310	1,842	15,306	5,388	1,877	15,468	78	5,471	1,914	15,642	162	5,561	1,954	15,827	251
Congo	29,476	9,685	89,706	29,909	9,869	90,644	433	30,373	10,066	91,644	897	30,871	10,279	92,715	1,395
Costa Rica	4,588	1,351	15,584	4,656	1,377	15,743	67	4,728	1,405	15,912	140	4,806	1,435	16,093	217
Cuba	8,100	2,462	26,655	8,219	2,509	26,929	119	8,347	2,559	27,222	246	8,484	2,614	27,535	383
Côte d'Ivoire	185,002	63,376	540,040	187,721	64,569	545,761	2,720	190,631	65,850	551,866	5,630	193,757	67,230	558,404	8,755
Dem. People's Republic of Korea	37,542	10,563	133,430	38,094	10,757	134,899	552	38,684	10,966	136,467	1,142	39,318	11,191	138,146	1,777
Democratic Republic of the Congo	1,388,004	524,706	3,671,682	1,408,409	534,338	3,712,285	20,405	1,430,241	544,670	3,755,652	42,237	1,453,691	555,796	3,802,143	65,687
Djibouti	2,076	583	7,397	2,107	593	7,478	31	2,139	605	7,563	63	2,174	618	7,655	98
Dominican Republic	16,310	4,937	53,880	16,549	5,032	54,433	240	16,806	5,133	55,022	496	17,081	5,243	55,653	772
Ecuador	30,859	9,623	98,961	31,312	9,806	99,980	454	31,798	10,004	101,068	939	32,319	10,217	102,231	1,460
Egypt	339,094	110,682	1,038,877	344,079	112,777	1,049,774	4,985	349,413	115,027	1,061,399	10,319	355,141	117,452	1,073,846	16,047
El Salvador	11,381	3,664	35,348	11,548	3,734	35,718	167	11,727	3,808	36,112	346	11,919	3,889	36,534	539
Equatorial Guinea	4,411	1,284	15,155	4,476	1,309	15,309	65	4,546	1,335	15,473	134	4,620	1,364	15,649	209
Eritrea	19,506	6,022	63,177	19,793	6,133	63,871	287	20,099	6,253	64,611	594	20,429	6,381	65,405	923
Eswatini	3,328	1,046	10,595	3,377	1,066	10,705	49	3,430	1,087	10,821	101	3,486	1,110	10,946	158
Ethiopia	992,985	369,329	2,669,755	1,007,582	376,148	2,698,999	14,598	1,023,202	383,463	2,730,228	30,217	1,039,977	391,343	2,763,698	46,993
Fiji	1,887	593	6,005	1,915	604	6,067	28	1,944	616	6,133	57	1,976	629	6,204	89

Gabon	6,873	2,046	23,087	6,974	2,085	23,322	101	7,082	2,128	23,573	209	7,198	2,173	23,841	325
Gambia	25,061	9,085	69,132	25,430	9,254	69,879	368	25,824	9,436	70,676	763	26,247	9,631	71,531	1,186
Georgia	4,272	1,350	13,518	4,334	1,376	13,657	63	4,402	1,403	13,806	130	4,474	1,433	13,966	202
Ghana	156,000	53,051	458,733	158,294	54,049	463,595	2,293	160,748	55,121	468,784	4,747	163,383	56,276	474,341	7,383
Grenada	128	38	430	130	39	435	2	132	40	439	4	134	41	444	6
Guatemala	47,406	15,369	146,228	48,103	15,661	147,751	697	48,848	15,974	149,376	1,443	49,649	16,312	151,115	2,243
Guinea	117,136	42,447	323,242	118,858	43,239	326,721	1,722	120,700	44,089	330,436	3,564	122,679	45,004	334,415	5,543
Guinea-Bissau	18,386	6,779	49,865	18,656	6,905	50,409	270	18,946	7,039	50,989	559	19,256	7,184	51,612	870
Guyana	1,467	469	4,590	1,488	478	4,638	22	1,511	487	4,689	45	1,536	497	4,743	69
Haiti	49,167	17,882	135,189	49,890	18,213	136,658	723	50,663	18,569	138,226	1,496	51,494	18,953	139,907	2,327
Honduras	24,945	8,339	74,620	25,312	8,497	75,405	367	25,705	8,666	76,244	759	26,126	8,848	77,141	1,181
India	2,929,298	986,082	8,701,895	2,972,361	1,004,659	8,793,951	43,063	3,018,437	1,024,604	8,892,182	89,139	3,067,926	1,046,101	8,997,378	138,628
Indonesia	461,840	147,090	1,450,106	468,629	149,891	1,465,156	6,789	475,893	152,899	1,481,208	14,054	483,696	156,142	1,498,392	21,856
Iran (Islamic Republic of)	125,719	38,512	410,401	127,567	39,248	414,631	1,848	129,544	40,038	419,143	3,826	131,668	40,891	423,973	5,950
Iraq	121,740	37,598	394,186	123,529	38,317	398,246	1,790	125,444	39,089	402,575	3,705	127,501	39,922	407,209	5,761
Jamaica	3,971	1,238	12,740	4,030	1,262	12,872	58	4,092	1,287	13,013	121	4,159	1,314	13,163	188
Jordan	24,669	7,962	76,434	25,031	8,113	77,233	363	25,419	8,275	78,086	751	25,836	8,449	78,999	1,167
Kazakhstan	25,519	7,467	87,216	25,894	7,610	88,105	375	26,295	7,764	89,052	777	26,726	7,931	90,066	1,208
Kenya	277,566	97,930	786,710	281,646	99,765	795,117	4,080	286,012	101,734	804,090	8,446	290,701	103,855	813,702	13,136
Kiribati	508	173	1,494	515	176	1,510	7	523	179	1,527	15	532	183	1,545	24
Kyrgyzstan	22,676	8,028	64,047	23,009	8,178	64,736	333	23,366	8,339	65,473	690	23,749	8,512	66,261	1,073
Lao People's Democratic Republic	22,522	7,702	65,854	22,853	7,847	66,552	331	23,207	8,003	67,298	685	23,587	8,170	68,096	1,066
Lebanon	9,088	2,782	29,693	9,222	2,835	29,999	134	9,365	2,892	30,326	277	9,518	2,953	30,676	430
Lesotho	8,928	3,091	25,792	9,060	3,149	26,066	131	9,200	3,211	26,359	272	9,351	3,278	26,672	423
Liberia	44,683	16,597	120,296	45,340	16,903	121,616	657	46,042	17,232	123,025	1,360	46,797	17,585	124,536	2,115
Libya	9,972	3,033	32,782	10,119	3,091	33,120	147	10,276	3,154	33,480	303	10,444	3,221	33,865	472
Madagascar	245,912	91,406	661,589	249,528	93,087	668,881	3,615	253,396	94,890	676,669	7,483	257,550	96,833	685,016	11,638
Malawi	173,658	64,879	464,823	176,211	66,074	469,933	2,553	178,942	67,356	475,391	5,284	181,876	68,737	481,241	8,218
Malaysia	33,845	9,852	116,269	34,343	10,042	117,453	498	34,875	10,245	118,715	1,030	35,447	10,465	120,066	1,602

Maldives	529	159	1,760	537	162	1,778	8	545	166	1,797	16	554	169	1,817	25
Mali	257,889	93,417	711,934	261,680	95,149	719,675	3,791	265,737	97,008	727,938	7,848	270,094	99,011	736,794	12,205
Mauritania	28,358	9,644	83,384	28,774	9,825	84,268	417	29,221	10,020	85,211	863	29,700	10,230	86,221	1,342
Mexico	151,002	44,437	513,117	153,221	45,292	518,343	2,220	155,597	46,210	523,914	4,595	158,148	47,201	529,875	7,146
Micronesia (Fed. States of)	320	105	973	325	107	983	5	330	109	994	10	335	112	1,006	15
Mongolia	8,259	2,632	25,918	8,381	2,682	26,187	121	8,511	2,736	26,474	251	8,650	2,794	26,781	391
Montenegro	457	137	1,529	464	139	1,545	7	471	142	1,562	14	479	145	1,580	22
Morocco	71,179	22,985	220,422	72,225	23,421	222,725	1,046	73,345	23,890	225,181	2,166	74,547	24,395	227,811	3,369
Mozambique	328,481	121,560	887,628	333,310	123,805	897,341	4,829	338,476	126,214	907,714	9,996	344,026	128,809	918,831	15,545
Myanmar	123,623	42,782	357,219	125,441	43,585	361,022	1,817	127,385	44,447	365,082	3,762	129,474	45,377	369,430	5,850
Namibia	7,403	2,282	24,016	7,512	2,326	24,262	109	7,628	2,373	24,525	225	7,753	2,423	24,806	350
Nepal	88,023	32,010	242,046	89,317	32,605	244,667	1,294	90,701	33,244	247,465	2,679	92,188	33,932	250,463	4,166
Nicaragua	16,495	5,553	48,995	16,738	5,658	49,515	242	16,997	5,770	50,069	502	17,276	5,891	50,663	781
Niger	461,338	171,118	1,243,775	468,120	174,273	1,257,434	6,782	475,377	177,657	1,272,020	14,039	483,171	181,302	1,287,654	21,833
Nigeria	1,503,219	497,646	4,540,714	1,525,317	507,077	4,588,238	22,098	1,548,962	517,205	4,638,937	45,743	1,574,358	528,124	4,693,221	71,139
North Macedonia	1,505	469	4,825	1,527	478	4,875	22	1,551	488	4,928	46	1,576	498	4,985	71
Pakistan	1,054,683	371,239	2,996,334	1,070,187	378,185	3,028,413	15,505	1,086,777	385,641	3,062,651	32,094	1,104,595	393,676	3,099,329	49,912
Papua New Guinea	32,868	10,947	98,689	33,351	11,154	99,722	483	33,868	11,377	100,823	1,000	34,424	11,617	102,002	1,555
Paraguay	12,704	3,950	40,860	12,891	4,025	41,283	187	13,091	4,106	41,733	387	13,305	4,193	42,216	601
Peru	46,296	14,179	151,157	46,976	14,451	152,711	681	47,704	14,742	154,368	1,409	48,486	15,056	156,142	2,191
Philippines	240,542	78,574	736,383	244,078	80,067	744,053	3,536	247,862	81,671	752,234	7,320	251,925	83,400	760,993	11,384
Republic of Moldova	3,073	998	9,467	3,118	1,016	9,565	45	3,166	1,037	9,671	94	3,218	1,059	9,783	145
Russian Federation	100,190	29,103	344,916	101,663	29,663	348,426	1,473	103,239	30,265	352,168	3,049	104,932	30,914	356,172	4,741
Rwanda	91,223	33,071	251,630	92,564	33,686	254,348	1,341	93,999	34,347	257,250	2,776	95,540	35,059	260,360	4,317
Saint Lucia	134	40	452	136	40	456	2	138	41	461	4	140	42	466	6

Saint Vincent and the Grenadines	115	35	378	117	36	382	2	119	37	386	4	121	37	390	5
Samoa	740	236	2,322	751	241	2,346	11	763	245	2,371	23	776	251	2,399	35
Sao Tome and Principe	1,419	495	4,064	1,440	505	4,107	21	1,462	515	4,154	43	1,486	525	4,203	67
Senegal	114,939	39,655	333,149	116,629	40,400	336,690	1,690	118,437	41,200	340,469	3,498	120,379	42,062	344,516	5,439
Serbia	5,194	1,579	17,091	5,271	1,609	17,269	76	5,352	1,641	17,458	158	5,440	1,676	17,660	246
Sierra Leone	71,002	26,707	188,764	72,046	27,198	190,842	1,044	73,163	27,726	193,062	2,161	74,362	28,294	195,442	3,360
Solomon Islands	4,201	1,453	12,150	4,263	1,480	12,279	62	4,329	1,509	12,416	128	4,400	1,541	12,563	199
Somalia	173,482	53,816	559,233	176,032	54,802	565,442	2,550	178,761	55,859	572,072	5,279	181,691	56,998	579,178	8,210
South Africa	99,249	29,951	328,878	100,708	30,525	332,249	1,459	102,269	31,142	335,844	3,020	103,946	31,807	339,691	4,697
South Sudan	97,763	35,661	268,011	99,200	36,325	270,904	1,437	100,737	37,038	273,993	2,975	102,389	37,805	277,302	4,627
Sri Lanka	33,501	10,656	105,320	33,994	10,859	106,418	492	34,521	11,076	107,589	1,019	35,087	11,311	108,842	1,585
Sudan	256,024	86,492	757,851	259,787	88,122	765,863	3,764	263,814	89,872	774,412	7,791	268,140	91,758	783,567	12,116
Suriname	809	243	2,697	821	247	2,725	12	834	252	2,754	25	847	258	2,786	38
Syrian Arab Republic	39,592	11,602	135,111	40,174	11,820	136,548	582	40,797	12,054	138,082	1,205	41,466	12,306	139,724	1,874
Tajikistan	46,998	16,668	132,524	47,689	16,979	133,949	691	48,429	17,312	135,471	1,430	49,223	17,672	137,100	2,224
Thailand	52,894	16,314	171,499	53,672	16,626	173,261	778	54,504	16,962	175,139	1,610	55,397	17,323	177,150	2,503
Timor-Leste	7,888	2,833	21,967	8,004	2,886	22,204	116	8,129	2,942	22,458	240	8,262	3,003	22,729	373
Togo	67,490	24,571	185,380	68,482	25,025	187,402	992	69,544	25,513	189,562	2,054	70,684	26,039	191,876	3,194
Tonga	298	94	940	302	96	950	4	307	98	960	9	312	100	971	14
Tunisia	18,764	5,945	59,217	19,039	6,059	59,830	276	19,335	6,181	60,484	571	19,652	6,312	61,184	888
Turkey	68,971	19,652	242,060	69,985	20,031	244,522	1,014	71,070	20,437	247,147	2,099	72,235	20,876	249,955	3,264
Turkmenistan	10,026	3,057	32,882	10,174	3,116	33,219	147	10,331	3,179	33,580	305	10,501	3,246	33,965	474
Uganda	415,087	149,123	1,155,405	421,189	151,906	1,167,830	6,102	427,718	154,893	1,181,093	12,631	434,731	158,111	1,195,303	19,644
Ukraine	33,813	10,979	104,134	34,310	11,187	105,228	497	34,842	11,410	106,395	1,029	35,413	11,650	107,645	1,600
United Republic of Tanzania	523,317	187,417	1,461,240	531,010	190,917	1,476,931	7,693	539,241	194,674	1,493,681	15,925	548,083	198,723	1,511,625	24,766

Uzbekistan	71,320	23,685	214,757	72,368	24,132	217,024	1,048	73,490	24,611	219,441	2,170	74,695	25,128	222,031	3,375
Vanuatu	1,234	406	3,753	1,252	413	3,793	18	1,272	422	3,835	38	1,292	431	3,879	58
Venezuela (Bolivarian Republic of)	39,457	10,678	145,805	40,037	10,877	147,375	580	40,658	11,090	149,051	1,201	41,324	11,321	150,846	1,867
Viet Nam	193,749	65,629	571,977	196,597	66,863	578,053	2,848	199,644	68,187	584,536	5,896	202,918	69,614	591,481	9,169
Yemen	188,879	69,331	514,566	191,656	70,618	520,149	2,777	194,627	71,999	526,110	5,748	197,818	73,487	532,498	8,939
Zambia	125,984	43,264	366,868	127,836	44,079	370,745	1,852	129,818	44,955	374,883	3,834	131,946	45,898	379,313	5,962
Zimbabwe	81,231	28,492	231,589	82,425	29,026	234,066	1,194	83,703	29,598	236,710	2,472	85,075	30,215	239,542	3,844
**Total**	**19,250,634**			**19,533,631**			**282,996**	**19,836,436**			**585,802**	**20,161,661**			**911,026**

Source: Authors' elaboration

Sensitivity analysis

Appendix Table 5 Sensitivity analysis of incremental deaths (95% Confidence Intervals)

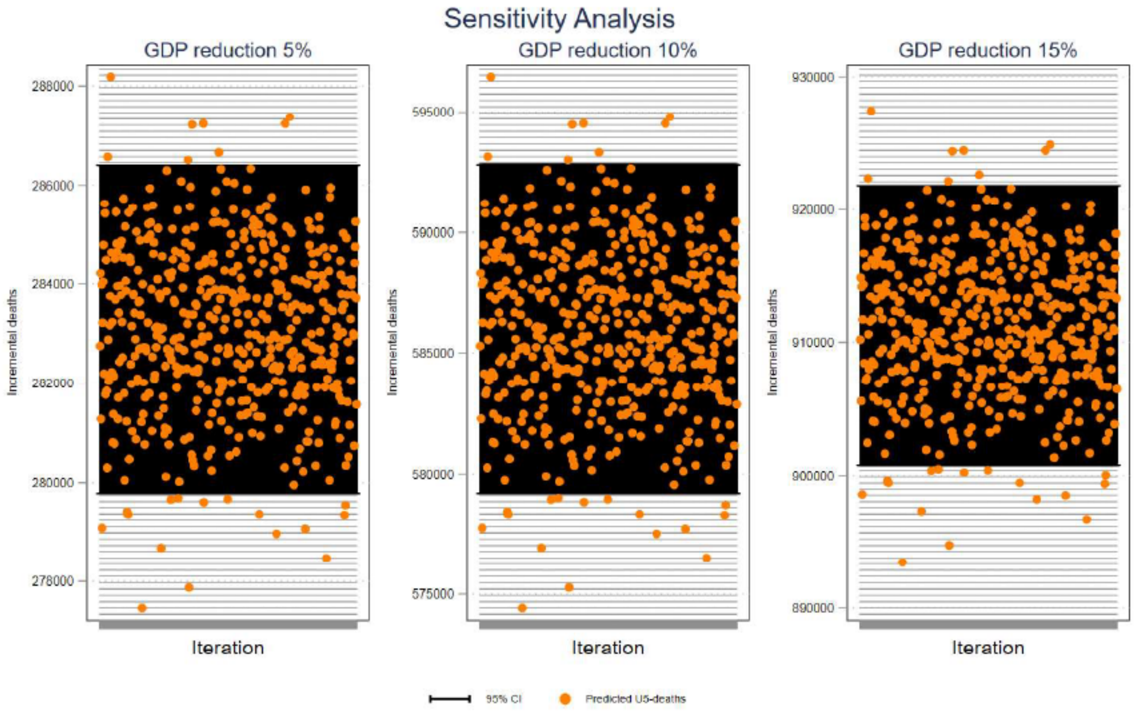

Source: Authors' elaboration

Figure 1

Click here to access/download;Figure;Figure1.tif

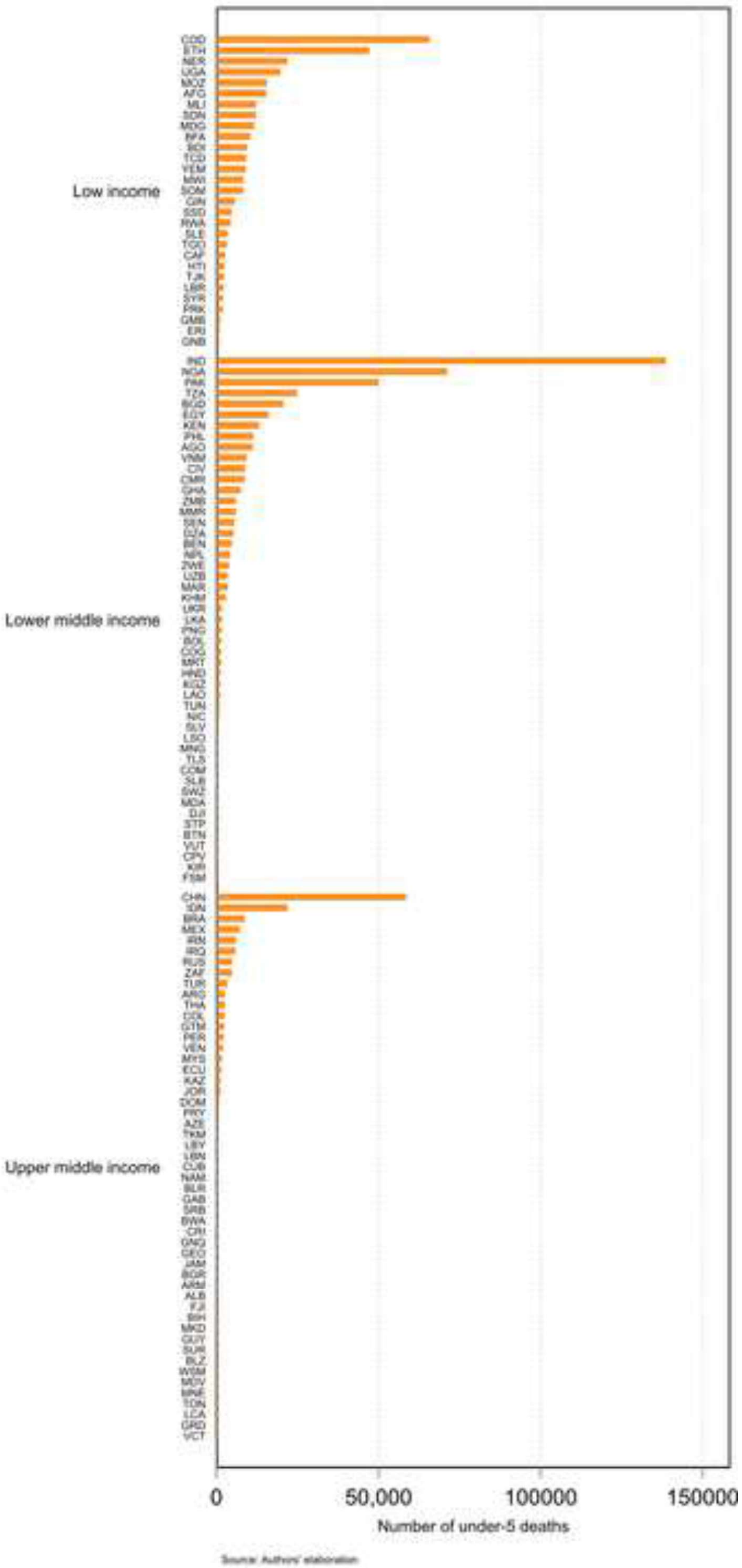

Supplement: Attachment — Submitted filename: PLoS Review_Redacted.pdf [file pone.0263245.s008.pdf]
