# Supplementary material for: Estimated impact of the 2020 economic downturn on under-5 mortality for 129 countries
Source: PLoS One. 2022 Feb 23;17(2):e0263245. doi: 10.1371/journal.pone.0263245 (PMC8865697; doi:10.1371/journal.pone.0263245)
Supplement: Attachment — Submitted filename: REVIEWERS COMMENTS.docx [file pone.0263245.s010.docx]

Title of Paper: Estimated impact of the 2020 economic downturn on under-5 mortality for 129 countries

Generally, the article is well revised but it will require editing. The following issues should be revised;

1. Recessions of under-five mortality lead to… This should be written as recession of under-five mortality led to…
2. PROTENTIAL BURDEN should be written as potential burden
3. Estimates of uncertainty- authors should delete the empty brackets ().
4. LMIC countries. This should be LMIC but not LMIC countries.
