# Supplementary material for: Estimated impact of the 2020 economic downturn on under-5 mortality for 129 countries
Source: PLoS One. 2022 Feb 23;17(2):e0263245. doi: 10.1371/journal.pone.0263245 (PMC8865697; doi:10.1371/journal.pone.0263245)
Supplement: Attachment — Submitted filename: Response to reviewers.docx [file pone.0263245.s011.docx]

*Response to reviewers*

*Reviewer 3*

1. *Recessions of under-five mortality lead to… This should be written as recession of under-five mortality led to…*

We have not been able to find the text “Recessions of under-five mortality **lead to**…” in the manuscript

2. *PROTENTIAL BURDEN should be written as potential burden*

We apologize for this typo, and we have edited it.

3. *Estimates of uncertainty- authors should delete the empty brackets ().*

We have not been able to find the empty brackets () in the **Estimates of uncertainty** sub-section of the manuscript.

4. *LMIC countries. This should be LMIC but not LMIC countries.*

We apologize for this oversight, and we have edited it.
